# Supplementary material for: Genetic and Epigenetic Fine Mapping of Complex Trait Associated Loci in the Human Liver
Source: Am J Hum Genet. 2019 Jun 13;105(1):89–107. doi: 10.1016/j.ajhg.2019.05.010 (PMC6612522; doi:10.1016/j.ajhg.2019.05.010)
Supplement: Document S1. Figures S1–S16 [file mmc1.pdf]

The American Journal of Human Genetics, Volume *105*

Supplemental Data

Genetic and Epigenetic Fine Mapping of Complex Trait Associated Loci in the Human Liver

Minal Çalışkan, Elisabetta Manduchi, H. Shanker Rao, Julian A. Segert, Marcia Holsbach Beltrame, Marco Trizzino, YoSon Park, Samuel W. Baker, Alessandra Chesi, Matthew E. Johnson, Kenyaita M. Hodge, Michelle E. Leonard, Baoli Loza, Dong Xin, Andrea M. Berrido, Nicholas J. Hand, Robert C. Bauer, Andrew D. Wells, Kim M. Olthoff, Abraham Shaked, Daniel J. Rader, Struan F.A. Grant, and Christopher D. Brown

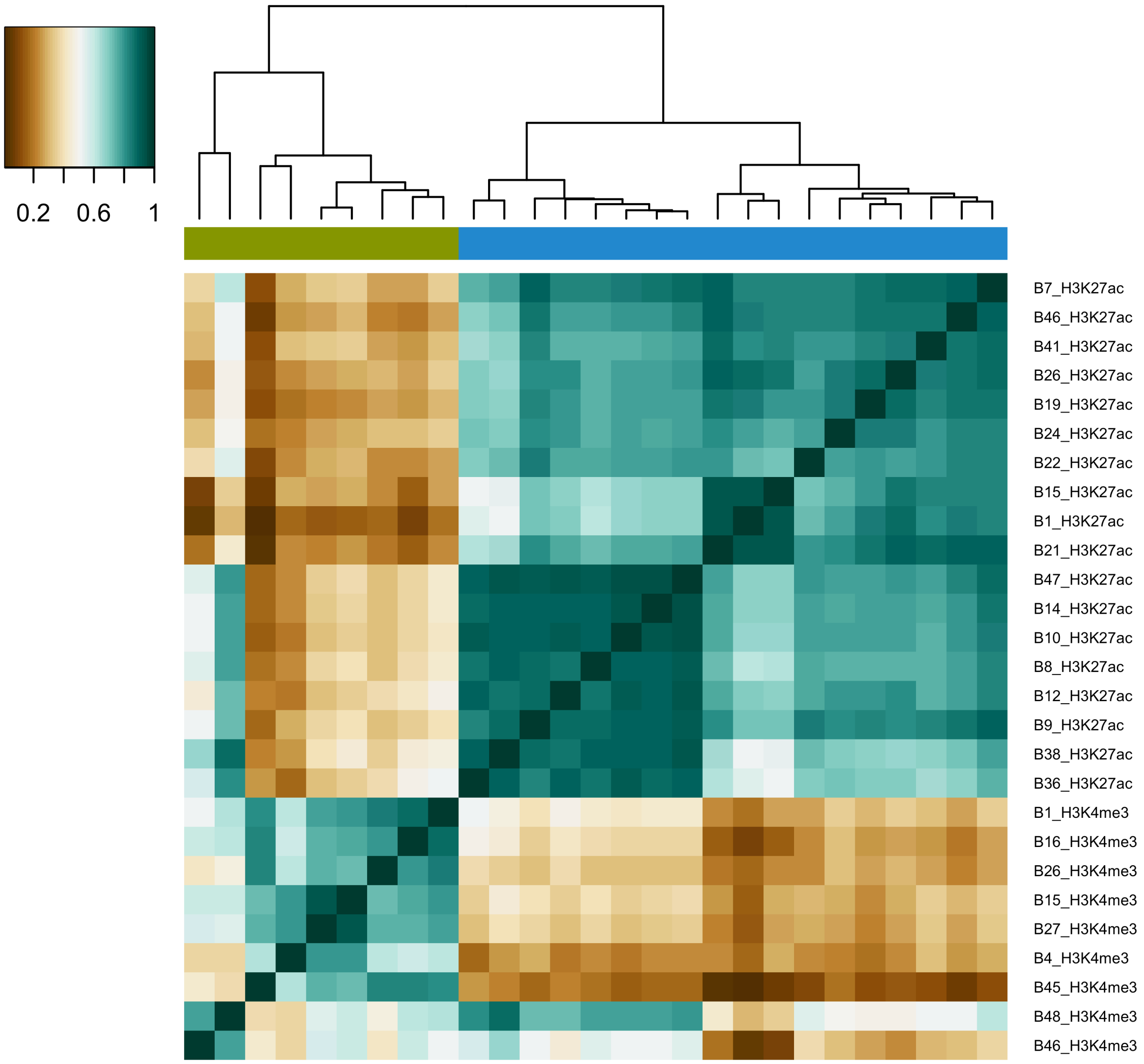

Figure S1. Heatmap plot of Spearman's correlation of ChIP-Seq data. Color bars above the heatmap plot indicate the ChIP-seq data type; columns corresponding to H3K4me3 data are shown in green, columns corresponding to H3K27ac data are shown in blue. Subject IDs are included as row labels.

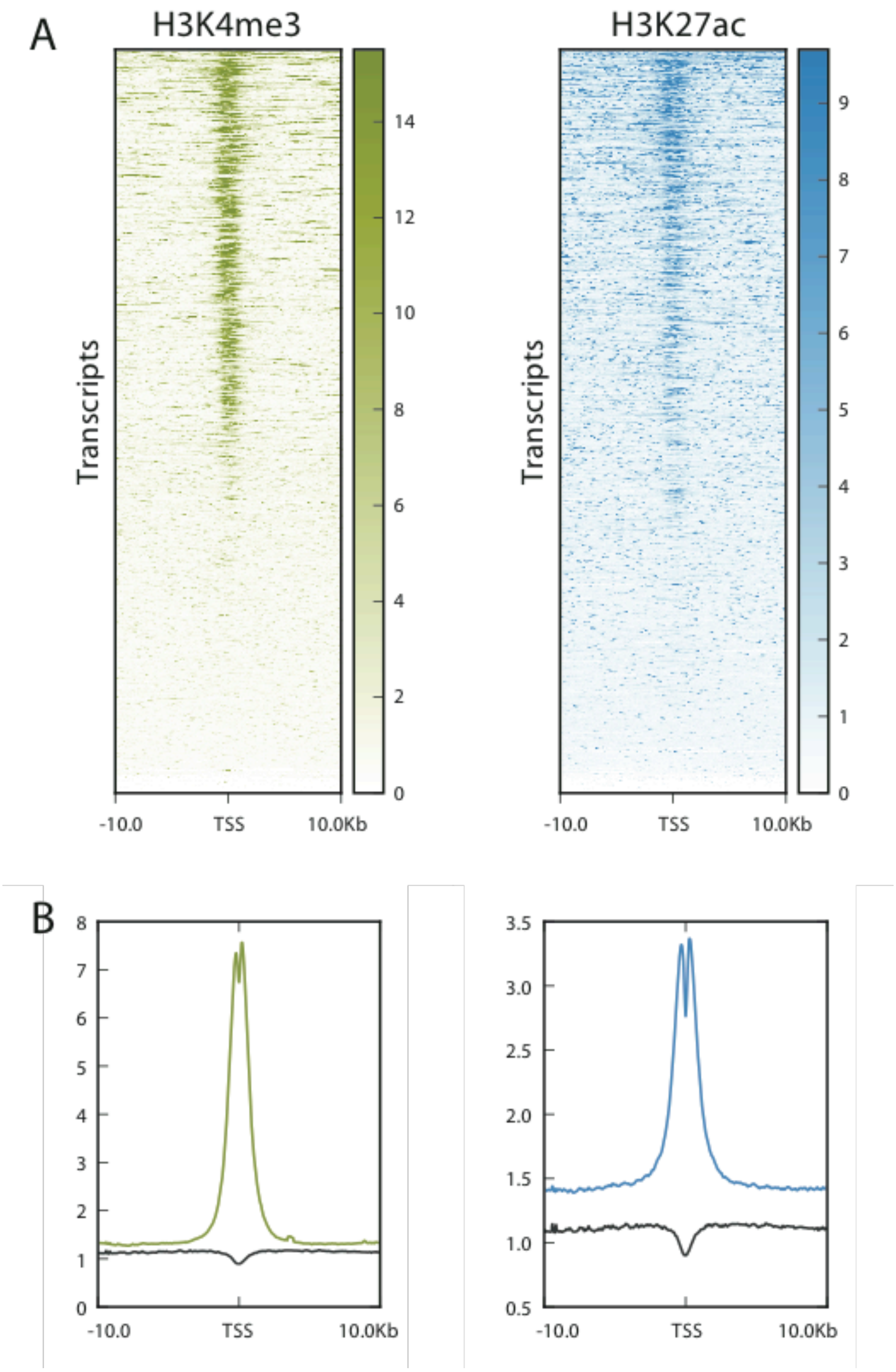

Figure S2. A) Heatmap plots of ChIP-Seq read counts in 20 kb regions centered on GENCODE v19 transcript TSS positions **B)** Profile plots of ChIP and Input sequencing read counts centered on GENCODE v19 transcript TSS positions. Green line corresponds to H3K4me3, blue line corresponds to H3K27ac, and black lines correspond to their Input DNA data.

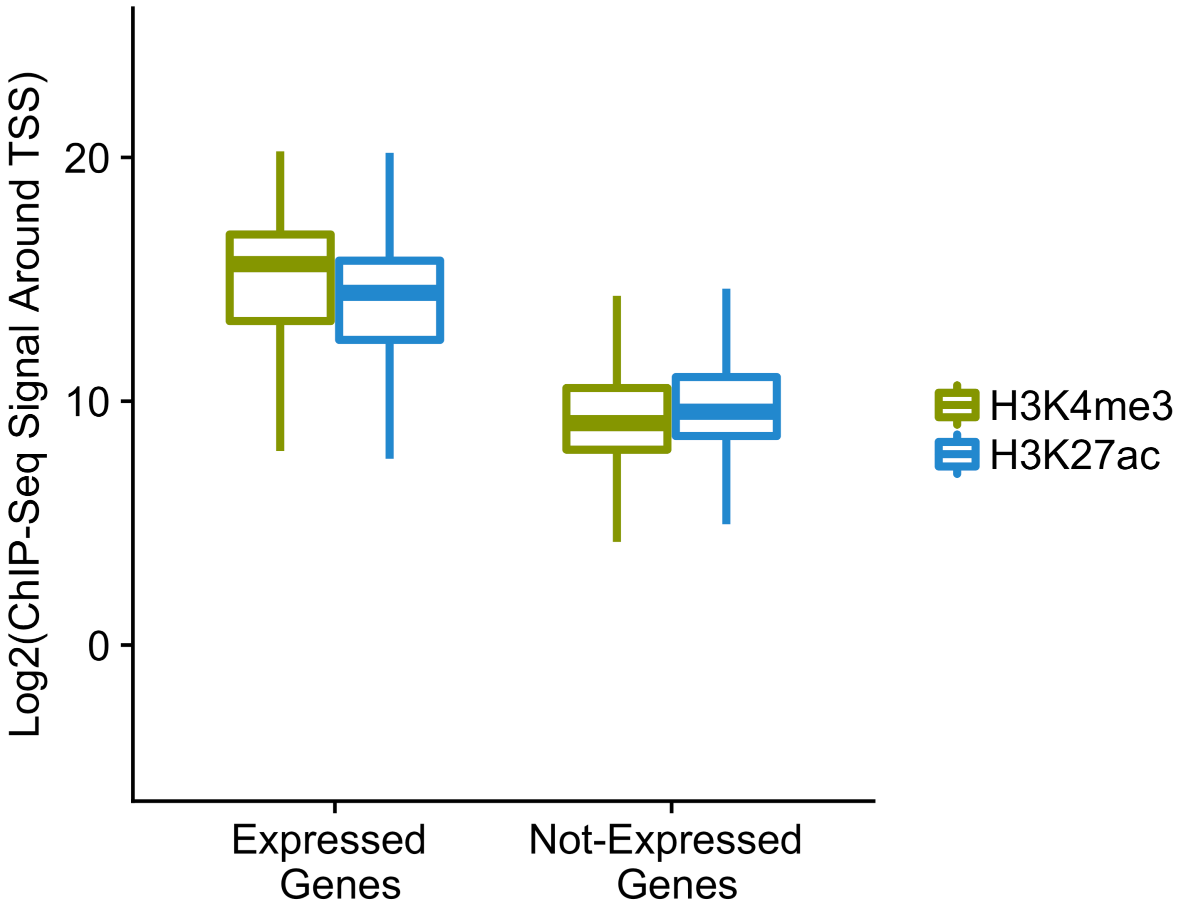

Figure S3. H3K4me3 and H3K27ac ChIP-Seq read counts in 2 kb regions centered on GENCODE v19 transcript TSS positions were extracted from merged ChIP-Seq data of biological replicates (N=9 for H3K4me3 and N=18 for H3K27ac). ChIP-Seq read counts around TSS of the 19,133 expressed genes were significantly higher than that of 38,687 genes that were not detected as expressed in Penn Cohort 1.

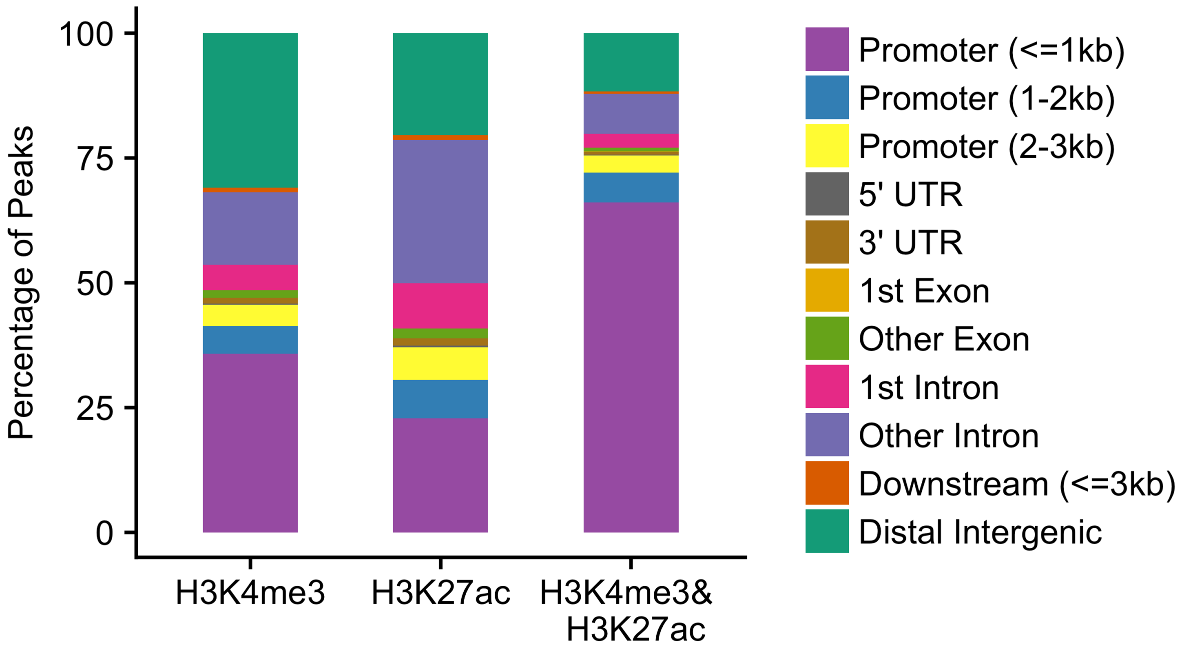

Figure S4. Genomic annotations of the ChIP-Seq peaks. Annotations are displayed for 68,600 H3K4me3 peaks, 131,293 H3K27ac peaks and 28,167 overlapping peaks between H3K4me3 and H3K27ac datasets.

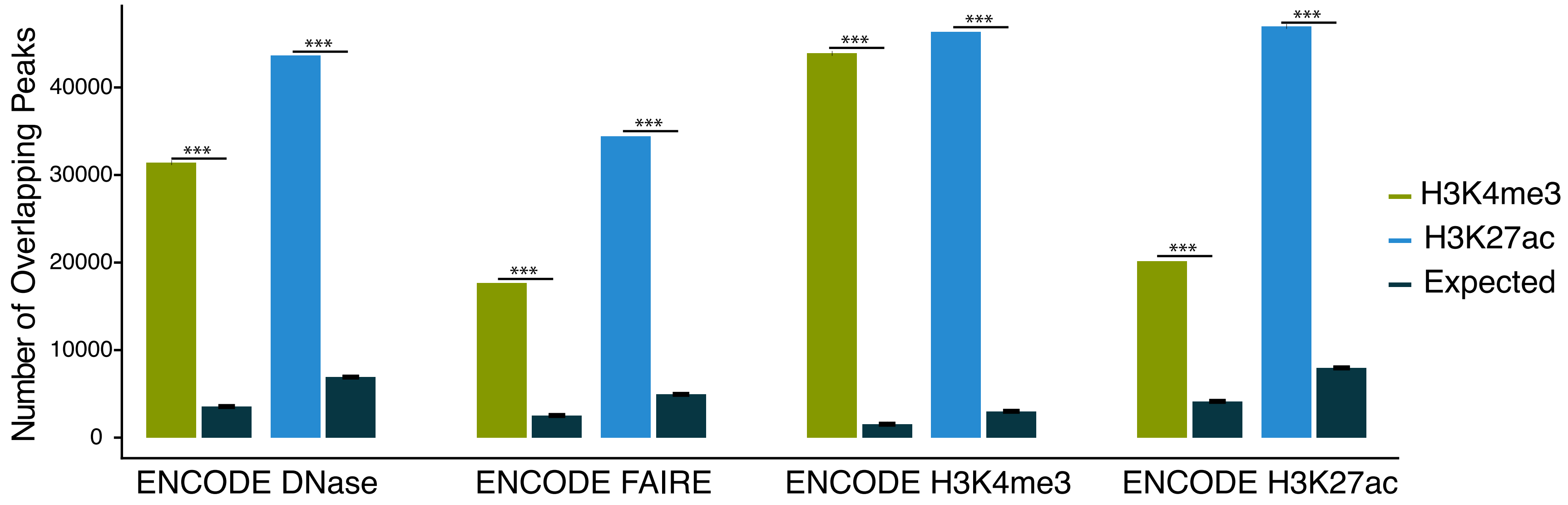

Figure S5. Observed and expected numbers of H3K4me3 and H3K27ac peaks overlapping ENCODE datasets in HepG2 cells. Expected numbers represent the mean overlap based on 1,000 sets of randomly selected size-matching regions for each of the original peaks. Error bars represent standard error of the mean. One-tailed Fisher's exact test P-values were $<2.2 \times 10^{-16}$ for all comparisons.

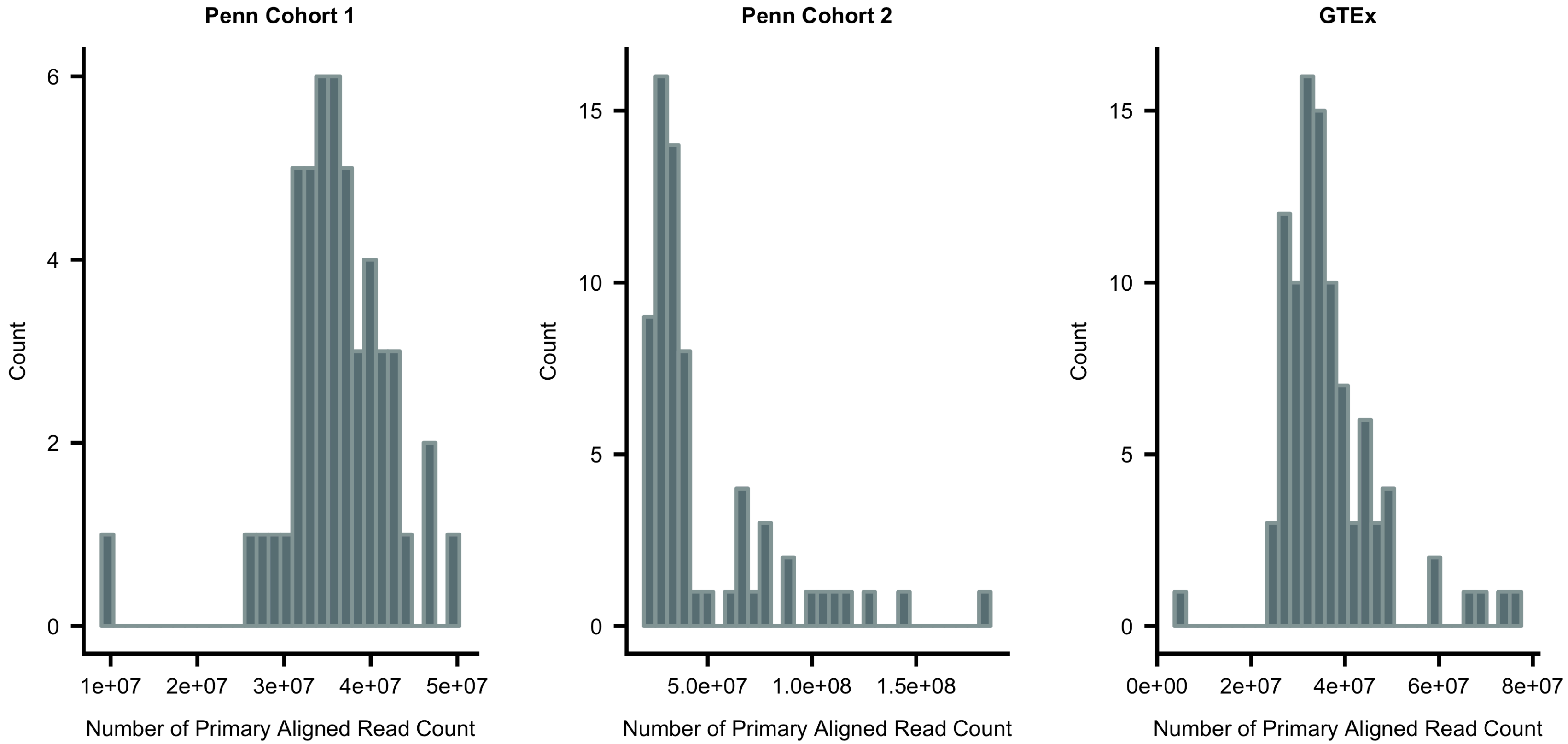

Figure S6. Histogram of the number of primary aligned RNA-seq reads to the reference human genome (hg19). Median read counts were 35.86, 32.72, 33.74 million for Penn Cohort 1, Penn Cohort 2, and GTEx, respectively.

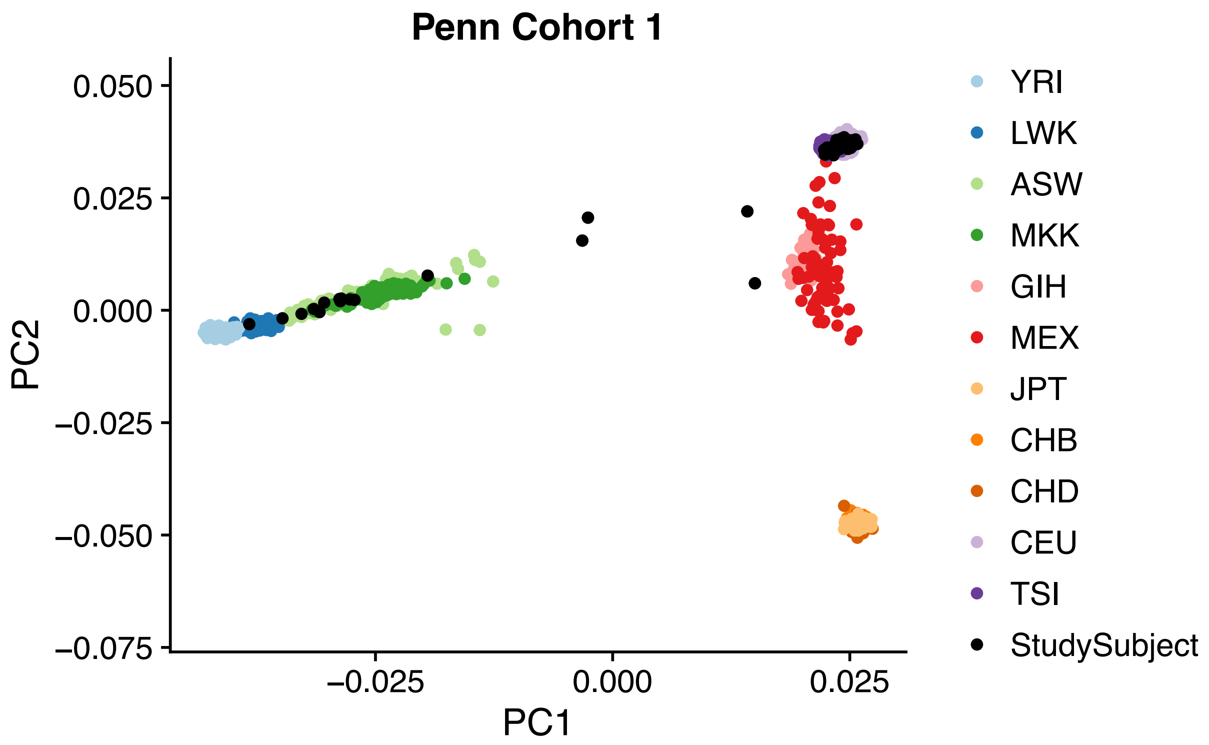

Figure S7. PCA plot of the genotype data of Penn Cohort 1 individuals. Plot displays the first two principal components of LD pruned genotype data from 50 study subjects (shown in black) with 1,184 HapMap Phase 3 individuals representing 11 populations. 34 of 50 individuals clustered with the HapMap European populations, 12 of them clustered with the HapMap African populations, and the remaining 4 individuals displayed mixed genetic ancestry.

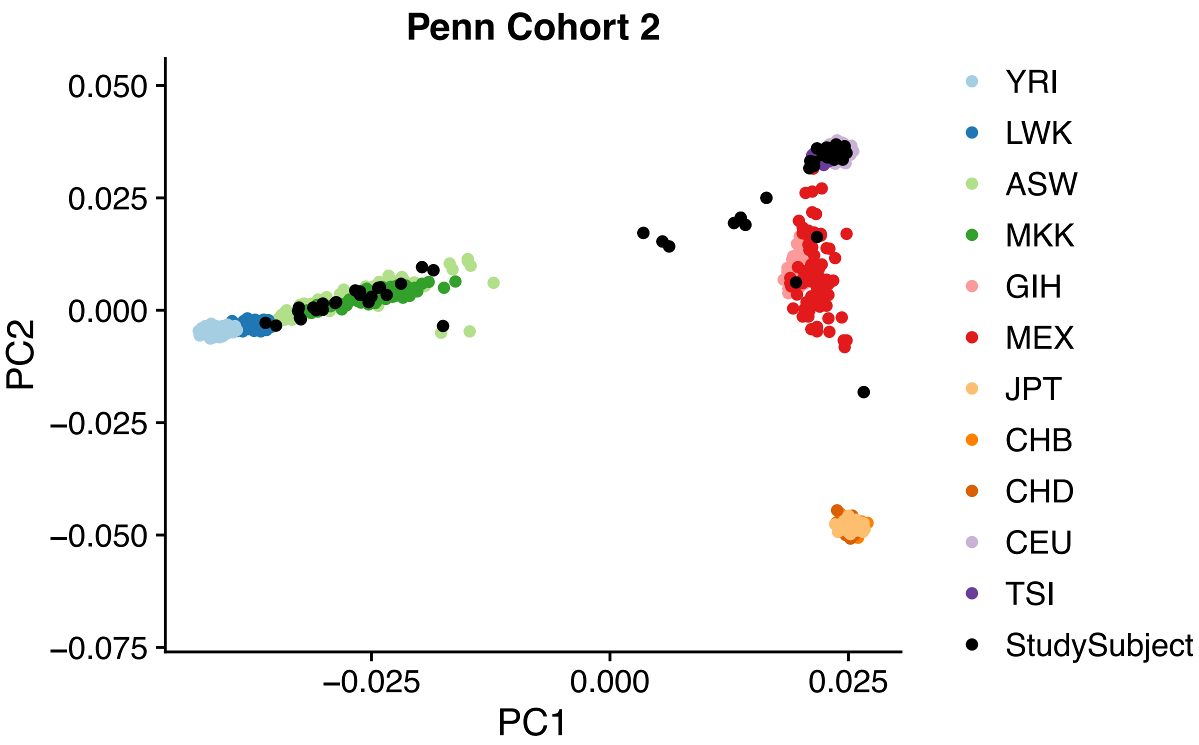

Figure S8. PCA plot of the genotype data of Penn Cohort 2 individuals. Plot displays the first two principal components of LD pruned genotype data from 96 study subjects (shown in black) with 1,184 HapMap Phase 3 individuals representing 11 populations. 62 of 96 individuals clustered with the HapMap European populations, 24 of them clustered with the HapMap African populations, and the remaining 10 individuals displayed mixed genetic ancestry.

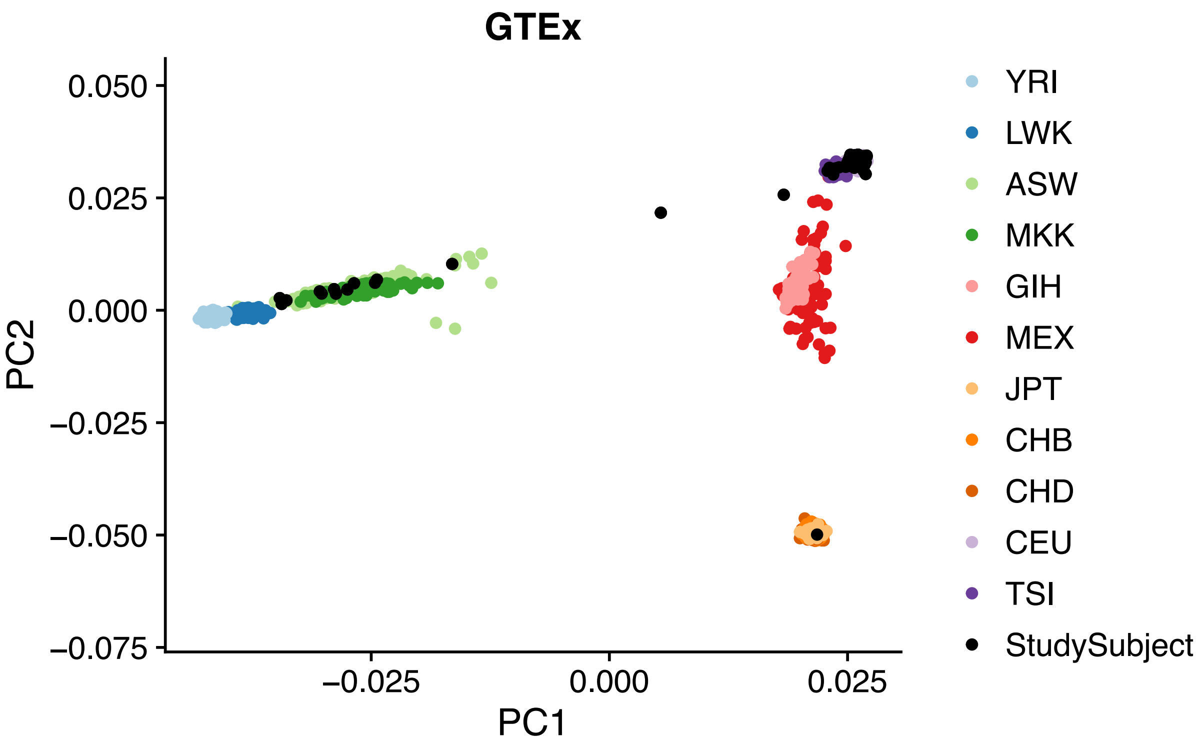

Figure S9. PCA plot of the genotype data of GTEx individuals. Plot displays the first two principal components of LD pruned genotype data from 96 study subjects (shown in black) with 1,184 HapMap Phase 3 individuals representing 11 populations. 81 of 96 individuals clustered with the HapMap European populations, 12 of them clustered with the HapMap African populations, one individual clustered with the HapMap Asian populations and the remaining two individuals displayed mixed genetic ancestry.

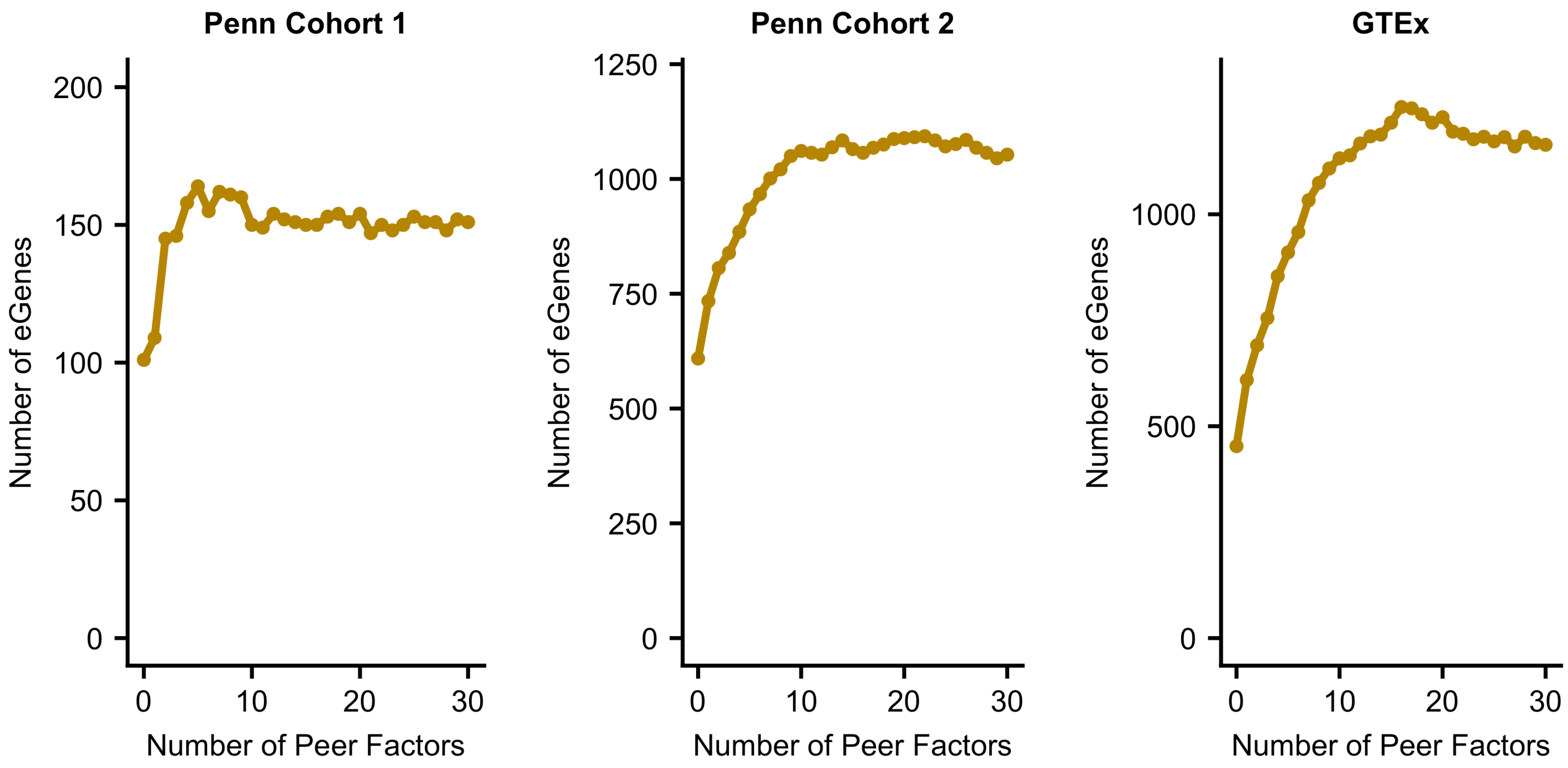

Figure S10. Number of eQTL-Genes identified as a function of number of Peer factor covariates that were included in cis-eQTL mapping (based on FastQTL). Number of eQTL-Genes were maximized when 5, 22, and 16 Peer Factors were used for Penn Cohort 1, Penn Cohort 2, and GTEx, respectively.

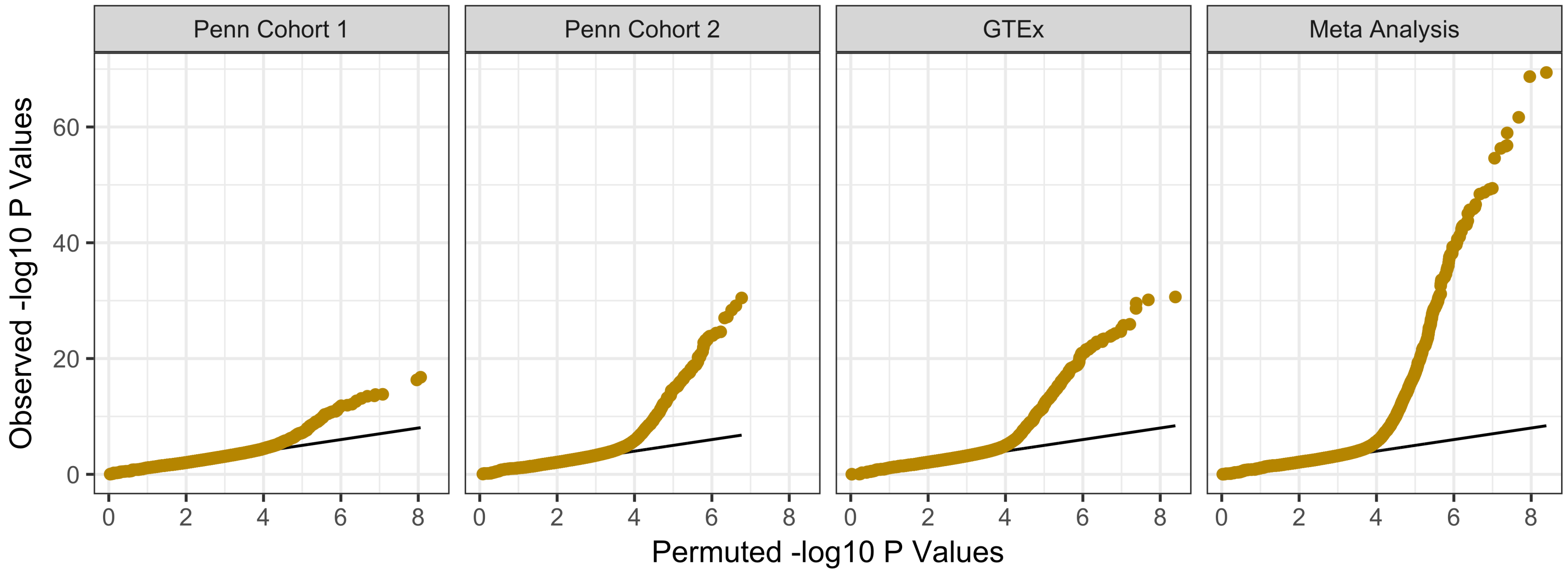

Figure S11. QQ-plots of the cis-eQTL association P-values within each cohort (Panels 1 to 3) and meta P-values across cohorts (Panel 4). Solid lines represent the distribution of P-values based on permuted data. A total of 2,625 cis eQTL-Genes were identified based on meta-analysis eQTL results. See Figure 1 for numbers of significant results within each cohort.

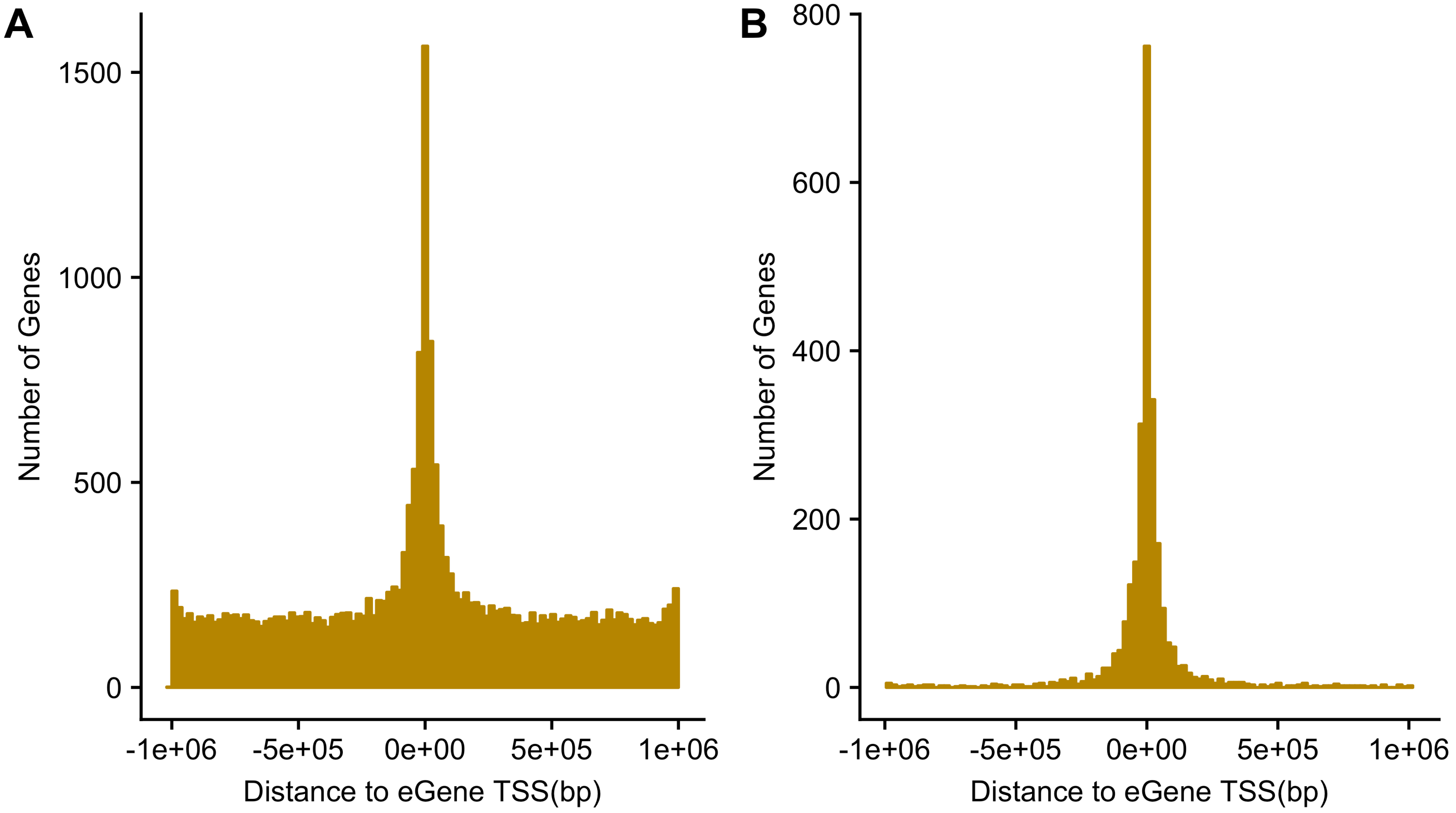

Figure S12. Lead SNP – target gene distance based on **A)** All genes **B)** Genes with significant meta cis-eQTLs

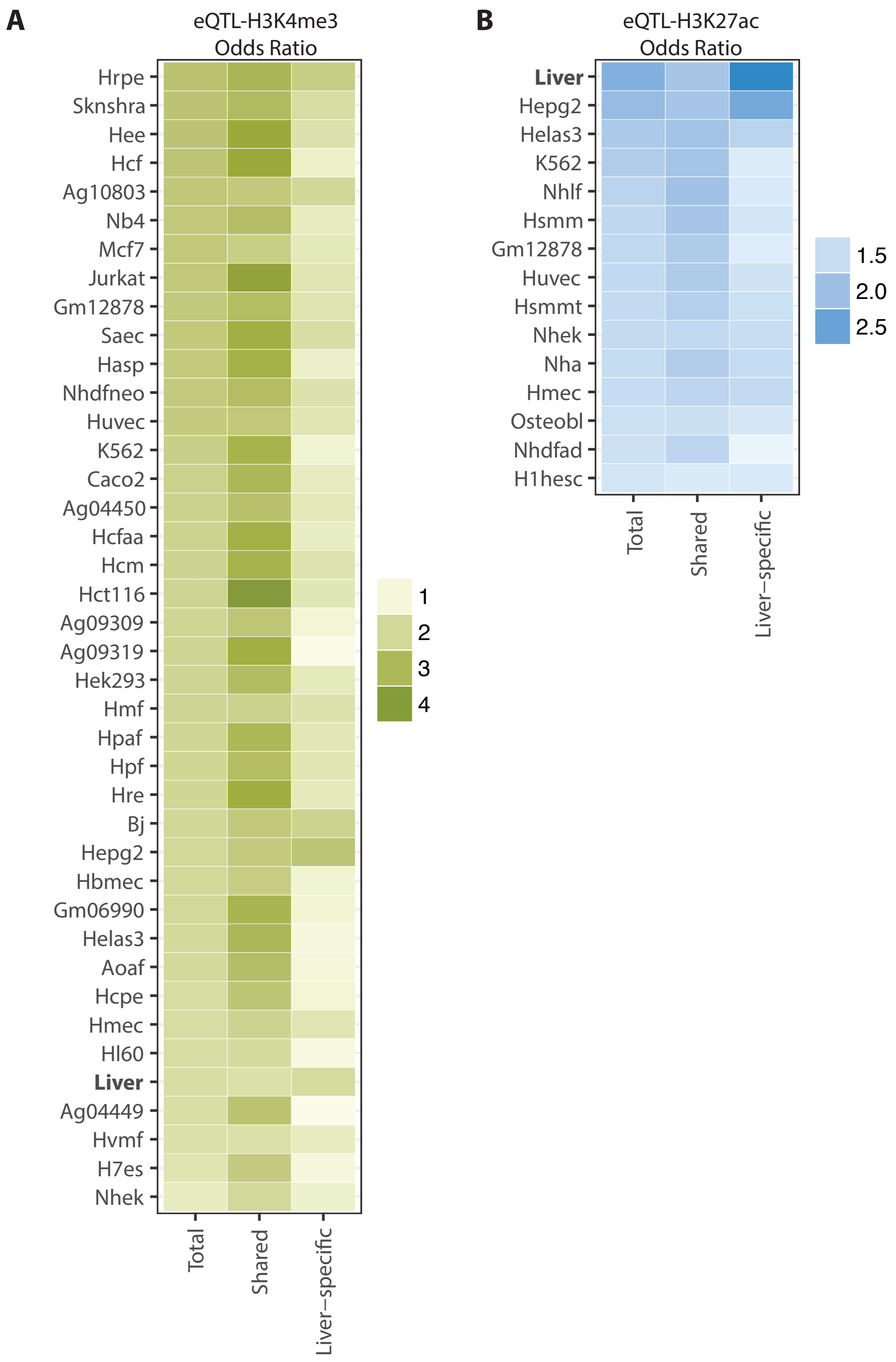

Figure S13. A) Odds ratios of overlap between H3K4me3 datasets and total, shared, and liver-specific cis-eQTLs **B)** Odds ratios of overlap between H3K27ac datasets and total, shared, and liver specific cis-eQTLs.

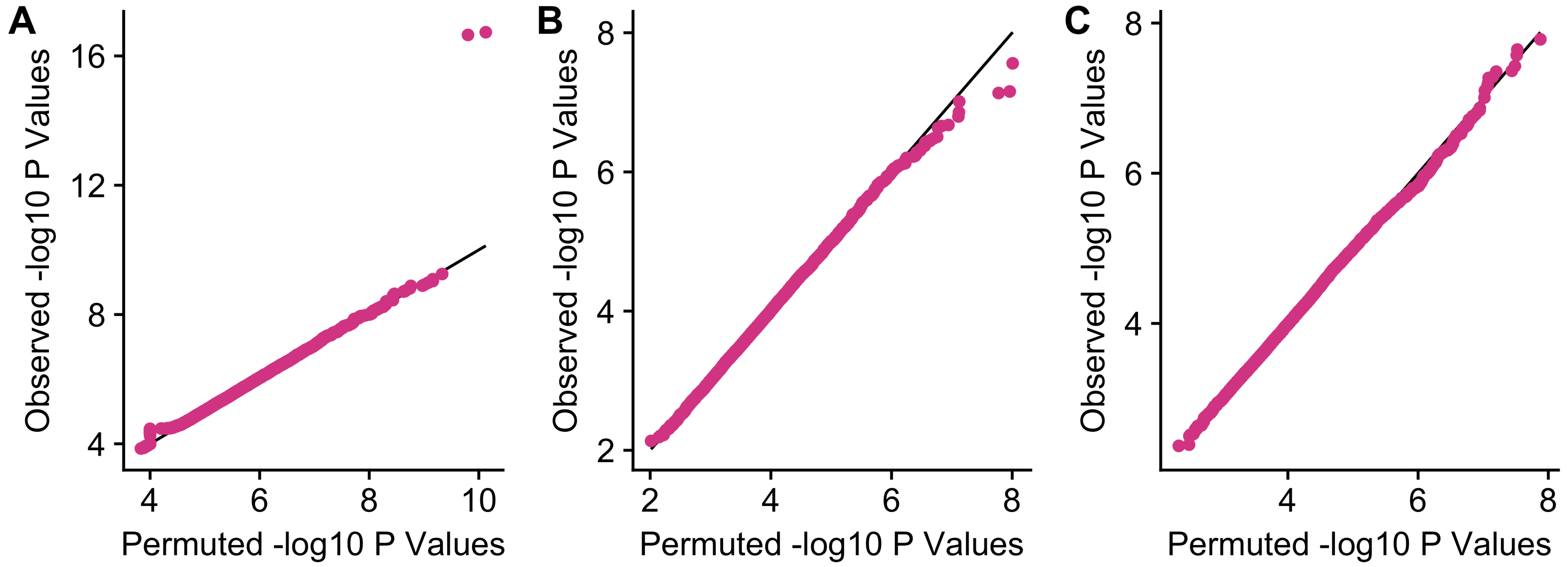

Figure S14. QQ-plots of the meta trans-eQTL association P-values using **A)** all linkage disequilibrium pruned variants in the genome **B)** cis-eQTL variants **C)** variants likely to affect activity of transcription factors. Magenta points correspond to observed P-values. Solid lines represent the expected distribution of P-values based on permuted gene expression data.

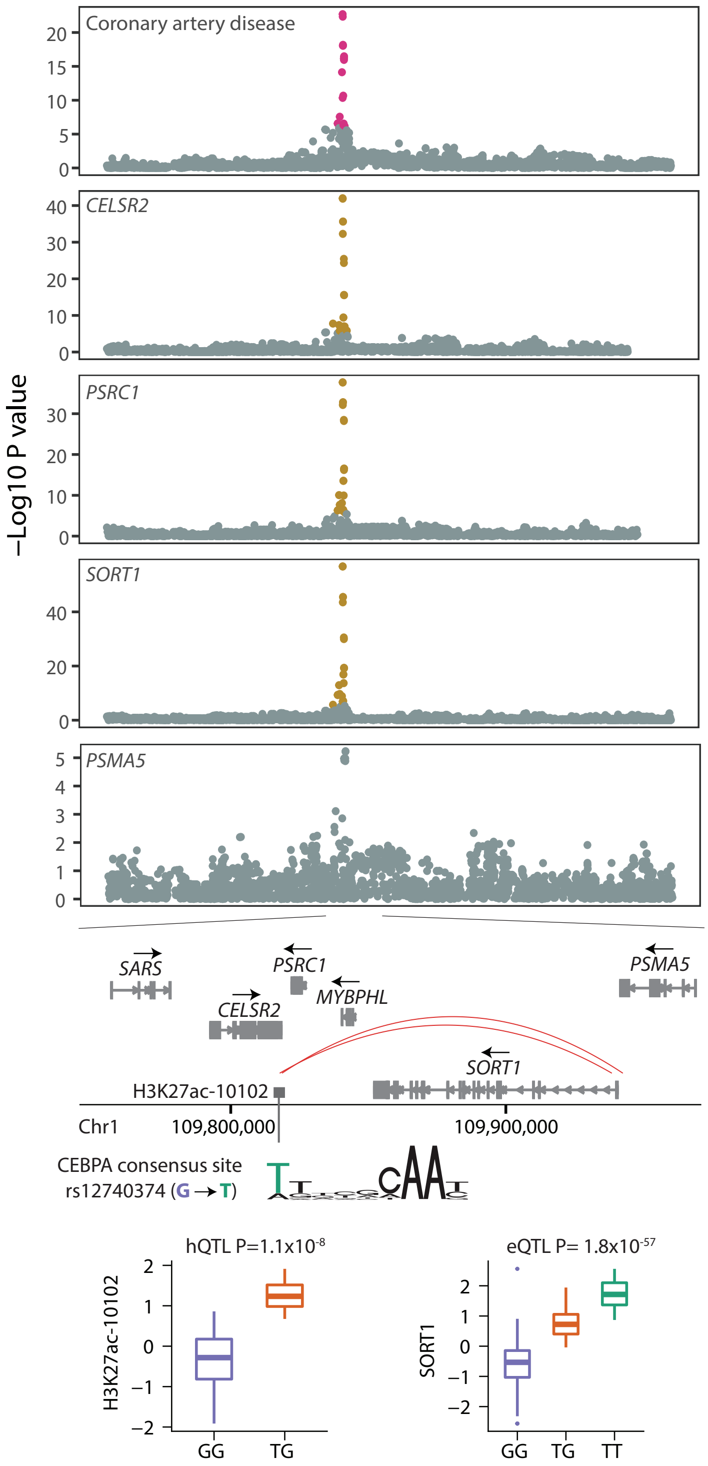

Figure S15. Significant colocalization signals at the chromosome 1p13.3 locus. Colocalization posterior probabilities of coronary artery disease associations with *CELSR2*, *PSRC1*, *SORT1* and *PSMA5* gene expression levels were 0.999, 0.999, 0.999, and 0.947,

respectively. Schematic representation of the genes in the zoomed-in locus of chr1: 109,750,000-109,940,573 and the putatively causal H3K27ac-10102 peak (chr1: 109,816,977-109,818,871). H3K27ac-10102 peak only formed DNA looping interaction with the promoter of the *SORT1* gene in the genome (CHiCAGO scores: 6.63 and 7.8). The T allele of the candidate causal variant, rs12740374, increases the odds of CEBPA binding. Box plots of normalized H3K27ac-10102 ChIP-Seq and *SORT1* RNA-Seq read counts are stratified by genotype at the rs12740374. Sample sizes of each genotype group were GG:14, TG:4 for ChIP-Seq data and GG:143, TG:83, TT:14 for RNA-Seq data.

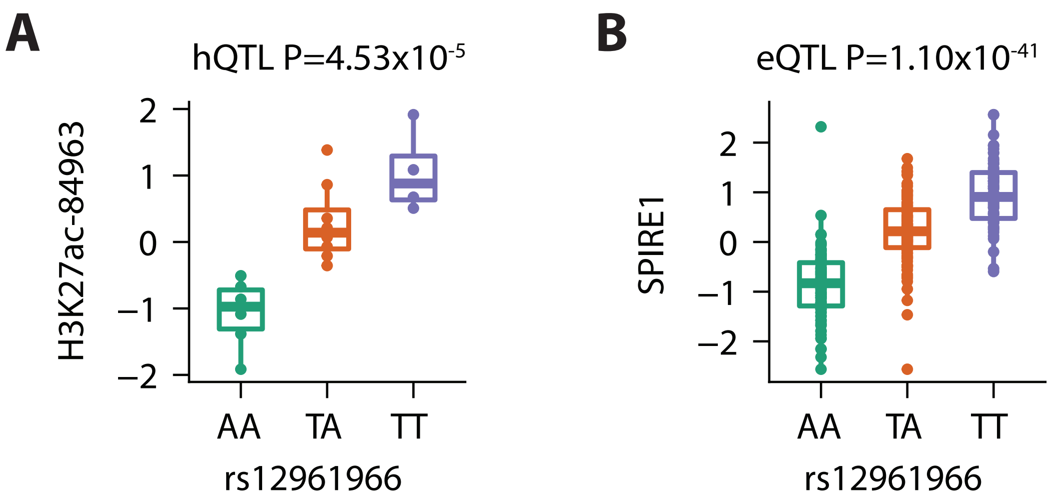

Figure S16. An example of putatively co-regulated histone modification and gene expression levels. Box plots of normalized H3K27ac-84963 ChIP-Seq and *SPIRE1* RNA-Seq read counts are stratified by genotype at the rs12961966. Sample sizes of each genotype group were TT:4, AT:8, AA:5 for ChIP-Seq data and TT:55, AT:94, AA:88 for RNA-Seq data.
