# Supplementary material for: Genetic and Epigenetic Fine Mapping of Complex Trait Associated Loci in the Human Liver
Source: Am J Hum Genet. 2019 Jun 13;105(1):89–107. doi: 10.1016/j.ajhg.2019.05.010 (PMC6612522; doi:10.1016/j.ajhg.2019.05.010)
Supplement: Document S2. Article plus Supplemental Data [file mmc3.pdf]

Genetic and Epigenetic Fine Mapping of Complex Trait Associated Loci in the Human Liver

Minal Çalışkan,[1,2,*] Elisabetta Manduchi,[3,4,5] H. Shanker Rao,[1,2] Julian A. Segert,[1] Marcia Holsbach Beltrame,[1] Marco Trizzino,[1] YoSon Park,[1] Samuel W. Baker,[1] Alessandra Chesi,[3,4] Matthew E. Johnson,[3,4] Kenyaita M. Hodge,[3,4] Michelle E. Leonard,[3,4] Baoli Loza,[6] Dong Xin,[6] Andrea M. Berrido,[1] Nicholas J. Hand,[1] Robert C. Bauer,[7] Andrew D. Wells,[4,8] Kim M. Olthoff,[6] Abraham Shaked,[6] Daniel J. Rader,[1,9] Struan F.A. Grant,[1,3,4,10] and Christopher D. Brown[1,2,*]

Deciphering the impact of genetic variation on gene regulation is fundamental to understanding common, complex human diseases. Although histone modifications are important markers of gene regulatory elements of the genome, any specific histone modification has not been assayed in more than a few individuals in the human liver. As a result, the effects of genetic variation on histone modification states in the liver are poorly understood. Here, we generate the most comprehensive genome-wide dataset of two epigenetic marks, H3K4me3 and H3K27ac, and annotate thousands of putative regulatory elements in the human liver. We integrate these findings with genome-wide gene expression data collected from the same human liver tissues and high-resolution promoter-focused chromatin interaction maps collected from human liver-derived HepG2 cells. We demonstrate widespread functional consequences of natural genetic variation on putative regulatory element activity and gene expression levels. Leveraging these extensive datasets, we fine-map a total of 74 GWAS loci that have been associated with at least one complex phenotype. Our results reveal a repertoire of genes and regulatory mechanisms governing complex disease development and further the basic understanding of genetic and epigenetic regulation of gene expression in the human liver tissue.

Introduction

The liver has a central role in detoxification of endogenous and exogenous toxins, synthesis of essential proteins, and regulation of carbohydrate, lipid, and drug metabolism. As such, the liver is associated with a diverse range of clinically important human traits[1] and was recently reported as one of the most critical tissues for explaining cellular mechanisms at loci revealed by genome-wide association studies (GWASs).[2] GWASs have been effective at providing robust, but imprecise, information about genetic risk factors of complex human diseases.[3] These studies have revealed that most variation associated with complex human phenotypes do not alter protein-coding sequences, making causal variant and trait-relevant gene identification a considerable challenge.[4] Characterization of the regulatory functions of non-coding regions is the first key step toward linking non-coding regions to disease biology. Large-scale efforts such as the Encyclopedia of DNA Elements (ENCODE)[5] and the NIH Roadmap Epigenomics[6] consortia have made major contributions to this end. It remains an important priority to obtain such data across many individuals, to characterize the extent of between-individual variation in the activity of regulatory elements, and to identify the genetic determinants of such differential activity. Discovering genotype-dependent non-coding functional activity can help to fine map and reveal mechanisms underlying complex trait associations.[7–14] Performing such studies at genome-wide scale in large numbers of human tissues is challenging[15] and therefore has been limited to those performed in easily accessible lymphoblastoid cell lines or blood cell types.[16–22] Here, we quantify regulatory element activity in the human liver across multiple individuals and integrate these findings with genome-wide gene expression data collected from the same human liver tissues, high-resolution promoter-focused chromatin interaction maps collected from human liver-derived HepG2 cells, and GWAS summary statistics for 20 commonly studied phenotypes with variable levels of suggested causality manifesting in the liver.[2] We identify 2,625 genes and 972 regulatory elements with genotype-dependent activity in the human liver and fine-map a total of 74 GWAS loci that have been associated with at least one complex phenotype. Overall, we provide a unique resource that contributes to basic understanding of genetic and epigenetic regulation of gene expression in the human liver tissue and highlight the benefits of integrating multiple cellular traits for the identification and characterization of disease-relevant genes, regulatory elements, and variants.

[1]Department of Genetics, Perelman School of Medicine, University of Pennsylvania, Philadelphia, PA 19104, USA; [2]Institute for Biomedical Informatics, Perelman School of Medicine, University of Pennsylvania, Philadelphia, PA 19104, USA; [3]Division of Human Genetics, The Children's Hospital of Philadelphia, Philadelphia, PA 19104, USA; [4]Center for Spatial and Functional Genomics, The Children's Hospital of Philadelphia, Philadelphia, PA 19104, USA; [5]Department of Biostatistics, Epidemiology, & Informatics, Perelman School of Medicine, University of Pennsylvania, Philadelphia, PA 19104, USA; [6]Division of Transplant Surgery, Perelman School of Medicine, University of Pennsylvania, Philadelphia, PA 19104, USA; [7]Division of Cardiology, Columbia University, New York, NY 10032, USA; [8]Department of Pathology and Laboratory Medicine, University of Pennsylvania Perelman School of Medicine, Philadelphia, PA 19104, USA; [9]Department of Medicine, Perelman School of Medicine, University of Pennsylvania, Philadelphia, PA 19104, USA; [10]Department of Pediatrics, Perelman School of Medicine, University of Pennsylvania, Philadelphia, PA 19104, USA
*Correspondence: minal@mail.med.upenn.edu (M.Ç.), chrbro@pennmedicine.upenn.edu (C.D.B.)
https://doi.org/10.1016/j.ajhg.2019.05.010.
© 2019 The Author(s). This is an open access article under the CC BY license (http://creativecommons.org/licenses/by/4.0/).

Material and Methods

Study Subjects

Penn Cohort 1

Samples in this cohort were prospectively collected between August 2014 and February 2015 at the Penn Transplant Institute. The cohort was comprised of 50 liver donors (49 deceased, 1 living). Sex and age of the donors were reported as 19 females and 31 males aged between 6 and 77 years old.

Penn Cohort 2

Between January 2012 and August 2014, ~25 mg of liver needle biopsy samples were collected from deceased donors prior to transplantation surgery at the Penn Transplant Institute. All samples were stored in RNAlater. For this study, 96 samples were chosen based on cold ischemic time (138–320 min) and reported sex (48 females, 48 males). Age of the donors ranged between 7 and 75 years old.

GTEx Cohort

Complete description of the Genotype-Tissue expression (GTEx) cohort was published previously.[23,24] In this study, 96 individuals (33 females, 63 males; age range 21–68) with genotype and liver gene expression data were included. 37 of the subjects were organ donors and 59 were postmortem. Liver needle biopsy samples from each subject were obtained in two centers. Samples were preserved in PAXgene tissue kits and shipped to the GTEx Laboratory Data Analysis and Coordinating Center LDACC at the Broad Institute for processing.[23,24] All GTEx datasets used in this study were from GTEx Analysis Releasev6p.[25]

ChIP-Seq Experimental Protocol

Penn Cohort 1

Between 40 and 900 mg of liver wedge biopsies were obtained from each donor prior to transplantation surgery at the Penn Transplant Institute. Flash frozen liver wedge biopsies were processed in a total of 8 batches (six/eight randomized samples per day). On each tissue preparation day, 20 mg of tissue from each liver sample was cut, placed in 1 mL of RNAlater, and flash frozen for isolation of DNA and RNA at a later date. When available, 120 mg of tissue from each subject was processed for the chromatin immunoprecipitation (ChIP) experiment. From 33 subjects, 120 mg of tissue could be used. Tissue amount from the remaining 17 subjects was limited, so the largest amount available was used (ranging between 20 and 110 mg). The tissue was cut into small pieces (~1 mm^3), washed with PBS, and fixed with 1% formaldehyde for 5 min at room temperature. Nuclei were prepared with the Covaris truChIP Tissue Chromatin Shearing Kit with SDS Shearing Buffer according to manufacturer's recommendations. Chromatin was sheared for 14 min at 5% duty cycle, 140 Watts peak incident power, and 200 cycles per burst using a Covaris S220 Focused-ultrasonicator. Shearing efficiency was assessed using the Agilent High Sensitivity DNA kit and chromatin concentration was determined using a NanoDrop Spectrophotometer. From each subject, a 0.5 μg aliquot of sheared chromatin was kept aside to be used as input chromatin. Each immunoprecipitation was performed using 9 μg of sheared chromatin and 5 μg of antibody (H3K27ac:ab4729, H3K4me3:ab8580) with an overnight incubation at 4°C following the Magna ChIP A/G Chromatin Immunoprecipitation Kit protocol. After elution and reverse-cross-linking of protein-DNA complexes, DNA was cleaned with a QIAGEN QIAquick PCR Purification Kit and quantified using the Agilent High Sensitivity DNA kit. 40 H3K27ac and 45 H3K4me3 samples yielded sufficient DNA (≥2 ng) to generate sequencing libraries. 2 or 5 ng of immunoprecipitated and input DNA was used to generate sequencing libraries using the NEBNext ChIP-Seq Library Prep Master Mix Set for Illumina. Libraries were sequenced to generate 100 bp single-end reads on Illumina HiSeq2500 instruments at the Penn Next-Generation Sequencing Core.

RNA-Seq and Genotyping Experimental Protocol

Penn Cohort 1

RNA and DNA were extracted in a total of 4 batches (12 or 14 randomized samples per day) using QIAGEN's AllPrep DNA/RNA/miRNA Universal Kit. Barcoded, strand-specific, polyA+ selected RNA-seq libraries were generated using the Illumina TruSeq Stranded mRNA kit. Quality of each library was assessed using the Agilent Bioanalyzer High Sensitivity DNA Kit. Libraries were then pooled into one group and sequenced to generate 125 bp paired-end reads on Illumina HiSeq2500 instruments at the Penn Next-Generation Sequencing Core. DNA was genotyped using Illumina HumanCoreExome arrays at the Center for Applied Genomics Core at the Children's Hospital of Pennsylvania.

Penn Cohort 2

RNA and DNA extraction method and library preparation was identical to that of Penn Cohort 1. Libraries were pooled into two groups of 48 samples and sequenced to generate 125 bp paired-end reads on Illumina HiSeq2500 instruments at the Penn Next-Generation Sequencing Core. DNA was genotyped using Illumina HumanCoreExome arrays at the Center for Applied Genomics Core at at the Children's Hospital of Pennsylvania.

GTEx

RNA was extracted from 96 human liver samples as described previously.[26] Non-strand specific, polyA+ selected RNA-seq libraries were generated using the Illumina TruSeq protocol. Libraries were sequenced to generate 76 bp paired end reads. DNA was extracted from whole blood using the QIAGEN Gentra Puregene method and genotyped using the Illumina Human Omni 2.5M and 5M-Quad BeadChip as described previously.[26]

Genome-wide Promoter-Focused Capture-C Experimental Protocol

Cell Fixation for Chromatin Capture

The protocol used for cell fixation was similar to previously published methods.[27] HepG2 cells were collected and single-cell suspension was made with aliquots of 10^7 cells in 10 mL media (RPMI + 10% FCS). 540 μL 37% formaldehyde was added and incubation was carried out for 10 min at room temperature in a tumbler. The reaction was quenched by adding 1.5 mL, 1 M cold glycine (4°C). Fixed cells were centrifuged for 5 min at 1,000 × g at 4°C, and supernatant was removed. The pellets were washed in 10 mL cold PBS (4°C) by centrifugation for 5 min at 1,000 × g at 4°C. Supernatant was removed and cell pellets were resuspended in 5 mL of cold lysis buffer (10 mM Tris [pH 8], 10 mM NaCl, 0.2% NP-40 [Igepal] supplemented with protease inhibitor cocktails). Resuspended cells were incubated for 20 min on ice and centrifuged to remove the lysis buffer. Finally, the pellets were resuspended in 1 mL lysis buffer and transferred to 1.5 mL Eppendorf tubes prior to snap freezing (ethanol/dry ice or liquid nitrogen). Cells were stored at −80°C until they were thawed again for digestion.

3C Library Generation

For preparation of initial 3C libraries, 10 million cells were harvested and fixed. Cells were thawed on ice and spun down, and

the lysis buffer was removed. The pellet was resuspended in water and incubated on ice for 10 min, followed by centrifugation and removal of supernatant. The pellet was then resuspended with 20% SDS and 1 × NEBuffer DpnII and incubated at 37°C for 1 h at 1,000 rpm on a MultiTherm (Sigma-Aldrich). Triton X-100 (at 20% concentration) was added and the pellet was incubated for another 1 h. After the incubation, 10 μL 50 U/μL DpnII (NEB) was added and left to digest for 8 h. An additional 10 μL DpnII was added and digestion was left overnight at 37°C. The next day, another 10 μL of DpnII was added and incubated for an additional 3 h. The chromatin was then ligated overnight (8 μL T4 DNA Ligase, HC ThermoFisher [30 U/μL]; with final concentration, 10 U/mL) and shaken at 16°C at 1,000 rpm on the MultiTherm. The next day, an additional 2 μL T4 DNA ligase was spiked in to each sample and incubated for 3 more hours. The ligated samples were de-crosslinked overnight at 65°C with Proteinase K (Invitrogen) and the following morning incubated for 30 min at 37°C with RNase A (Millipore). Phenol-chloroform extraction was performed, followed by an ethanol precipitation overnight at −20°C and then washed with 70% ethanol. Digestion efficiencies of 3C libraries were assessed by gel electrophoresis on a 0.9% agarose gel and quantitative PCR (SYBR green, Thermo Fisher).

Capture-C

Custom capture baits were designed using Agilent SureSelect library design targeting both ends of DpnII restriction fragments encompassing promoters (including alternative promoters) of all human coding genes and non-coding RNAs (antisense RNA, snRNA, miRNA, snoRNA, and lincRNA), totaling 36,691 RNA baited fragments. The capture library design successfully covered 95% of the coding gene promoters and 88% of the non-coding RNA promoters. Custom capture bait design failed for 5% of the coding genes, which were either duplicated genes or contained highly repetitive DNA in their promoter regions. The isolated DNA of the 3C libraries generated by DpnII digestion and ligation was quantified using a Qubit fluorometer (Life Technologies), and 10 μg of each library was sheared in dH_2O using a QSonica Q800R to an average DNA fragment size of 350 bp. QSonica settings used were 60% amplitude, 30 s on, 30 s off, 2 min intervals, for a total of 5 intervals at 4°C. After shearing, DNA was purified using AMPureXP beads (Agencourt), the concentration was checked via Qubit and DNA size was assessed on a Bioanalyzer 2100 using a 1000 DNA Chip. SureSelect XT Library Prep Kit (Agilent) was used to repair DNA ends and for adaptor ligation following the standard protocol. Excess adaptors were removed using AMPureXP beads. Size and concentration were checked again before hybridization. 1 μg of ligated library was used and the standard protocol of the SureSelect XT capture kit was followed to obtain the custom designed Capture-C library. The quality of the captured library was assessed using both Qubit fluorometer and Bioanalyzer's high sensitivity DNA chip. Each SureSelect XT library was initially sequenced on one lane of HiSeq 4000 machine to generate 100 bp paired end reads for QC purposes. All Capture-C libraries were then sequenced three at a time on an S2 flow cell on an Illumina NovaSeq machine, generating ~1.6 billion paired-end reads per sample.

ChIP-Seq Data Processing

Penn Cohort 1

Quality of the raw sequence data was assessed using FastQC. Low-quality base calls and sequencing adapters were trimmed using Trim Galore! with the following parameters: -stringency 5 -length 50 -q 20. Reads were then aligned to the reference human genome (hg19) using the BWA-MEM algorithm.[28] Aligned reads were sorted and filtered based on a minimum mapping quality of 10 using SAMtools-1.3.1.[29] MACS2[30] was used to call peaks for each individual ChIP data using the following parameters:–nomodel–extsize 147 -q 0.01 and the corresponding input data as control. Samples that both had fraction of reads in peaks (FRiP) ≥ 1% as suggested previously[31] and that displayed a significant overlap (i.e., right tailed Fisher's $p < 10^{-6}$ and at least 2-fold enrichment) with ENCODE DNaseI Hypersensitive sites (the ENCODE DNaseI Hypersensitive site master list generated by the ENCODE Analysis Working Group downloaded in October 2016) were retained for the downstream analyses (Table S1). See Figure S1 for the heatmap plot of Spearman's correlation of normalized and averaged ChIP-seq read counts for 27 samples that passed the ChIP-Seq QC thresholds. To generate Figure S1, deepTools[32] was used to normalize the ChIP-Seq read counts to 1× depth of coverage while excluding chromosome X and average scores were calculated based on 10 kb bins that consecutively cover the entire genome.

To define the final set of ChIP-Seq peaks, ChIP-Seq data from biological replicates ($n = 9$ for H3K4me3 and $n = 18$ for H3K27ac) as well as their corresponding input data ($n = 9$ for Input of H3K4me3 and $n = 18$ for Input of H3K27ac) were pooled into separate groups. See Figure S2 for heatmap and profile plots of read density signal around TSS (based on GENCODE v19 annotations) and Figure S3 for correlation between read density signal around TSS and gene expression levels. MACS2[30] was used to call the peaks on the pooled ChIP-seq data of 9 and 18 individuals respectively while using the corresponding input data as control. Among peaks that were called, 68,600 H3K4me3 and 131,293 H3K27ac peaks that have a mean read count of at least 20 were included in further analyses. See Table S2 for chromosomal positions of the peaks, Figure 2A for distribution of peak lengths, and Figure S4 for genomic annotation of ChIP-Seq peaks. Genomic annotations were obtained using Bioconductor's GenomicFeatures package[33] and based on GENCODE v.19 annotations. H3K4me3 peaks were significantly enriched near promoter regions (≤3 kb to TSSs) relative to 1,000 sets of randomly selected size-matching regions of the genome (one-side Fisher's exact test $p < 2.2 \times 10^{-16}$ when the observed overlap was compared with the mean overlap of 1,000 permutations). H3K4me3 and H3K27ac peaks identified also displayed significant enrichment for ENCODE DNase and FAIRE open chromatin regions as well as ENCODE H3K4me3 and H3K27ac sites in HepG2 cells ($p < 2.2 \times 10^{-16}$; Figure S5). Links to ENCODE datasets used are included in Table S5. ENCODE datasets were intersected with liver histone peaks as well as 1,000 sets of randomly selected size-matching regions. Fisher's exact test was used to compare the observed number of overlap with the mean overlap of 1,000 sets of randomly selected size-matching regions.

RNA-Seq Data Processing

Penn Cohort 1

One outlier sample with fewer than one million reads was excluded from the analysis. Quality of the raw sequence data was assessed using FastQC. Low-quality base calls and sequencing adapters were trimmed using Trim Galore! with the following parameters: -stringency 5 -length 50 -q 20 --paired. Trimmed reads were aligned to the reference human genome (hg19) as implemented in STAR aligner[34] using (1) genome indexes based on GENCODE v.19 annotations and (2) genome indexes based on discovered as well as expressed and annotated splice junctions. Specifically, STAR v.2.5[34] was run in two-pass

mode using the following parameters: --outFilterMultimapNmax 10 --outFilterMismatchNmax 10 --outFilterMismatchNoverLmax 0.3 --alignIntronMin 21 --alignIntronMax 0 --alignMatesGapMax 0 --alignSJoverhangMin 5 --twopassMode Basic --twopass1readsN 500000000 --sjdbOverhang 124. Aligned reads were sorted and filtered to retain only primary aligned reads using SAMtools-1.3.1.[29] See Figure S6 for a histogram of number of primary aligned reads. RSEM[35] was used to estimate gene-level expression as transcripts per million (TPM). 19,133 genes with RSEM expected read count of >6 and TPM of >0.1 in at least 10% of the subjects were defined as expressed. TPM values of the expressed genes were natural log transformed after adding a pseudocount of 1. After log transformation, expression values were quantile normalized between individuals across all expressed genes. For each gene, expression values were then inverse quantile normalized to a standard normal distribution across individuals.

Penn Cohort 2

RNA-seq data of Penn Cohort 2 was processed the same way as in Penn Cohort 1. 19,537 genes with RSEM expected read count of >6 and TPM of >0.1 in at least 10% of the subjects were defined as expressed in this cohort.

GTEx

Low-quality base calls and sequencing adapters were trimmed using Trim Galore!. Trimmed reads were aligned to the reference human genome (hg19) as implemented in STAR aligner[34] using (1) genome indexes based on GTEx's GENCODE v.19 gene level annotations; gencode.v19.genes.v6p_model.patched_contigs.gtf and (2) genome indexes based on discovered as well as expressed and annotated splice junctions. STAR v.2.5[34] was run in 2-pass mode using the same parameters as in Penn Cohorts 1 and 2 except the parameter–sjdbOverhang 75 to correspond to the RNA-seq read length of this cohort. Similarly, aligned reads were sorted and filtered to retain only primary aligned reads using SAMtools-1.3.1[29]. RSEM[35] was used to estimate gene-level expression as TPM. 22,415 genes with RSEM expected read count of >6 and TPM of >0.1 in at least 10% of the subjects were defined as expressed.

Genotype Data Processing

Penn Cohort 1

Genotype data were subjected to standard QC checks using whole-genome association analysis toolset PLINK.[36] First, genetic sex of individuals was compared to the self-reported sex. Out of 50 subjects, 1 had inconsistency between self-reported (male) and genotyped (female) sex, but the subject was retained in the study because genotype data were concordant when genotypes based on genotyping array and RNA-seq data were compared. Next, variants with HWE $p < 10^{-6}$ and variants with more than 5% missing rate were excluded. QC'ed genotype data were phased and imputed with SHAPEIT2[37] and IMPUTE2,[38] respectively, using multi-ethnic panel reference from 1000 Genomes Project Phase 3.[39] Following imputation, variants with HWE $p < 10^{-6}$, missing rate > 5%, minor allele frequency (MAF) < 5%, and imputation info score < 0.4 were excluded. This yielded in a total of 4,584,583 imputed variants.

Penn Cohort 2

Genotype data of Penn Cohort 2 was processed the same way as in Penn Cohort 1. Out of 96 subjects, 2 had inconsistencies between their self-reported (female) and genotyped (male) sex. Both subjects were retained in the study after making sure the genotypes based on genotyping array and RNA-seq data were concordant. After imputation and QC checks, 4,541,981 variants were retained.

GTEx

Genotype data was phased and imputed as described previously.[25] Variants with HWE $p < 10^{-6}$, missing rate < 5%, MAF < 5%, and imputation info score < 0.4 were excluded. 5,598,884 variants were retained after imputation and QC filtering.

Genome-wide Promoter-Focused Capture-C Data Processing

Paired-end reads were pre-processed with the HICUP pipeline,[40] with bowtie2 as aligner and hg19 as reference genome. Significant interactions at 1-DpnII fragment resolution were called using CHiCAGO,[41] an open-source package that is commonly used for detection of robust chromatin-chromatin interactions. For this analysis, CHiCAGO was run using default parameters except for binsize, which was set to 2,500. The 4-cutter restriction enzyme, DpnII, yields high-resolution fragments (median fragment size = 264 bp, mean size = 433 bp) compared to the HindIII 6-cutter (median fragment size = 2,274 bp, mean fragment size = 3,697 bp), which is commonly used in comparable Hi-C-based approaches, but sequencing reads are distributed across many fragments, leaving fewer reads available per fragment to call significant promoter contacts, especially when further from the bait. In order to identify additional distal contacts, we also called interactions at the lower 4-DpnII fragment resolution (median fragment size = 1,440 bp, mean fragment size = 1,736 bp), which is still substantially higher than the HindIII resolution. To this end, we proceeded as described in Chesi et al.[42] and according to recommendations in the CHiCAGO vignette.[41] Namely, we generated artificial .baitmap and .rmap files where DpnII fragments were grouped into four consecutively and used these files to run CHiCAGO with default parameters, except for binsize, which was set to 10,000 and removeAdjacent, which was set to False. Results from the two resolutions were merged by taking the union of the interaction calls at either resolution and removing any 4-fragment interaction which contained a 1-fragment interaction.

Estimating Population Structure

Principal component analysis (PCA) as implemented in EIGENSOFT[43] was performed using the genotype data of each cohort in aggregate with HapMap Phase 3 genotype data from 1,184 individuals from 11 populations (ASW, African ancestry in Southwest USA; CEU, Utah residents with Northern and Western European ancestry from the CEPH collection; CHB, Han Chinese in Beijing, China; CHD, Chinese in Metropolitan Denver, Colorado; GIH, Gujarati Indians in Houston, Texas; JPT, Japanese in Tokyo, Japan; LWK, Luhya in Webuye, Kenya; MEX, Mexican ancestry in Los Angeles, California; MKK, Maasai in Kinyawa, Kenya; TSI, Toscani in Italia; YRI, Yoruba in Ibadan, Nigeria).[44] In Penn Cohort 1, 34 of 50 individuals clustered with the HapMap European populations, 12 of them clustered with the HapMap African populations, and the remaining 4 individuals displayed mixed genetic ancestry (Figure S7). In Penn Cohort 2, 62 of 96 individuals clustered with the HapMap European populations, 24 of them clustered with the HapMap African populations, and the remaining 10 individuals displayed mixed genetic ancestry (Figure S8). In GTEx, 81 of 96 individuals clustered with the HapMap European populations, 12 of them clustered with the HapMap African populations, 1 individual clustered with the HapMap Asian populations, and the remaining 2 individuals displayed mixed genetic ancestry (Figure S9).

Mapping *cis*-Expression Quantitative Trait Loci (*cis*-eQTLs)

cis-eQTLs were mapped by linear regression as implemented in FastQTL v2.184.[45] Associations between total expression level (normalized TPM values) of each autosomal gene and variants within 1 Mb of the transcription start site (TSS) were tested within each cohort while adjusting for sex, first three genotype-based PCs, and PEER factors.[46] In the GTEx cohort, genotyping platform was additionally included as a covariate in eQTL mapping. The most suitable effective number of PEER factor was determined to be 5, 22, and 16 for Penn Cohort 1, Penn Cohort 2, and GTEx cohorts, respectively (Figure S10). Nominal p values between each variant and gene pair within 1 Mb of the TSS were calculated by measuring the Pearson product-moment correlation coefficients and using standard significance tests for Pearson correlation.[45] To identify the most significantly associated variant per gene, adjusted p values were estimated by beta approximation method using the parameter "–permute 10000." Genome-wide significance was determined by correcting the adjusted p values for multiple testing across genes using Benjamini&Hochberg method (FDR < 0.05 were considered significant).

METAL[47] was used for meta-analysis of *cis*-eQTL mapping by combining nominal p values across three cohorts while taking the sample size and direction of effect into account. For each gene, the most significantly associated variant per gene (i.e., the one with the smallest meta p value) was recorded to form the empirical, true meta p value distribution. To assess the significance of meta p values, eQTL mapping within each cohort was repeated using permuted gene expression data. METAL was successively run on the permuted eQTL results across three cohorts and permuted meta p values were obtained. For each gene, the most significantly associated variant per gene (i.e., the one with the smallest meta p value) was recorded to form the empirical, null meta p value distribution. Next, FDR of 0.05 was estimated such that Probability(p value0 $<$ z)/ Probability(p value1 $<$ z) $=$ 0.05, where Probability(p value0 $<$ z) is the fraction of p values from the null meta p value distribution that fall below the p value threshold z and Probability(p value1 $<$ z) is the corresponding fraction in the true meta p value distribution (See Figure S11 for QQ-plots of meta *cis*-eQTL associations, Figure S12 for relative distance of meta *cis*-eQTLs to their target gene TSS, and Table S4 for significant meta *cis*-eQTL results).

Identification of Shared and Liver-Specific *cis*-eQTLs

Among 2,625 lead *cis*-eQTLs, 2,552 of them were tested in GTEx Analysis Releasev6p. For each of these 2,552 lead *cis*-eQTL-gene expression pairs, posterior probability of association in 43 non-liver GTEx tissues[25] were calculated using METASOFT.[48] *cis*-eQTLs with a posterior probability of 0.9 in at least 38 non-liver tissues were defined as "shared-eQTLs" and *cis*-eQTLs with a posterior probability of 0.9 in fewer than 5 non-liver tissues were defined as "liver-specific eQTLs." Overlap between these three sets of *cis*-eQTLs (total, shared, and liver-specific) and H3K4me3 and H3K27ac peaks were identified using bedtools intersect –u function. Liver H3K4me3 and H3K27ac peaks identified in this study as well as those of ENCODE consortium (links to ENCODE datasets are in Table S5) were included in this analysis. For each *cis*-eQTL set, 1,000 matching SNP sets (LD of r^2 0.5, MAF of $\pm$5%, gene density of $\pm$5%, distance to nearest gene of $\pm$50%, LD buddies of $\pm$50% in European 1000G Phase3) were obtained using SNPsnap.[49] Odds ratios of observed/expected overlaps were plotted in Figures S13 and 3B.

Mapping *trans*-Expression Quantitative Trait Loci (*trans*-eQTLs)

Associations between each expressed autosomal protein coding gene and variants that are more than 5 Mb apart were considered as *trans*. *trans*-eQTLs were mapped using MatrixeQTL[51] while adjusting for the same covariates that were used in *cis*-eQTL mapping (i.e., sex, ancestry, PEER factors in each cohort as well as genotyping platform in GTEx cohort). *trans*-eQTL mapping was performed within each cohort using (1) all linkage disequilibrium pruned variants ($r^2 > 0.5$, plink parameters –indep 50 5 2 across cohorts), (2) variants that were identified as *cis*-eQTLs in this study (2,625 variants based on meta *cis*-eQTL results), and (3) variants that are likely to affect transcription factor activity. In approach 1, our goal was to perform a hypothesis-free genome-wide *trans*-eQTL scan. In approach 2, we hypothesized that *cis*-eQTLs through regulation of gene expression can alter protein levels of their *cis*-eGenes and differences in protein levels can affect expression levels of other genes downstream. In approach 3, we hypothesized that *cis*-eQTLs and coding variants of transcription factors are likely to alter transcription factor activity (either through altering protein level or protein function) and that differences in transcription factor activity can affect expression levels of other genes downstream. For approach 3, we obtained the curated list of 1,988 transcription factors from T. Ravasi et al.[52] We included the significant meta *cis*-eQTLs for this set of genes and protein coding variants of this set of genes (based on gnomAD release-170228) that passed our initial genotyping QC threshold within each cohort (4,997, 4,860, and 5,516 variants for Penn Cohort 1, Penn Cohort 2, and GTEx, respectively).

METAL[47] was used for meta-analysis of *trans*-eQTL mapping by combining nominal p values across three cohorts while taking the sample size and direction of effect into account for each type of *trans*-eQTL approach. Similarly, METAL was run on the permuted *trans*-eQTL results across three cohorts and permuted meta p values were obtained. Permutation-based FDR was calculated as explained in the *cis*-eQTL mapping section above. See Figure S14 for QQ-plots of meta *trans*-eQTL associations. There were two statistically significant *trans*-eQTL findings when genome-wide approach was used. However, both of these results were filtered due to presence of genes near *trans*-eQTL (within 100 kb) with evidence of RNA-seq read cross-mapping due to sequence similarity (Table S12). There were no significant findings when only *cis*-eQTLs or only variants likely to affect transcription factor activity were tested as *trans*-eQTLs.

Mapping *cis*-Histone Quantitative Trait Loci (*cis*-hQTLs)

Picard Tools' MarkDuplicates function was used with the "REMOVE_DUPLICATES = true" parameter set to remove duplicate reads from aligned and q10 filtered ChIP-seq data (initial ChIP-seq data processing and QC steps were explained above in the ChIP-Seq Data Processing section). Genotype data of Penn cohort 1 were further processed to include only single-nucleotide substitutions in hQTL mapping. Allele-specific read counts were obtained with GATK's[53] ASEReadCounter function. Total feature counts and GC% values of each feature were used to generate sample specific offset values for each feature. To generate PEER Factors, FPM values (equivalent of TPM for ChIP-seq data) were calculated, quantile normalized between individuals, and inverse quantile normalized to a standard normal distribution within each peak. Sex, first three genotype-based PCs, and five PEER factors were included as covariates. RASQUAL[20] was used to map hQTL

associations. Variants within 10 kb of each end of a histone peak were considered as *cis*. All autosomal peaks were included in hQTL mapping. This corresponded to a total of 65,649 and 128,822 tested peaks for H3K4me3 and H3K27ac, respectively. For each peak, the most significant p value was selected to form the empirical, true hQTL p value distribution. To assess genome-wide significance, RASQUAL was successively run using the -r/–random-permutation option. For each peak, the most significant p value from the permutation run was selected to form the empirical, null hQTL p value distribution. Next, FDR of 0.05 was estimated such that Probability(p value0 $<$ z)/ Probability(p value1 $<$ z) = 0.05, where Probability(p value0 $<$ z) is the fraction of p values from the null hQTL p value distribution that fall below the p value threshold z and Probability(p value1 $<$ z) is the corresponding fraction in the true hQTL p value distribution. Peaks with significant hQTLs were excluded if they had potential reference mapping bias ($\phi < 0.25$ and $\phi > 0.75$). See Table S7 for significant hQTL results.

Integrative Analyses of Liver *cis*-hQTLs with Other Functional Datasets

We used ENCODE Regulation "Txn Factor" track and downloaded the wgEncodeRegTfbsClusteredWithCellsV3.bed.gz file, which includes transcription factor binding site clusters together with the input cell sources. Using this file, we extracted the binding sites of 61 transcription factors that were obtained in HepG2 cell line. Link to the ENCODE dataset used is included in Table S5. Peaks with *cis*-hQTLs were overlapped with 61 different transcription factors' binding sites in HepG2 cells using bedtools intersect –u function. Enrichment of overlap was calculated relative to 1,000 sets of randomly chosen matching numbers of H3K4me3 and H3K27ac autosomal peaks in our data. One-sided Fisher's exact test was used to assess the significance of the enrichment.

For 151 of the hQTL-peaks, target interacting gene promoters could be identified using the Capture-C data. For each variant that was located within such hQTL-peak, *cis*-eQTL p value for the interacting gene was pulled to form the distribution of observed *cis*-eQTL p values. This observed p value distribution was then compared to the expected distribution of p values that was observed when 151 histone peaks with no hQTLs were chosen randomly from the set of autosomal histone peaks that do not have significant hQTLs. In a complementary analysis, the proportion of eQTL-genes among the 210 genes that interact with hQTL-peaks was compared with the proportion that was observed among 1,000 sets of randomly chosen 210 genes that were baited and expressed but that do not interact with hQTL-peaks. Significance was assessed based on the permutation p value.

Identification of Shared Genetic Signals Underlying Variation in Histone Modification States and Gene Expression Levels

For each gene with a significant meta *cis*-eQTL, H3K4me3 and H3K27ac peaks with significant hQTLs that are located within 1 Mb of its transcription start site (TSS) were tested for evidence of co-regulation. Gene-peak pairs with $r^2 > 0.8$ between the lead hQTL and lead eQTL were considered as putatively co-regulated. r^2 was calculated in the 1000 Genomes, Phase 3, European population. Among significant co-regulation results, the ones in the MHC region (chr6: 28,510,120–33,480,577) were excluded owing to complicated LD patterns of this locus. We note that hQTLs were mapped using a subset of Penn Cohort 1 samples and eQTLs were mapped across the three cohorts in our study. While the majority of the individuals in our cohorts were of European ancestry (Figures S7–S9), we suggest that caution be taken, as there could be differences in LD in specific genomic regions when genotype data of our study subjects are compared to 1000 Genomes, Phase 3, European population.

Identification of Trait-Relevant Genes and Regulatory Elements in GWAS Loci

GWAS summary statistics of 20 phenotypes including coronary artery disease,[54] HDL cholesterol,[55] LDL cholesterol,[55] total cholesterol,[55] triglycerides,[55] diastolic blood pressure,[56] systolic blood pressure,[56] mean arterial pressure,[56] rheumatoid arthritis,[57] type 2 diabetes,[58] multiple sclerosis,[59] asthma,[59] psoriasis,[59] Parkinson disease,[59] Alzheimer disease,[60] schizophrenia,[61] Crohn disease,[62] ulcerative colitis,[62] inflammatory bowel disease,[62] and age-related macular degeneration[63] were obtained (see Table S9 for links to datasets). Among GWAS variants with $p < 10^{-6}$, most significant variant was chosen to represent each 2 Mb region and to define each significant GWAS locus, 1 Mb upstream and 1 Mb downstream of the lead variant. Approximate Bayes Factor colocalization analysis of the coloc package[64] was performed between each eQTL-gene whose TSS was within each GWAS locus and the corresponding GWAS phenotype. To assess the significance of colocalization analysis,[64] we used a previously published approach[16] and assessed whether there was sufficient power to test for colocalization (PP3+PP4 $>$ 0.8), and for the colocalization pairs that pass the power threshold, we defined PP4/(PP3+PP4) $>$ 0.9 as the significant colocalization threshold. Similarly, significant hQTL-peaks that were located within each GWAS locus were tested for evidence of underlying GWAS signals. When lead hQTLs and lead GWAS variants were in high linkage disequilibrium ($r^2 > 0.8$), we considered such hQTL-peaks as the likely trait-relevant regulatory elements in GWAS loci. r^2 was calculated in the 1000 Genomes, Phase 3, European population. All significant signals in the MHC region (chr6: 28,510,120–33,480,577) were excluded owing to the complicated LD patterns of this locus.

In loci where we identified a single colocalized gene, we classified whether the candidate genes prioritized in our study have been previously reported as likely trait-relevant genes for the phenotype of interest or not. To do so, we downloaded the NHGRI-EBI GWAS Catalog in May 2018. We retrieved the entries that matched the GWAS phenotype (column "MAPPED_TRAIT") as well as chromosome nomenclature of the GWAS locus under question (column "REGION"). For each retrieved entry, we checked the hg19 genomic position of the SNP reported (column "SNPs") and pulled entries that are located within the GWAS locus of interest (within 1 Mb to GWAS lead variant). Among the selected entries, we checked the proportions of studies that reported the colocalized gene as the sole versus among many candidate genes of interest (column "REPORTED.GENE.S"). Colocalized genes that have not been reported as candidate GWAS genes in NHGRI database were further retrieved in PubMed database using search terms that included the colocalized gene ID in combination with GWAS phenotype. Each retrieved manuscript was then evaluated to determine whether colocalized gene has been previously reported as candidate GWAS gene for the phenotype of interest or not. We note that our literature review may be incomplete as the review was performed by a single author and was limited to manuscripts that were written in English and those that were open

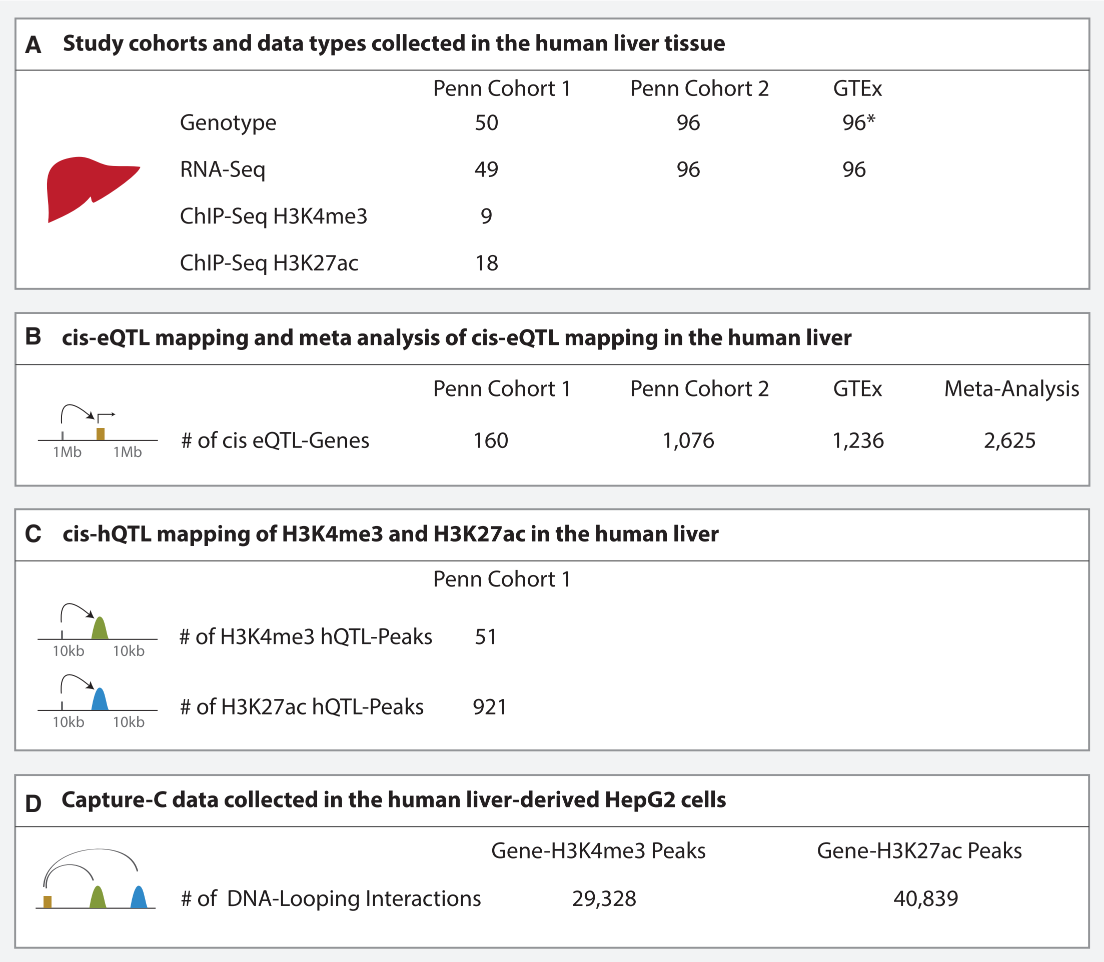

Figure 1. Descriptions of the Study Cohorts and Datasets Collected
(A) Subjects from three cohorts were included in this study. Penn cohort 1 and Penn cohort 2 samples were collected at the Penn Transplant Institute and datasets from these two cohorts have not been published previously. GTEx liver samples were collected as a part of the GTEx Analysis Releasev6p.[25]
(B) For eQTL mapping, associations between variant-gene pairs that are within 1 Mb of the TSS were considered as *cis*. *cis*-eQTLs were mapped within each cohort and a meta-analysis was performed across cohorts. Numbers of genes with a significant *cis*-eQTL at an FDR of 5% are shown.
(C) For hQTL mapping, associations between histone peaks and variants within 10 kb of the nearest end of the histone peaks were considered as *cis*. Numbers of histone peaks with a significant *cis*-hQTL at an FDR of 5% are shown.
(D) Genome-wide promoter-focused Capture-C was used to identify gene promoter-histone peak interactions. 29,328 gene promoter-H3K4me3 peak and 40,839 gene promoter-H3K27ac peak interactions were identified at CHiCAGO score[41] of $\geq$ 5. *DNA from GTEx liver samples were extracted from whole blood.

access and/or could be retrieved through Penn Library-licensed electronic resources.

Results

Annotation of Regulatory Elements and Their Interacting Gene Promoters

H3K4me3 and H3K27ac are epigenetic histone modifications that are enriched in functional non-coding regions of the human genome, including active gene promoters and enhancers.[65–67] We performed chromatin immunoprecipitation (ChIP)[68] for H3K4me3 and H3K27ac modifications in human liver tissue, sequenced ChIP-ed DNA, and obtained H3K4me3 and H3K27ac ChIP-seq data from 9 and 18 individuals, respectively (Figure 1A and Table S1, see Material and Methods for all cohort and analysis details). Using these data, we annotated 68,600 and 131,293 genomic regions enriched for H3K4me3 and H3K27ac modifications (i.e., ChIP-seq peaks; Figure 2A and Table S2). Similar to previous reports,[65–67] we showed that H3K4me3 is highly enriched ($p < 2.2 \times 10^{-16}$) near transcription start sites (TSSs) and H3K27ac is approximately equally present within intronic, distal intergenic, and near-TSS regions (Figure 2B). We also collected genome-wide genotype and RNA-seq data in the liver from two cohorts and, alongside with publicly available GTEx v6p liver data,[25] we studied the extent of inter-individual variation in liver gene expression levels in a total sample size of 241 (Figure 1A). Across the three cohorts in our study, we identified 23,271 expressed genes. Using a genome-wide

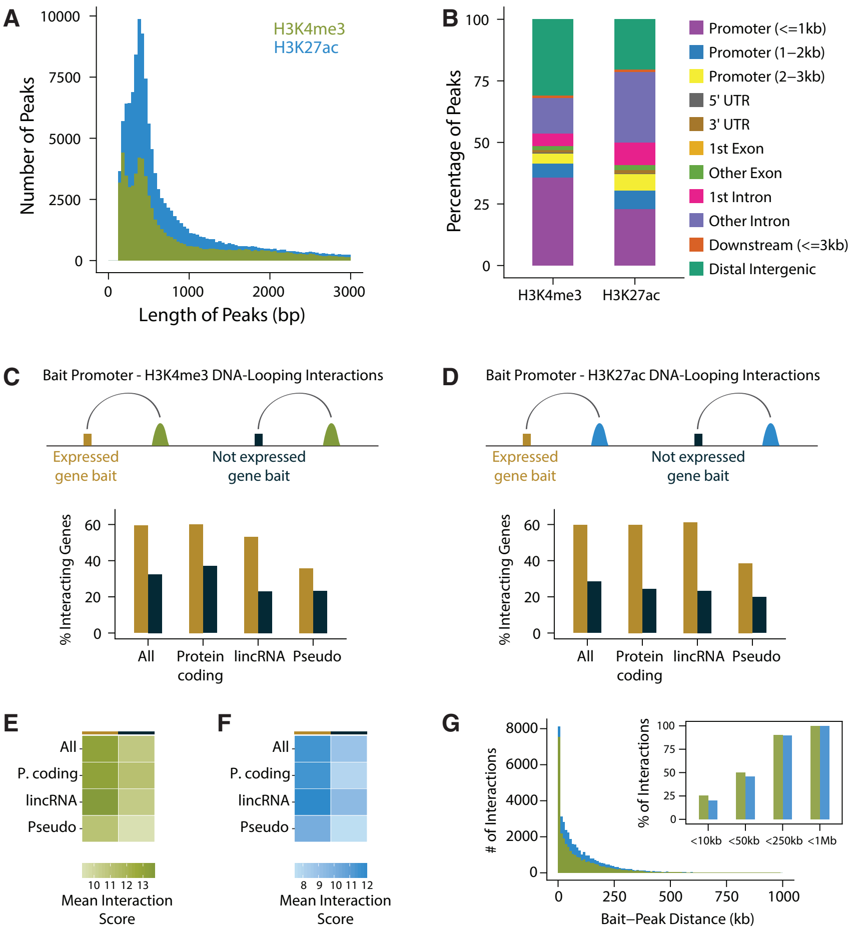

Figure 2. Annotation of Regulatory Elements and Their Interacting Gene Promoters

(A) Distribution of H3K4me3 (green) and H3K27ac (blue) peak lengths. Median peak lengths were 507 bp and 491 bp for H3K4me3 and H3K27ac peaks, respectively. Note that this zoomed-in plot does not display peaks with length >3,000 bp.

(B) Genomic annotations of the 68,600 H3K4me3 and 131,293 H3K27ac ChIP-seq peaks.

(C) Percentage of baited gene promoters that form DNA-looping interactions with H3K4me3 peaks. Expressed genes were significantly more likely to form DNA-looping interactions with H3K4me3 peaks. Pearson's chi-square test p values were $< 2.2 \times 10^{-16}$ for all, protein-coding, lincRNA gene groups and 0.00036 for pseudogenes.

(D) Percentage of baited gene promoters that form DNA-looping interactions with H3K27ac peaks. When expressed genes were compared with genes that were not detected as expressed, Pearson's chi-square test p values were $< 2.2 \times 10^{-16}$ for all, protein-coding, lincRNA gene groups and 4.2×10^{-8} for pseudogenes.

(E) Mean CHiCAGO interaction scores between baited gene promoters and H3K4me3 peaks. Interaction scores between H3K4me3 peaks and expressed genes were significantly higher than those between H3K4me3 peaks and genes that were not detected as expressed. One-tailed Welch two sample t test p values were $< 2.2 \times 10^{-16}$, 5.34×10^{-6}, 0.0061, 0.084 for all, protein-coding, lincRNA, and pseudogenes, respectively.

(F) Mean CHiCAGO interaction scores between baited gene promoters and H3K27ac peaks. Interaction scores between H3K27ac peaks and expressed genes were significantly higher than those between H3K27ac peaks and genes that were not detected as expressed. One-tailed Welch two sample t test p values were $< 2.2 \times 10^{-16}$, $< 2.2 \times 10^{-16}$, 6.3×10^{-5}, 8.6×10^{-4} for all, protein-coding, lincRNA, and pseudogenes, respectively.

(G) Distribution of distance between interacting bait promoters and histone peaks. >99% of all interacting bait-peak pairs were within less than 1 Mb apart.

promoter-focused Capture-C method (approach was derived from Hughes et al.;[27] see Material and Methods for details), we identified 29,328 significant[41] promoter-H3K4me3 and 40,839 promoter-H3K27ac peak interactions within the liver-derived HepG2 cell line (Figure 1D and Table S3). The promoters of expressed genes were significantly more likely to have DNA looping interactions with the histone peaks identified in our study relative to the promoters of genes that were not expressed ($p < 2.2 \times 10^{-16}$; Figures 2C and 2D). Furthermore, the interactions between expressed gene promoters and the liver histone peaks were significantly stronger than those observed between histone peaks and genes that were not expressed ($p < 2.2 \times 10^{-16}$; Figures 2E and 2F). More than 99% of the histone peaks with evidence of looping were within 1 Mb of their interacting promoters (Figure 2G).

Identification of Shared and Liver-Specific *cis*-eQTLs

We mapped *cis*-expression quantitative trait loci (*cis*-eQTL; here defined as associations between the expression level of a gene and a variant within 1 Mb of the gene TSS) within each cohort by linear regression[45] and performed a meta-analysis[47] across three cohorts. We identified 2,625 genes with *cis*-eQTLs at 5% FDR; we hereafter refer to such genes as eQTL-genes (Figure 1B and Table S4). For each *cis*-eQTL identified, we estimated the posterior probability[48] that the eQTL effect is present in 43 non-liver GTEx v6p tissues[25] (Figure 3A and Table S4). We classified *cis*-eQTLs that have a posterior probability of greater than 0.9 for being an eQTL in at least 38 non-liver tissues as "shared eQTLs" and those with a posterior probability of greater than 0.9 in fewer than five non-liver tissues as "liver-specific eQTLs" (the last and first quartiles of the distribution in Figure 3A, respectively). We integrated these *cis*-eQTL findings with H3K4me3 and H3K27ac peaks that we identified in the human liver as well as those that were identified in multiple cell lines by the ENCODE consortium[5] (Table S5). Overall, *cis*-eQTLs were significantly more likely to overlap H3K4me3 and H3K27ac histone peaks relative to randomly selected SNPs matched for key properties

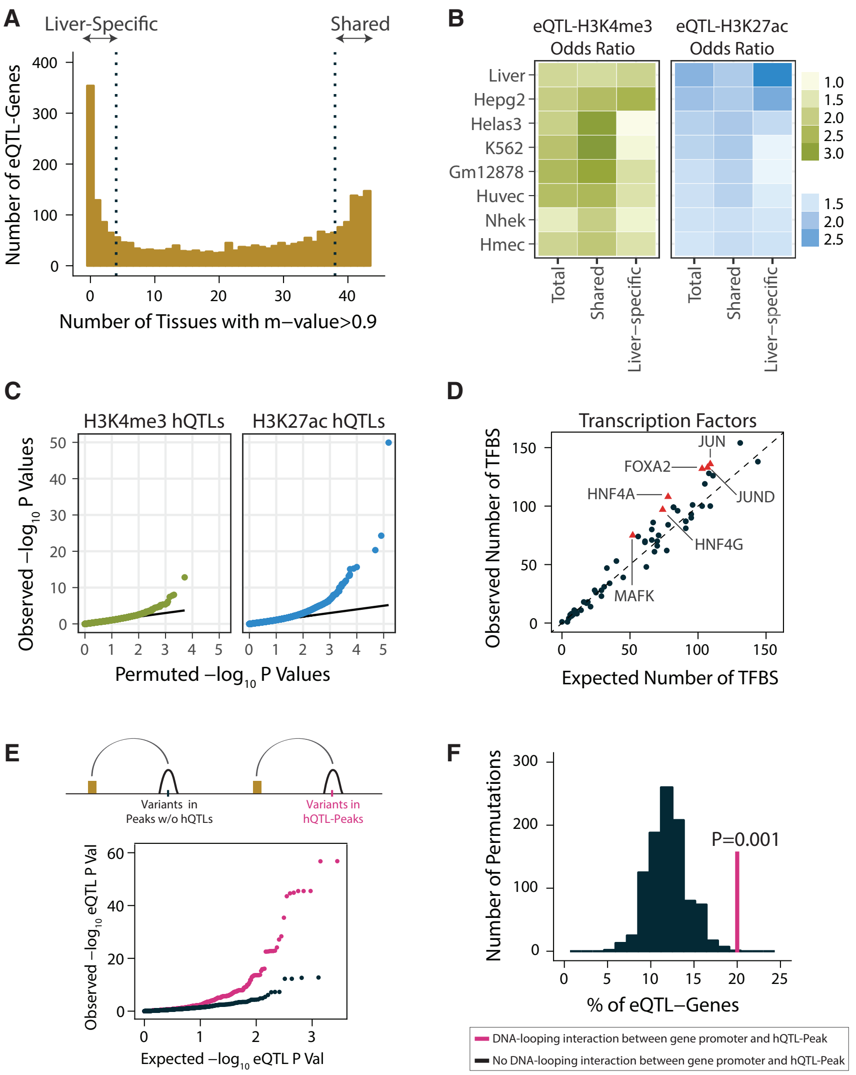

Figure 3. Identification of Expression and Histone Quantitative Trait Loci in the Human Liver

(A) Distribution of the number of non-liver GTEx tissues with an association posterior probability (m-value) of > 0.9 for lead *cis*-eQTL-gene pairs. *cis*-eQTL-gene pairs in the first quartile of the distribution were considered as liver-specific, those in the last quartile were considered as shared eQTLs.

(B) Overlap between *cis*-eQTL sets (total, shared, and liver-specific) and H3K4me3 and H3K27ac peaks. Odds ratios are relative to randomly chosen matching (with respect to LD, MAF, gene density, distance to nearest gene) SNP sets. H3K4me3 and H3K27ac data from non-liver tissues were obtained from ENCODE database, links to the ENCODE data files are included in Table S5. The odds ratios are only plotted for cell lines with both H3K4me3 and H3K27ac data. Results from other cell types are included in Figure S13, p values and odds ratios are included in Table S6.

(C) QQ-plots of the *cis*-hQTL association p values of H3K4me3 (panel 1) and H3K27ac (panel 2). Solid lines represent the expected distribution of p values based on permuted data.

(D) Observed and expected numbers of transcription factor binding sites (TFBS) in hQTL-peaks. Expected numbers represent the mean TFBS overlap of 1,000 set of randomly chosen matching numbers of autosomal liver histone peaks. Transcription factors that were significantly enriched (one-tailed Fisher's exact test $p < 0.05$) in hQTL-peaks are shown in red triangles.

(E) hQTL-peaks were assigned to their interacting gene(s) using chromatin capture data. For each variant within a hQTL-peak, its eQTL-pvalue on the interacting gene(s) is plotted in magenta color. Matching numbers of peaks were drawn from the set of expressed and baited genes that do not interact with hQTL-peaks. eQTL p values of the variants within the background set of histone peaks on their interacting gene(s) are shown in black color. One-sided Wilcoxon rank sum test p value comparing two p value distributions was 3.75×10^{-9}.

(F) Percentage of eQTL-genes among genes that interact with hQTL-peaks is shown in the magenta vertical line. Distribution of expected percentage based on 1,000 sets of randomly chosen matching numbers of genes that do not interact with hQTL-peaks are shown in black. p value of 0.001 is based on the permutation test.[50]

including linkage disequilibrium (LD), minor allele frequency (MAF), gene density, and distance to nearest gene (Figure 3B and Table S6). Moreover, shared eQTLs overlapped H3K4me3 promoter marks more often than liver-specific eQTLs (Figure 3B and Table S6). Conversely, while shared eQTLs showed similar levels of overlap with H3K27ac enhancer marks across different tissues, liver-specific eQTLs were significantly more likely to overlap H3K27ac marks that we identified in human liver tissue as well as those that were identified in liver-derived HepG2 cell lines, consistent with the previous reports of significantly higher cell-type specificity of enhancers relative to promoters[69] (Figure 3B and Table S6). Despite implementing multiple approaches, we did not identify any significant *trans*-eQTLs in the human liver (see Material and Methods for details).

Identification of *cis*-hQTLs in the Human Liver

To identify genetic determinants of H3K4me3 and H3K27ac modifications in the liver, we applied a method that uses both total and allele-specific signals in sequencing data to enable quantitative trait loci (QTL) mapping with relatively small sample sizes.[20] We identified *cis*-QTLs for 51 H3K4me3 and 921 H3K27ac peaks at 5% FDR (Figures 1C and 3C and Table S7). We refer to such variants as histone QTLs (hQTLs) and the peaks that they regulate as hQTL-peaks throughout the manuscript. We intersected the hQTL-peaks with transcription factor binding sites (TFBSs) that were obtained in HepG2 cells by the ENCODE consortium (Table S5).[5] We found that liver hQTL-peaks are significantly enriched for binding sites of hepatocyte nuclear factors (HNF4A, HNF4G, FOXA2) as well as transcription factors (TF) involved in hepatocellular

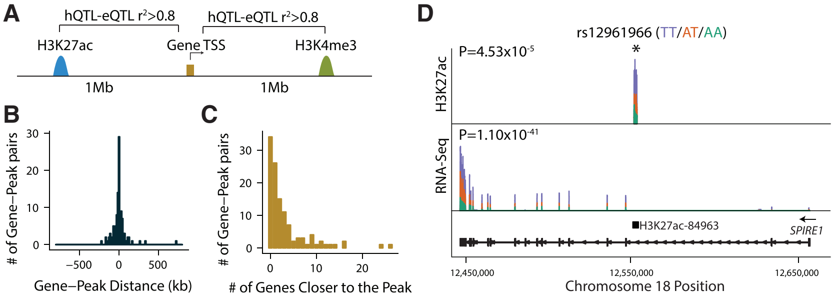

Figure 4. Putatively Co-regulated Histone Modification States and Gene Expression Levels

(A) For each gene with a significant meta *cis*-eQTL, H3K4me3 and H3K27ac peaks with significant hQTLs that are located within 1 Mb of its transcription start site (TSS) were tested for evidence of co-regulation. Gene-peak pairs with $r^2 > 0.8$ between the lead hQTL and lead eQTL were considered as putatively co-regulated.

(B) Distance between putatively co-regulated gene-peak pairs.

(C) Distribution of number of genes that are closer to the hQTL-peak than its putatively co-regulated eQTL-gene.

(D) Example of a putatively co-regulated gene-peak pair. SNP rs12961966 was significantly associated with chromatin modification state of an enhancer (H3K27ac-84963; chr18: 12,551,731–12,553,678) residing in the second intron of *SPIRE1* and *SPIRE1* expression level. Sushi plots[70] show the mean normalized read counts of each genotype group. Sample sizes of each genotype group were TT:4, AT:8, AA:5 for ChIP-seq data and TT:55, AT:94, AA:88 for RNA-seq data. *SPIRE1* model shown below the sushi plots was generated using ggbio Bioconductor package[71] and *SPIRE1* transcript ENST00000409402. Boxplots of normalized H3K27ac-84963 ChIP-seq and *SPIRE1* RNA-seq read counts are stratified by genotype at the rs12961966 are displayed in Figure S16.

remodeling (JUN and JUND) when compared with randomly selected matching numbers of liver histone peaks from our data ($p < 0.05$; Figure 3D). Furthermore, using our chromatin capture data, we found that variants within hQTL-peaks were more likely to be significantly associated with the expression of genes with which they are in contact with relative to the variants within histone peaks that do not have hQTLs ($p = 3.75 \times 10^{-9}$, Figure 3E). Overall, genes that interact with an hQTL-peak were almost twice as likely to have *cis*-eQTLs relative to randomly selected matching numbers of expressed and baited genes that do not interact with hQTL-peaks ($p = 0.001$; Figure 3F). These results suggest that genotype-dependent putative functional elements identified here play causal roles in the regulation of gene expression levels and this, at least in part, is mediated via DNA looping interactions.

Putatively Co-regulated Histone Modification States and Gene Expression Levels

Integrating eQTL associations with regulatory element annotations has proven useful for the precise identification of causal regulatory variants and the specific regulatory elements they perturb. Our results highlight the value of analyzing tissue-type-matched gene expression and regulatory element datasets (Figure 3B). These analyses, however, are limited as there are often multiple regulatory elements within each eQTL locus and hence it has remained difficult to systematically link regulatory elements to their respective target genes. To address this, we first identified putatively co-regulated hQTL-peaks and eQTL-genes. Because of the limited sample size of our ChIP-seq data, we identified co-regulated peak-gene pairs based on LD between lead QTL-SNPs (i.e., $r^2 > 0.8$ between the lead hQTL and lead eQTL; Figure 4A). We found 116 gene-peak pairs that are likely regulated by the same causal variant (Table S8). These 116 gene-peak pairs corresponded to 104 unique eQTL-genes and 95 unique hQTL-peaks. hQTL-peaks were often not assigned to their nearest gene; in 71% of the co-regulated gene-peak pairs, there was at least one other gene that is closer to the hQTL-peak than the eQTL-gene with which it is co-regulated (Figures 4B and 4C). Figure 4D displays an example of a putatively genetically co-regulated gene-peak pair (r^2 between lead QTLs = 0.89), supporting the presence of a shared causal effect underlying the activity of an enhancer located in the second intron of *SPIRE1* (H3K27ac-84963; chr18: 12,551,731–12,553,678) as well as *SPIRE1* gene expression level. The full set of putatively genetically co-regulated gene-peak pairs are included in Table S8.

Identification of Trait-Relevant Genes and Regulatory Elements in GWAS Loci

Next, we asked whether leveraging our hQTL, eQTL, and chromatin capture findings could help fine-map GWAS loci. Throughout this manuscript, we defined "fine-mapping" as evidence of refinement in putatively trait-relevant gene, regulatory element, and variant identification in any individual GWAS locus. We obtained GWAS summary statistics of 20 phenotypes that are commonly studied (based on the number of PubMed IDs in the NHGRI-EBI GWAS Catalog) and that have variable levels of suggested causality manifesting in the liver.[2] These phenotypes included coronary artery disease,[54] HDL cholesterol,[55] LDL cholesterol,[55] total cholesterol,[55] triglycerides,[55] diastolic blood pressure,[56] systolic blood pressure,[56] mean arterial pressure,[56] rheumatoid arthritis,[57] type 2 diabetes,[58] multiple sclerosis,[59] asthma,[59] psoriasis,[59] Parkinson disease,[59] Alzheimer disease,[60] schizophrenia,[61] Crohn disease,[62] ulcerative colitis,[62] inflammatory bowel disease,[62] and age-related macular degeneration.[63] Links to the GWAS summary statistics used are included in Table S9. We used a p value threshold of $< 1 \times 10^{-6}$, selected a lead variant to represent each 2 Mb region (1 Mb upstream and 1 Mb downstream of the lead variant), and identified 1,614 loci previously associated with these phenotypes. Genetically regulated gene expression levels and histone modification states in GWAS loci can reveal the

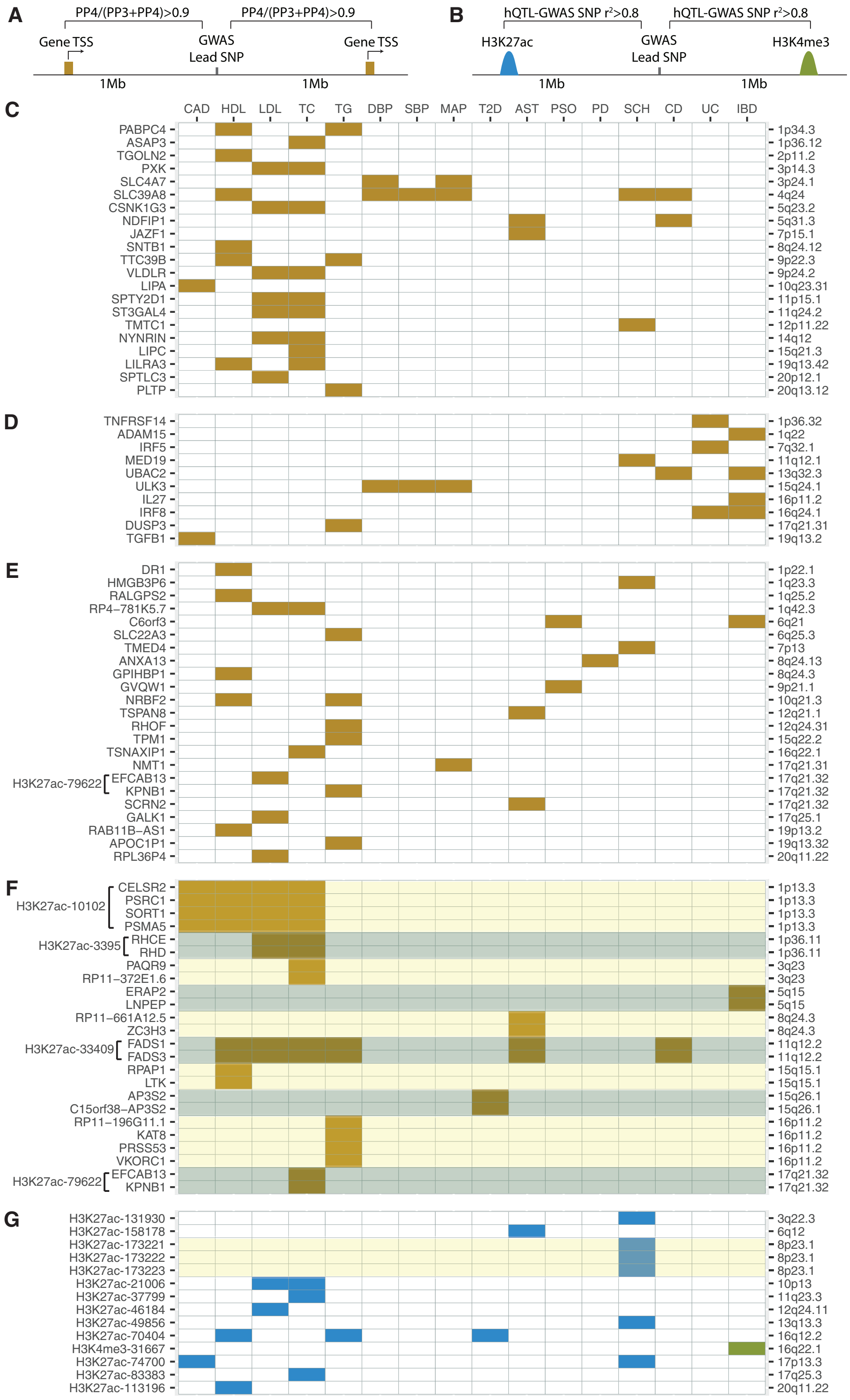

Figure 5. Candidate Trait-Relevant Genes and Gene Regulatory Elements in GWAS Loci
(A) Each GWAS locus was defined as the 2 Mb region around the lead GWAS variant. A Bayesian colocalization approach was performed between the GWAS phenotype and each gene with a significant meta *cis*-eQTL whose TSS resides within the GWAS locus.
(B) An LD threshold of $r^2 > 0.8$ between lead GWAS variants and lead hQTLs was used to identify putatively trait-relevant *cis*-regulatory elements in GWAS loci.

(legend continued on next page)

mechanisms underlying observed associations between genetic variants and disease phenotypes.[7–14] We therefore applied a Bayesian colocalization approach[64] to identify eQTL-genes that likely underlie disease phenotypes (Figure 5A) and used an LD threshold ($r^2 > 0.8$) between lead GWAS variants and lead hQTLs to identify putatively trait-relevant *cis*-regulatory elements in GWAS loci (Figure 5B). To assess the significance of colocalization analysis,[64] we used a previously published approach[16] and first assessed whether there was sufficient power to test for colocalization (PP3+PP4 > 0.8), and for the colocalization pairs that pass the power threshold, we defined the threshold for significance as PP4/(PP3+PP4) > 0.9. In loci where we found at least one eQTL-gene with significant evidence of underlying GWAS associations, we repeated colocalization analysis using all expressed genes within 2 Mb region around each lead GWAS variant. Our rationale was to avoid possibility of excluding genes with moderate eQTL signals, which did not reach genome-wide significance threshold of eQTL mapping but displayed significant evidence of eQTL-GWAS colocalization.

We found a total of 125 GWAS-gene and 33 GWAS-peak pairs with evidence of shared genetic causality (Table S10). For 77 GWAS-gene pairs, our dataset contains evidence supporting identification of the trait-relevant gene, as there was only one gene that significantly colocalized with the GWAS phenotype (Figures 5C–5E). We identified several candidate genes and regulatory elements that underlie associations with more than one GWAS phenotype. For instance, 77 GWAS-gene pairs with only one colocalized gene implicated 54 unique genes (Figures 5C–5E). 21 of these genes were previously reported as the likely trait-relevant gene at the locus[55,56,61,64,72–79] (Figure 5C). For 10 loci, our findings help refine candidate gene identification from among several genes that were suggested to be trait relevant in the literature[56,72,73,76,79–84] (Figure 5D) and in 21 loci, we discovered candidate trait-relevant genes that have not been previously suggested to underlie the corresponding GWAS phenotype (Figure 5E). We were not able to identify any trait-relevant genes for rheumatoid arthritis, multiple sclerosis, age-related macular degeneration, and Alzheimer disease using the data collected in the human liver.

At the 17q21.32 locus, we identified a genetically regulated enhancer (H3K27ac-79622; chr17: 45,733,609–45,733,977) that likely underlies GWAS associations with LDL cholesterol, triglyceride, and total cholesterol levels. We found *EFCAB13* and *KPNB1* (MIM: 602738) as the candidate genes driving associations with LDL and triglyceride levels, respectively (Figure 5E). We were not able to distinguish the effects of these two genes with regard to total cholesterol associations (Figure 5F). The colocalization probabilities of these two genes were close to the significance threshold for all three phenotypes, suggesting that there is insufficient signal to discriminate the two genes using colocalization analysis. The enhancer identified in this locus, however, was only forming DNA-looping interactions with the promoter of *KPNB1*, and the direction of effect on enhancer activity was only consistent with *KPNB1* expression, suggesting that *KPNB1* is the likely trait-relevant gene in this locus (Figure 6A). Similary, at the chromosome 1p13.3 locus, we reassuringly identified the previously reported trait-relevant enhancer (H3K27ac-10102; chr1: 109,816,977–109,818,871)[11] as the candidate regulatory element responsible for the GWAS associations with coronary artery disease, HDL cholesterol, LDL cholesterol, and total cholesterol levels (Figures 5F and S15). Our chromatin capture interaction data also revealed an interaction between ChIP-seq peak H3K27ac-10102 and the promoter of the *SORT1* (MIM: 602458) gene, supporting the previously reported regulatory role of this enhancer on *SORT1* gene expression (Figure S15).[11]

At the 11q12.2 locus, which has been shown to be a critical component of adaptation to different diets,[86,87] we identified a genetically regulated enhancer (H3K27ac-33409; chr11: 61,587,373–61,589,527) that likely underlies associations with blood lipid phenotypes, asthma, and Crohn disease (Figure 6B). Interestingly, a previous study has reported a genetically regulated DNA methylation probe that overlaps the enhancer we identified and suggested that DNA methylation differences in this putative enhancer affects FADS1 (MIM: 606148) protein activity in the liver.[88] While our colocalization analyses identified both *FADS1* and *FADS3* (MIM: 606150) as candidate trait-relevant genes (Figure 6B), our findings as well as those from Howard et al.[88] support the identification of the trait-relevant regulatory element (H3K27ac-33409; chr11: 61,587,373–61,589,527) at this locus.

Lastly, while we were not able to identify a target gene at the chromosome 16q12.2 locus, we found a genetically regulated putative enhancer in the first intron of *FTO*

(C) GWAS loci with only one significantly colocalized gene and the gene identified has been reported as the likely trait-relevant gene by overwhelming majority of the literature.
(D) GWAS loci with only one significantly colocalized gene and the gene identified has been reported among several genes that were suggested to be trait relevant in the literature.
(E) GWAS loci with only one significantly colocalized gene and the gene identified has not been previously implicated in the corresponding GWAS phenotype.
(F) GWAS loci with more than one significantly colocalized gene. Genes within the same GWAS locus are shown in alternating shades of yellow and blue. When identified, putatively trait-relevant histone peaks are included next to the colocalized gene names of each locus.
(G) GWAS loci where candidate trait-relevant regulatory elements were identified in the absence of colocalized liver eQTL genes.
Phenotype abbreviations are as follows: CAD, coronary artery disease; HDL, HDL cholesterol; LDL, LDL cholesterol; TC, total cholesterol; TG, triglycerides; DBP, diastolic blood pressure; SBP, systolic blood pressure; MAP, mean arterial pressure; T2D, type 2 diabetes; AST, asthma; PSO, psoriasis; PD, Parkinson disease; SCH, schizophrenia; CD, Crohn disease; UC, ulcerative colitis; IBD, inflammatory bowel disease.

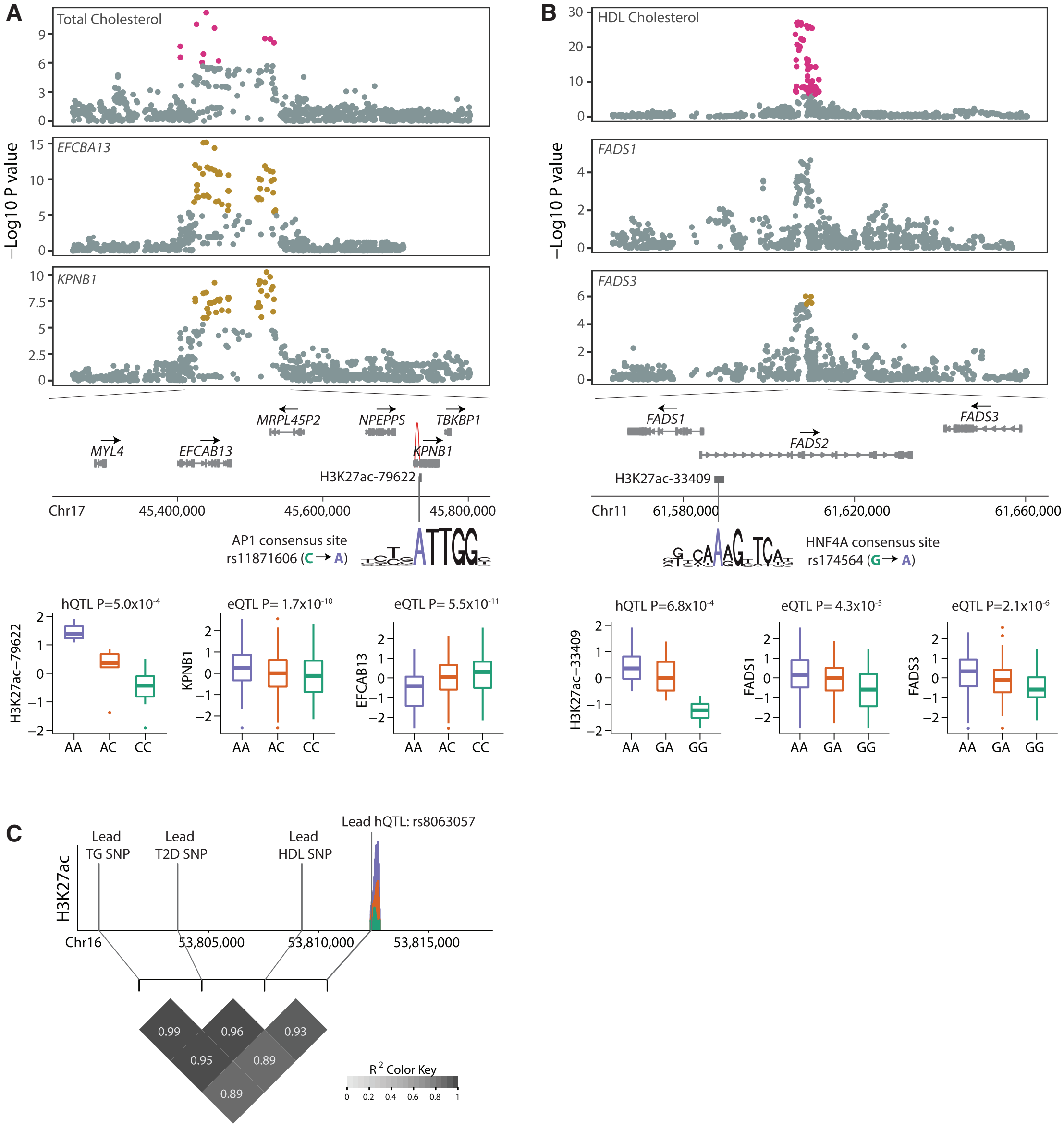

Figure 6. Patterns of eQTL, hQTL, Capture-C Signals in Fine-Mapped GWAS Loci

(A) Significant colocalization signals at the 17q21.32 locus are displayed using Manhattan plots. Colocalization posterior probability of total cholesterol GWAS associations with *EFCAB13* gene expression was 0.999 and with *KPNB1* was 0.934. Schematic representation of the genes in the zoomed-in locus of chr17: 45,250,000–45,800,000 and the putatively trait-relevant H3K27ac-79622 peak (chr17: 45,733,609–45,733,977). H3K27ac-79622 peak only showed significant DNA looping interaction with the promoter of *KPNB1* in the genome (CHiCAGO score: 7.26). The A allele at rs11871606 is the lead hQTL of this peak and significantly increases the odds of AP1 binding ($p = 4.89 \times 10^{-4}$).[85] Boxplots of normalized H3K27ac-79622 ChIP-seq, *KPNB1*, and *EFCAB13* RNA-seq read counts stratified by genotype at the rs11871606. Sample sizes of each genotype group were AA:3, AC:5, CC:10 for ChIP-seq data and AA:49, AC:111, CC:81 for *KPNB1* and *EFCAB13* RNA-seq data.

(B) Significant colocalization signals at the chromosome 11q12.2 locus. Colocalization posterior probabilities of HDL cholesterol associations with *FADS1* and *FADS3* gene expression levels were 0.81 and 0.94, respectively. Schematic representation of the genes in the zoomed-in locus of chr11: 61,560,452–61,654,826 and the putatively trait-relevant H3K27ac-33409 peak (chr11: 61,587,373–61,589,527). An allele of the candidate causal variant, rs174564, increases the odds of HNF4A binding ($p = 3.4 \times 10^{-5}$).[85] Boxplots of normalized H3K27ac-33409 ChIP-seq, *FADS1* and *FADS3* RNA-seq read counts stratified by genotype at the rs174564. Sample sizes of each genotype group were AA:10, GA:4, GG:4 for ChIP-seq data and AA:112, GA:96, GG:31 for *FADS1* and *FADS3* RNA-seq data.

(C) 16q12.2 GWAS locus where putatively trait-relevant regulatory element was identified in the first intron of the *FTO* gene (H3K27ac-70404; chr16: 53,812,377–53,812,817). Sushi plot shows the mean normalized H3K27ac-70404 ChIP-seq read counts of each genotype group at rs8063057. This peak could not be assigned to any gene in the human liver tissue. LD heatmap shows the r^2 between lead hQTL and the lead GWAS variants of T2 diabetes, HDL, and triglyceride levels.

(MIM: 610966) (H3K27ac-70404; chr16: 53,812,377–53,812,817) with evidence of underlying GWAS associations in this locus with type 2 diabetes, HDL, and triglyceride levels (Figure 6D). To our knowledge, there has not been a previous report of a genotype-dependent regulatory element in the human liver that overlaps the

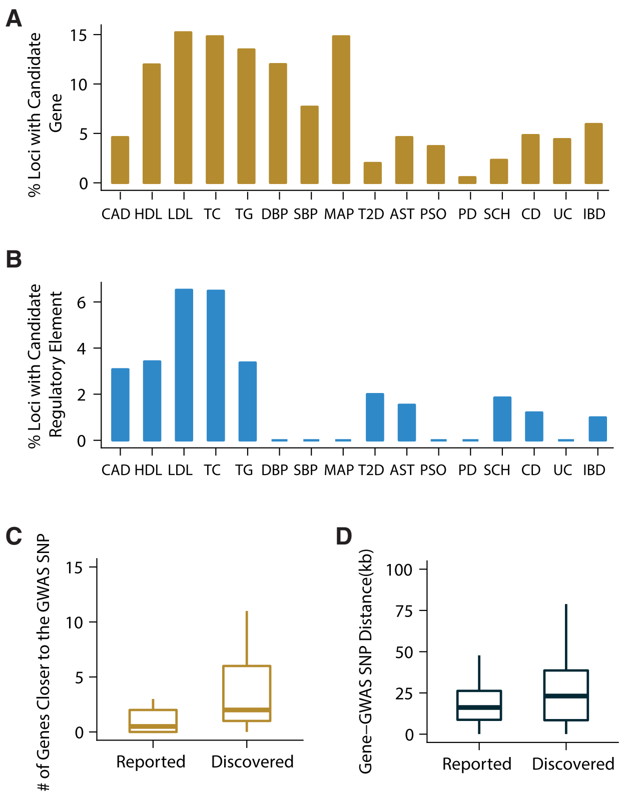

Figure 7. Characteristics of Fine-Mapped GWAS Loci
(A) Percentages of GWAS loci with at least one significantly colocalized gene are shown for each of the complex phenotypes.
(B) Percentages of GWAS loci with at least one candidate regulatory element are shown for each of the complex phenotypes.
(C) Number of genes that are closer to the lead GWAS variant are shown for genes that were previously suggested as trait relevant (i.e., genes shown in Figure 5C) and for genes that were discovered as trait relevant in our study (i.e., genes shown in Figure 5E).
(D) Distance between lead GWAS variant and the TSS of the candidate trait-relevant gene identified. "Reported" corresponds to genes that were previously reported as likely trait relevant (i.e., genes shown in Figure 5C) and "Discovered" corresponds to the trait-relevant genes that have not been previously suggested to underlie the corresponding GWAS phenotype (i.e., genes shown in Figure 5E).

GWAS interval at this locus, which has received considerable study.[89]

Characteristics of Fine-Mapped GWAS Loci

Overall, using the genome-wide data collected in the human liver, we were able to fine-map at least one GWAS locus for 16 out of 20 phenotypes we studied (Figure 5). 30% of the trait-relevant genes had liver-specific *cis*-eQTLs, 16% had shared *cis*-eQTLs, and the remaining 54% had *cis*-eQTLs that were neither classified as liver-specific nor as shared. When we looked at the percentage of GWAS loci with at least one candidate gene (Figure 7A) or regulatory element (Figure 7B) identified in our study, we found that our ability to identify trait-relevant genes and regulatory elements in GWAS loci is correlated with the physiological relevance of the studied phenotype to the human liver. Phenotypes with a known molecular basis in the liver such as blood lipid phenotypes had larger proportion of GWAS loci with candidate genes or regulatory elements identified in our study (Figures 7A and 7B).

A median of two genes were located closer to the lead GWAS variant than the trait-relevant gene identified in this study. When we compared the candidate trait-relevant genes discovered in our study (Figure 5E) to genes that were previously suggested to be trait relevant in the literature (Figure 5C), we noted a significant difference both in terms of the distance and the number of genes between the lead GWAS variants and the trait-relevant genes identified (Figures 7C and 7D). This discrepancy emphasizes once again that genes reported as trait relevant in the literature are biased toward those that are closer to the GWAS lead variants and that unbiased genome-wide approaches are required to identify true trait-relevant genes in GWAS loci.

We also note that mutations in four of the complex phenotype-causing genes, *LIPA* (MIM: 613497), *LIPC* (MIM: 151670), *GPIHBP1* (MIM: 612757), and *IRF8* (MIM: 601565), have been implicated in related Mendelian diseases,[90–93] and 50% of the trait-relevant genes that have murine models were reported to display similar phenotype in the model organism as well (Mouse Genome Database; Table S11). Lastly, while *LIPA*, *PLTP* (MIM: 172425), and *SLC39A8* (MIM: 608732) have been previously suggested to affect their associated phenotypes through protein altering mutations,[94–96] our findings are in line with those of Wild et al.[97] and Hess et al.,[98] suggesting that genotype-dependent changes in their gene expression levels also contribute to the complex trait pathogenesis.

Discussion

In 2001, the first published draft of the human genome confirmed that the vast majority of its sequence, approximately 97% of the 3.2 billion bases, has no protein-coding function.[99] Following this discovery, the next phase of research focused on understanding and functionally annotating non-coding regions within the human genome. These studies generated reference epigenomic maps for multiple cell lines and tissue types and demonstrated that epigenetic marks on histone proteins are important predictors of gene-regulatory activity.[100] Perhaps more interestingly, such gene regulatory regions were subsequently shown to harbor the majority of the complex disease-associated variants,[4] making studies of gene regulation an important area of investigation at the interface of basic and disease biology.

In this study, we generated the most comprehensive, in terms of sample size and characterizing inter-individual differences, genome-wide dataset of two epigenetic marks, H3K4me3 and H3K27ac, in the human liver. Using DNA-looping interactions, we identified at least one target interacting regulatory element for 65.4% of the genes that were baited and detected as expressed. We demonstrated

widespread functional consequences of natural genetic variation on regulatory element activity and gene expression levels. Furthermore, we showed that a single genetic variant could co-regulate both histone modification states and gene expression levels and this co-regulation is at least partly mediated via DNA looping interactions. We expect that this expansive resource containing functional annotation of non-coding elements and DNA-looping interactions between gene promoters and putative functional gene regulatory elements will greatly facilitate future analyses and stimulate new areas of investigation.

Our results also hold significant relevance for medical genomics. Using genetic colocalization approaches, we fine-mapped a total of 74 GWAS loci associated with at least one complex phenotype. For 21 loci, the gene we prioritized had been previously reported as the likely trait-relevant gene in the majority of the literature. For 10 loci, our findings helped refine identification of the candidate gene from among several genes that were suggested to be trait relevant in the literature. In 21 loci, we discovered candidate trait-relevant genes that have not been previously suggested to underlie the corresponding GWAS phenotype and for a total of 16 loci, we identified candidate trait-relevant gene regulatory elements.

While our efforts constitute the largest GWAS fine-mapping effort performed in the human liver, we were able to identify candidate trait-relevant genes in less than 20% of the GWAS loci even for the most directly liver-related complex phenotypes (i.e., blood lipid levels). This result indicates a need for similar comprehensive studies of the transcriptome and epigenome in a wider range of tissue types and stimulation conditions as well as studies focusing on other complex disease-causing mechanisms. Additionally, we believe performing Capture-C experiments in the human liver tissue as opposed to immortalized HepG2 cell lines could increase our fine-mapping power. We note that while HepG2 cell lines are a widely accepted model system to study liver biology, they display an abnormal hyperdiploid karyotype, which could have effects on chromatin-chromatin interactions. It is also likely that there is inter-individual variation in chromatin-chromatin interactions and when possible Capture-C data should be obtained across several individuals as opposed to a single cell line. Our eQTL and hQTL mapping experiments were performed in whole liver tissue samples and performing similar studies in isolated liver cell types or single cells could also enhance GWAS fine-mapping ability. Furthermore, while statistical colocalization approaches help prioritization of genes in GWAS loci, it is possible that the application of such approaches to eQTL and GWAS summary statistics from larger cohorts may reveal additional colocalization signals in these same GWAS loci. Lastly, while we recognize and value the contribution of genome-wide integrative approaches we and others[16–22] have undertaken, we note that further *in vivo* and organism-level validations are necessary to confirm the suggested causality of these findings.

Overall, our findings expand the repertoire of candidate genes and regulatory mechanisms governing complex disease development and contribute to basic understanding of genetic and epigenetic regulation of gene expression in the human liver tissue. Furthermore, by more precisely highlighting genes and regulatory elements with relevance to disease or critical intermediate phenotypes, we believe this study will improve research into the development of therapeutic or preventative measures to mitigate the effects of complex disease. Finally, our approaches to integrate genetic variation and multiple molecular phenotypes across individuals are likely to be applicable to other tissues and traits.

Accession Numbers

RNA-seq and ChIP-seq datasets from Penn Cohorts have been deposited in GEO under accession number of GSE128072. Genotype and RNA-seq datasets from GTEx Cohort have been deposited in the dbGaP under ID code of phs000424.v6.p1. HepG2 Capture-C data have been deposited in ArrayExpress with accession number E-MTAB-7144.

Supplemental Data

Supplemental Data can be found online at https://doi.org/10.1016/j.ajhg.2019.05.010.

Acknowledgments

We thank all liver donors and their families for participating in this study. We thank Ana Gonzalez and Mary Shaw of Penn Transplant Institute for coordinating donor tissue and donor information sharing. We thank Daniel Savic for his input on ChIP-seq experiments, Jonathan Schug and Susannah Elwyn for their help with generating RNA-seq and ChIP-seq data, Arif Harmanci for his input in ChIP-seq peak calling, Graham McVicker for his input on generating read density plots, and Ben Strober for sharing *trans*-eQTL cross-mapping regions list. We thank Greg Cooper, Barbara Engelhardt, and Katalin Susztak for helpful discussions and comments on an earlier draft of the manuscript. This study was supported by NIH grants R01 HL133218 (C.D.B.), R01 AG057516 (S.F.A.G.), HL055323 (A.M.B., D.J.R.), HL134853 (N.J.H., D.J.R.), HL109489 (D.J.R.), and HG006398 (D.J.R.). The Genotype-Tissue Expression (GTEx) Project was supported by the Common Fund of the Office of the Director of the National Institutes of Health, and by NCI, NHGRI, NHLBI, NIDA, NIMH, and NINDS. M.Ç. was partially supported by Penn IBI Postdoctoral Collaboration Fellowship. S.F.A.G. is supported by the Daniel B. Burke Endowed Chair for Diabetes Research.

Declaration of Interests

The authors declare no competing interests.

Received: October 5, 2018
Accepted: May 13, 2019
Published: June 13, 2019

Web Resources

ArrayExpress, https://www.ebi.ac.uk/arrayexpress/

dbGaP, https://www.ncbi.nlm.nih.gov/gap
GEO, https://www.ncbi.nlm.nih.gov/geo/
Mouse Genome Informatics, http://www.informatics.jax.org/
OMIM, https://www.omim.org/
Trim Galore!, http://www.bioinformatics.babraham.ac.uk/projects/trim_galore/

References

1. Wang, F.S., Fan, J.G., Zhang, Z., Gao, B., and Wang, H.Y. (2014). The global burden of liver disease: the major impact of China. Hepatology *60*, 2099–2108.
2. Ongen, H., Brown, A.A., Delaneau, O., Panousis, N.I., Nica, A.C., Dermitzakis, E.T.; and GTEx Consortium (2017). Estimating the causal tissues for complex traits and diseases. Nat. Genet. *49*, 1676–1683.
3. Stranger, B.E., Stahl, E.A., and Raj, T. (2011). Progress and promise of genome-wide association studies for human complex trait genetics. Genetics *187*, 367–383.
4. Maurano, M.T., Humbert, R., Rynes, E., Thurman, R.E., Haugen, E., Wang, H., Reynolds, A.P., Sandstrom, R., Qu, H., Brody, J., et al. (2012). Systematic localization of common disease-associated variation in regulatory DNA. Science *337*, 1190–1195.
5. ENCODE Project Consortium (2012). An integrated encyclopedia of DNA elements in the human genome. Nature *489*, 57–74.
6. Bernstein, B.E., Stamatoyannopoulos, J.A., Costello, J.F., Ren, B., Milosavljevic, A., Meissner, A., Kellis, M., Marra, M.A., Beaudet, A.L., Ecker, J.R., et al. (2010). The NIH Roadmap Epigenomics Mapping Consortium. Nat. Biotechnol. *28*, 1045–1048.
7. Pomerantz, M.M., Ahmadiyeh, N., Jia, L., Herman, P., Verzi, M.P., Doddapaneni, H., Beckwith, C.A., Chan, J.A., Hills, A., Davis, M., et al. (2009). The 8q24 cancer risk variant rs6983267 shows long-range interaction with MYC in colorectal cancer. Nat. Genet. *41*, 882–884.
8. Wasserman, N.F., Aneas, I., and Nobrega, M.A. (2010). An 8q24 gene desert variant associated with prostate cancer risk confers differential in vivo activity to a MYC enhancer. Genome Res. *20*, 1191–1197.
9. McCarroll, S.A., Huett, A., Kuballa, P., Chilewski, S.D., Landry, A., Goyette, P., Zody, M.C., Hall, J.L., Brant, S.R., Cho, J.H., et al. (2008). Deletion polymorphism upstream of IRGM associated with altered IRGM expression and Crohn's disease. Nat. Genet. *40*, 1107–1112.
10. Bauer, D.E., Kamran, S.C., Lessard, S., Xu, J., Fujiwara, Y., Lin, C., Shao, Z., Canver, M.C., Smith, E.C., Pinello, L., et al. (2013). An erythroid enhancer of BCL11A subject to genetic variation determines fetal hemoglobin level. Science *342*, 253–257.
11. Musunuru, K., Strong, A., Frank-Kamenetsky, M., Lee, N.E., Ahfeldt, T., Sachs, K.V., Li, X., Li, H., Kuperwasser, N., Ruda, V.M., et al. (2010). From noncoding variant to phenotype via SORT1 at the 1p13 cholesterol locus. Nature *466*, 714–719.
12. Zhang, X., Cowper-Sal lari, R., Bailey, S.D., Moore, J.H., and Lupien, M. (2012). Integrative functional genomics identifies an enhancer looping to the SOX9 gene disrupted by the 17q24.3 prostate cancer risk locus. Genome Res. *22*, 1437–1446.
13. Smemo, S., Tena, J.J., Kim, K.H., Gamazon, E.R., Sakabe, N.J., Gómez-Marín, C., Aneas, I., Credidio, F.L., Sobreira, D.R., Wasserman, N.F., et al. (2014). Obesity-associated variants within FTO form long-range functional connections with IRX3. Nature *507*, 371–375.
14. Huang, Q., Whitington, T., Gao, P., Lindberg, J.F., Yang, Y., Sun, J., Väisänen, M.R., Szulkin, R., Annala, M., Yan, J., et al. (2014). A prostate cancer susceptibility allele at 6q22 increases RFX6 expression by modulating HOXB13 chromatin binding. Nat. Genet. *46*, 126–135.
15. Savic, D., Gertz, J., Jain, P., Cooper, G.M., and Myers, R.M. (2013). Mapping genome-wide transcription factor binding sites in frozen tissues. Epigenetics Chromatin *6*, 30.
16. Alasoo, K., Rodrigues, J., Mukhopadhyay, S., Knights, A.J., Mann, A.L., Kundu, K., Hale, C., Dougan, G., Gaffney, D.J.; and HIPSCI Consortium (2018). Shared genetic effects on chromatin and gene expression indicate a role for enhancer priming in immune response. Nat. Genet. *50*, 424–431.
17. Degner, J.F., Pai, A.A., Pique-Regi, R., Veyrieras, J.B., Gaffney, D.J., Pickrell, J.K., De Leon, S., Michelini, K., Lewellen, N., Crawford, G.E., et al. (2012). DNaseI sensitivity QTLs are a major determinant of human expression variation. Nature *482*, 390–394.
18. Grubert, F., Zaugg, J.B., Kasowski, M., Ursu, O., Spacek, D.V., Martin, A.R., Greenside, P., Srivas, R., Phanstiel, D.H., Pekowska, A., et al. (2015). Genetic Control of Chromatin States in Humans Involves Local and Distal Chromosomal Interactions. Cell *162*, 1051–1065.
19. Kasowski, M., Kyriazopoulou-Panagiotopoulou, S., Grubert, F., Zaugg, J.B., Kundaje, A., Liu, Y., Boyle, A.P., Zhang, Q.C., Zakharia, F., Spacek, D.V., et al. (2013). Extensive variation in chromatin states across humans. Science *342*, 750–752.
20. Kumasaka, N., Knights, A.J., and Gaffney, D.J. (2016). Fine-mapping cellular QTLs with RASQUAL and ATAC-seq. Nat. Genet. *48*, 206–213.
21. McVicker, G., van de Geijn, B., Degner, J.F., Cain, C.E., Banovich, N.E., Raj, A., Lewellen, N., Myrthil, M., Gilad, Y., and Pritchard, J.K. (2013). Identification of genetic variants that affect histone modifications in human cells. Science *342*, 747–749.
22. Waszak, S.M., Delaneau, O., Gschwind, A.R., Kilpinen, H., Raghav, S.K., Witwicki, R.M., Orioli, A., Wiederkehr, M., Panousis, N.I., Yurovsky, A., et al. (2015). Population Variation and Genetic Control of Modular Chromatin Architecture in Humans. Cell *162*, 1039–1050.
23. Carithers, L.J., Ardlie, K., Barcus, M., Branton, P.A., Britton, A., Buia, S.A., Compton, C.C., DeLuca, D.S., Peter-Demchok, J., Gelfand, E.T., et al.; GTEx Consortium (2015). A Novel Approach to High-Quality Postmortem Tissue Procurement: The GTEx Project. Biopreserv. Biobank. *13*, 311–319.
24. GTEx Consortium (2013). The Genotype-Tissue Expression (GTEx) project. Nat. Genet. *45*, 580–585.
25. Battle, A., Brown, C.D., Engelhardt, B.E., Montgomery, S.B.; GTEx Consortium; Laboratory, Data Analysis &Coordinating Center (LDACC)—Analysis Working Group; Statistical Methods groups—Analysis Working Group; Enhancing GTEx (eGTEx) groups; NIH Common Fund; NIH/NCI; NIH/NHGRI; NIH/NIMH; NIH/NIDA; Biospecimen Collection Source Site—NDRI; Biospecimen Collection Source Site—RPCI; Biospecimen Core Resource—VARI; Brain Bank Repository—University of Miami Brain Endowment Bank; Leidos Biomedical—Project Management; ELSI Study; Genome Browser Data Integration &Visualization—EBI; Genome Browser Data Integration &Visualization—UCSC Genomics

Institute, University of California Santa Cruz; Lead analysts; Laboratory, Data Analysis &Coordinating Center (LDACC); NIH program management; Biospecimen collection; Pathology; and eQTL manuscript working group (2017). Genetic effects on gene expression across human tissues. Nature *550*, 204–213.
26. GTEx Consortium (2015). Human genomics. The Genotype-Tissue Expression (GTEx) pilot analysis: multitissue gene regulation in humans. Science *348*, 648–660.
27. Hughes, J.R., Roberts, N., McGowan, S., Hay, D., Giannoulatou, E., Lynch, M., De Gobbi, M., Taylor, S., Gibbons, R., and Higgs, D.R. (2014). Analysis of hundreds of cis-regulatory landscapes at high resolution in a single, high-throughput experiment. Nat. Genet. *46*, 205–212.
28. Li, H. (2013). Aligning sequence reads, clone sequences and assembly contigs with BWA-MEM. arXiv., 1303.3997.
29. Li, H., Handsaker, B., Wysoker, A., Fennell, T., Ruan, J., Homer, N., Marth, G., Abecasis, G., Durbin, R.; and 1000 Genome Project Data Processing Subgroup (2009). The Sequence Alignment/Map format and SAMtools. Bioinformatics *25*, 2078–2079.
30. Zhang, Y., Liu, T., Meyer, C.A., Eeckhoute, J., Johnson, D.S., Bernstein, B.E., Nusbaum, C., Myers, R.M., Brown, M., Li, W., and Liu, X.S. (2008). Model-based analysis of ChIP-Seq (MACS). Genome Biol. *9*, R137.
31. Landt, S.G., Marinov, G.K., Kundaje, A., Kheradpour, P., Pauli, F., Batzoglou, S., Bernstein, B.E., Bickel, P., Brown, J.B., Cayting, P., et al. (2012). ChIP-seq guidelines and practices of the ENCODE and modENCODE consortia. Genome Res. *22*, 1813–1831.
32. Ramírez, F., Ryan, D.P., Grüning, B., Bhardwaj, V., Kilpert, F., Richter, A.S., Heyne, S., Dündar, F., and Manke, T. (2016). deepTools2: a next generation web server for deep-sequencing data analysis. Nucleic Acids Res. *44* (W1), W160-5.
33. Lawrence, M., Huber, W., Pagès, H., Aboyoun, P., Carlson, M., Gentleman, R., Morgan, M.T., and Carey, V.J. (2013). Software for computing and annotating genomic ranges. PLoS Comput. Biol. *9*, e1003118.
34. Dobin, A., Davis, C.A., Schlesinger, F., Drenkow, J., Zaleski, C., Jha, S., Batut, P., Chaisson, M., and Gingeras, T.R. (2013). STAR: ultrafast universal RNA-seq aligner. Bioinformatics *29*, 15–21.
35. Li, B., and Dewey, C.N. (2011). RSEM: accurate transcript quantification from RNA-Seq data with or without a reference genome. BMC Bioinformatics *12*, 323.
36. Purcell, S., Neale, B., Todd-Brown, K., Thomas, L., Ferreira, M.A., Bender, D., Maller, J., Sklar, P., de Bakker, P.I., Daly, M.J., and Sham, P.C. (2007). PLINK: a tool set for whole-genome association and population-based linkage analyses. Am. J. Hum. Genet. *81*, 559–575.
37. Delaneau, O., Marchini, J., and Zagury, J.F. (2011). A linear complexity phasing method for thousands of genomes. Nat. Methods *9*, 179–181.
38. Howie, B., Fuchsberger, C., Stephens, M., Marchini, J., and Abecasis, G.R. (2012). Fast and accurate genotype imputation in genome-wide association studies through pre-phasing. Nat. Genet. *44*, 955–959.
39. Auton, A., Brooks, L.D., Durbin, R.M., Garrison, E.P., Kang, H.M., Korbel, J.O., Marchini, J.L., McCarthy, S., McVean, G.A., Abecasis, G.R.; and 1000 Genomes Project Consortium (2015). A global reference for human genetic variation. Nature *526*, 68–74.
40. Wingett, S., Ewels, P., Furlan-Magaril, M., Nagano, T., Schoenfelder, S., Fraser, P., and Andrews, S. (2015). HiCUP: pipeline for mapping and processing Hi-C data. F1000Res. *4*, 1310.
41. Cairns, J., Freire-Pritchett, P., Wingett, S.W., Várnai, C., Dimond, A., Plagnol, V., Zerbino, D., Schoenfelder, S., Javierre, B.M., Osborne, C., et al. (2016). CHiCAGO: robust detection of DNA looping interactions in Capture Hi-C data. Genome Biol. *17*, 127.
42. Chesi, A., Wagley, Y., Johnson, M.E., Manduchi, E., Su, C., Lu, S., Leonard, M.E., Hodge, K.M., Pippin, J.A., Hankenson, K.D., et al. (2019). Genome-scale Capture C promoter interactions implicate effector genes at GWAS loci for bone mineral density. Nat. Commun. *10*, 1260.
43. Price, A.L., Patterson, N.J., Plenge, R.M., Weinblatt, M.E., Shadick, N.A., and Reich, D. (2006). Principal components analysis corrects for stratification in genome-wide association studies. Nat. Genet. *38*, 904–909.
44. Altshuler, D.M., Gibbs, R.A., Peltonen, L., Altshuler, D.M., Gibbs, R.A., Peltonen, L., Dermitzakis, E., Schaffner, S.F., Yu, F., Peltonen, L., et al.; International HapMap 3 Consortium (2010). Integrating common and rare genetic variation in diverse human populations. Nature *467*, 52–58.
45. Ongen, H., Buil, A., Brown, A.A., Dermitzakis, E.T., and Delaneau, O. (2016). Fast and efficient QTL mapper for thousands of molecular phenotypes. Bioinformatics *32*, 1479–1485.
46. Stegle, O., Parts, L., Piipari, M., Winn, J., and Durbin, R. (2012). Using probabilistic estimation of expression residuals (PEER) to obtain increased power and interpretability of gene expression analyses. Nat. Protoc. *7*, 500–507.
47. Willer, C.J., Li, Y., and Abecasis, G.R. (2010). METAL: fast and efficient meta-analysis of genomewide association scans. Bioinformatics *26*, 2190–2191.
48. Han, B., and Eskin, E. (2012). Interpreting meta-analyses of genome-wide association studies. PLoS Genet. *8*, e1002555.
49. Pers, T.H., Timshel, P., and Hirschhorn, J.N. (2015). SNPsnap: a Web-based tool for identification and annotation of matched SNPs. Bioinformatics *31*, 418–420.
50. Phipson, B., and Smyth, G.K. (2010). Permutation P-values should never be zero: calculating exact P-values when permutations are randomly drawn. Stat. Appl. Genet. Mol. Biol. *9*, e39.
51. Shabalin, A.A. (2012). Matrix eQTL: ultra fast eQTL analysis via large matrix operations. Bioinformatics *28*, 1353–1358.
52. Ravasi, T., Suzuki, H., Cannistraci, C.V., Katayama, S., Bajic, V.B., Tan, K., Akalin, A., Schmeier, S., Kanamori-Katayama, M., Bertin, N., et al. (2010). An atlas of combinatorial transcriptional regulation in mouse and man. Cell *140*, 744–752.
53. McKenna, A., Hanna, M., Banks, E., Sivachenko, A., Cibulskis, K., Kernytsky, A., Garimella, K., Altshuler, D., Gabriel, S., Daly, M., and DePristo, M.A. (2010). The Genome Analysis Toolkit: a MapReduce framework for analyzing next-generation DNA sequencing data. Genome Res. *20*, 1297–1303.
54. Nikpay, M., Goel, A., Won, H.H., Hall, L.M., Willenborg, C., Kanoni, S., Saleheen, D., Kyriakou, T., Nelson, C.P., Hopewell, J.C., et al. (2015). A comprehensive 1,000 Genomes-based genome-wide association meta-analysis of coronary artery disease. Nat. Genet. *47*, 1121–1130.
55. Willer, C.J., Schmidt, E.M., Sengupta, S., Peloso, G.M., Gustafsson, S., Kanoni, S., Ganna, A., Chen, J., Buchkovich, M.L., Mora, S., et al.; Global Lipids Genetics Consortium (2013). Discovery and refinement of loci associated with lipid levels. Nat. Genet. *45*, 1274–1283.

56. Ehret, G.B., Munroe, P.B., Rice, K.M., Bochud, M., Johnson, A.D., Chasman, D.I., Smith, A.V., Tobin, M.D., Verwoert, G.C., Hwang, S.J., et al.; International Consortium for Blood Pressure Genome-Wide Association Studies; CARDIoGRAM consortium; CKDGen Consortium; KidneyGen Consortium; EchoGen consortium; and CHARGE-HF consortium (2011). Genetic variants in novel pathways influence blood pressure and cardiovascular disease risk. Nature *478*, 103–109.
57. Stahl, E.A., Raychaudhuri, S., Remmers, E.F., Xie, G., Eyre, S., Thomson, B.P., Li, Y., Kurreeman, F.A., Zhernakova, A., Hinks, A., et al.; BIRAC Consortium; and YEAR Consortium (2010). Genome-wide association study meta-analysis identifies seven new rheumatoid arthritis risk loci. Nat. Genet. *42*, 508–514.
58. Mahajan, A., Go, M.J., Zhang, W., Below, J.E., Gaulton, K.J., Ferreira, T., Horikoshi, M., Johnson, A.D., Ng, M.C., Prokopenko, I., et al.; DIAbetes Genetics Replication And Meta-analysis (DIAGRAM) Consortium; Asian Genetic Epidemiology Network Type 2 Diabetes (AGEN-T2D) Consortium; South Asian Type 2 Diabetes (SAT2D) Consortium; Mexican American Type 2 Diabetes (MAT2D) Consortium; and Type 2 Diabetes Genetic Exploration by Nex-generation sequencing in muylti-Ethnic Samples (T2D-GENES) Consortium (2014). Genome-wide trans-ancestry meta-analysis provides insight into the genetic architecture of type 2 diabetes susceptibility. Nat. Genet. *46*, 234–244.
59. Sudlow, C., Gallacher, J., Allen, N., Beral, V., Burton, P., Danesh, J., Downey, P., Elliott, P., Green, J., Landray, M., et al. (2015). UK biobank: an open access resource for identifying the causes of a wide range of complex diseases of middle and old age. PLoS Med. *12*, e1001779.
60. Lambert, J.C., Ibrahim-Verbaas, C.A., Harold, D., Naj, A.C., Sims, R., Bellenguez, C., DeStafano, A.L., Bis, J.C., Beecham, G.W., Grenier-Boley, B., et al.; European Alzheimer's Disease Initiative (EADI); Genetic and Environmental Risk in Alzheimer's Disease; Alzheimer's Disease Genetic Consortium; and Cohorts for Heart and Aging Research in Genomic Epidemiology (2013). Meta-analysis of 74,046 individuals identifies 11 new susceptibility loci for Alzheimer's disease. Nat. Genet. *45*, 1452–1458.
61. Schizophrenia Working Group of the Psychiatric Genomics Consortium (2014). Biological insights from 108 schizophrenia-associated genetic loci. Nature *511*, 421–427.
62. Liu, J.Z., van Sommeren, S., Huang, H., Ng, S.C., Alberts, R., Takahashi, A., Ripke, S., Lee, J.C., Jostins, L., Shah, T., et al.; International Multiple Sclerosis Genetics Consortium; and International IBD Genetics Consortium (2015). Association analyses identify 38 susceptibility loci for inflammatory bowel disease and highlight shared genetic risk across populations. Nat. Genet. *47*, 979–986.
63. Fritsche, L.G., Loenhardt, T., Janssen, A., Fisher, S.A., Rivera, A., Keilhauer, C.N., and Weber, B.H. (2008). Age-related macular degeneration is associated with an unstable ARMS2 (LOC387715) mRNA. Nat. Genet. *40*, 892–896.
64. Giambartolomei, C., Vukcevic, D., Schadt, E.E., Franke, L., Hingorani, A.D., Wallace, C., and Plagnol, V. (2014). Bayesian test for colocalisation between pairs of genetic association studies using summary statistics. PLoS Genet. *10*, e1004383.
65. Creyghton, M.P., Cheng, A.W., Welstead, G.G., Kooistra, T., Carey, B.W., Steine, E.J., Hanna, J., Lodato, M.A., Frampton, G.M., Sharp, P.A., et al. (2010). Histone H3K27ac separates active from poised enhancers and predicts developmental state. Proc. Natl. Acad. Sci. USA *107*, 21931–21936.
66. Karlić, R., Chung, H.R., Lasserre, J., Vlahovicek, K., and Vingron, M. (2010). Histone modification levels are predictive for gene expression. Proc. Natl. Acad. Sci. USA *107*, 2926–2931.
67. Liang, G., Lin, J.C., Wei, V., Yoo, C., Cheng, J.C., Nguyen, C.T., Weisenberger, D.J., Egger, G., Takai, D., Gonzales, F.A., and Jones, P.A. (2004). Distinct localization of histone H3 acetylation and H3-K4 methylation to the transcription start sites in the human genome. Proc. Natl. Acad. Sci. USA *101*, 7357–7362.
68. Das, P.M., Ramachandran, K., vanWert, J., and Singal, R. (2004). Chromatin immunoprecipitation assay. Biotechniques *37*, 961–969.
69. Andersson, R., Gebhard, C., Miguel-Escalada, I., Hoof, I., Bornholdt, J., Boyd, M., Chen, Y., Zhao, X., Schmidl, C., Suzuki, T., et al. (2014). An atlas of active enhancers across human cell types and tissues. Nature *507*, 455–461.
70. Phanstiel, D.H., Boyle, A.P., Araya, C.L., and Snyder, M.P. (2014). Sushi.R: flexible, quantitative and integrative genomic visualizations for publication-quality multi-panel figures. Bioinformatics *30*, 2808–2810.
71. Yin, T., Cook, D., and Lawrence, M. (2012). ggbio: an R package for extending the grammar of graphics for genomic data. Genome Biol. *13*, R77.
72. Anderson, C.A., Boucher, G., Lees, C.W., Franke, A., D'Amato, M., Taylor, K.D., Lee, J.C., Goyette, P., Imielinski, M., Latiano, A., et al. (2011). Meta-analysis identifies 29 additional ulcerative colitis risk loci, increasing the number of confirmed associations to 47. Nat. Genet. *43*, 246–252.
73. de Lange, K.M., Moutsianas, L., Lee, J.C., Lamb, C.A., Luo, Y., Kennedy, N.A., Jostins, L., Rice, D.L., Gutierrez-Achury, J., Ji, S.G., et al. (2017). Genome-wide association study implicates immune activation of multiple integrin genes in inflammatory bowel disease. Nat. Genet. *49*, 256–261.
74. Kathiresan, S., Willer, C.J., Peloso, G.M., Demissie, S., Musunuru, K., Schadt, E.E., Kaplan, L., Bennett, D., Li, Y., Tanaka, T., et al. (2009). Common variants at 30 loci contribute to polygenic dyslipidemia. Nat. Genet. *41*, 56–65.
75. Pickrell, J.K., Berisa, T., Liu, J.Z., Ségurel, L., Tung, J.Y., and Hinds, D.A. (2016). Detection and interpretation of shared genetic influences on 42 human traits. Nat. Genet. *48*, 709–717.
76. Spracklen, C.N., Chen, P., Kim, Y.J., Wang, X., Cai, H., Li, S., Long, J., Wu, Y., Wang, Y.X., Takeuchi, F., et al. (2017). Association analyses of East Asian individuals and trans-ancestry analyses with European individuals reveal new loci associated with cholesterol and triglyceride levels. Hum. Mol. Genet. *26*, 1770–1784.
77. Surakka, I., Horikoshi, M., Mägi, R., Sarin, A.P., Mahajan, A., Lagou, V., Marullo, L., Ferreira, T., Miraglio, B., Timonen, S., et al.; ENGAGE Consortium (2015). The impact of low-frequency and rare variants on lipid levels. Nat. Genet. *47*, 589–597.
78. Teslovich, T.M., Musunuru, K., Smith, A.V., Edmondson, A.C., Stylianou, I.M., Koseki, M., Pirruccello, J.P., Ripatti, S., Chasman, D.I., Willer, C.J., et al. (2010). Biological, clinical and population relevance of 95 loci for blood lipids. Nature *466*, 707–713.
79. van der Harst, P., and Verweij, N. (2018). Identification of 64 Novel Genetic Loci Provides an Expanded View on the Genetic Architecture of Coronary Artery Disease. Circ. Res. *122*, 433–443.

80. Fritsche, L.G., Igl, W., Bailey, J.N., Grassmann, F., Sengupta, S., Bragg-Gresham, J.L., Burdon, K.P., Hebbring, S.J., Wen, C., Gorski, M., et al. (2016). A large genome-wide association study of age-related macular degeneration highlights contributions of rare and common variants. Nat. Genet. *48*, 134–143.
81. Huang, H., Fang, M., Jostins, L., Umićević Mirkov, M., Boucher, G., Anderson, C.A., Andersen, V., Cleynen, I., Cortes, A., Crins, F., et al.; International Inflammatory Bowel Disease Genetics Consortium (2017). Fine-mapping inflammatory bowel disease loci to single-variant resolution. Nature *547*, 173–178.
82. Jostins, L., Ripke, S., Weersma, R.K., Duerr, R.H., McGovern, D.P., Hui, K.Y., Lee, J.C., Schumm, L.P., Sharma, Y., Anderson, C.A., et al.; International IBD Genetics Consortium (IIBDGC) (2012). Host-microbe interactions have shaped the genetic architecture of inflammatory bowel disease. Nature *491*, 119–124.
83. Li, Z., Chen, J., Yu, H., He, L., Xu, Y., Zhang, D., Yi, Q., Li, C., Li, X., Shen, J., et al. (2017). Genome-wide association analysis identifies 30 new susceptibility loci for schizophrenia. Nat. Genet. *49*, 1576–1583.
84. van Leeuwen, E.M., Sabo, A., Bis, J.C., Huffman, J.E., Manichaikul, A., Smith, A.V., Feitosa, M.F., Demissie, S., Joshi, P.K., Duan, Q., et al.; LifeLines Cohort Study; and CHARGE Lipids Working Group (2016). Meta-analysis of 49 549 individuals imputed with the 1000 Genomes Project reveals an exonic damaging variant in ANGPTL4 determining fasting TG levels. J. Med. Genet. *53*, 441–449.
85. Shin, S., Hudson, R., Harrison, C., Craven, M., and Keles, S. (2018). atSNP Search: a web resource for statistically evaluating influence of human genetic variation on transcription factor binding. Bioinformatics. Published online December 8, 2018. https://doi.org/10.1093/bioinformatics/bty1010.
86. Buckley, M.T., Racimo, F., Allentoft, M.E., Jensen, M.K., Jonsson, A., Huang, H., Hormozdiari, F., Sikora, M., Marnetto, D., Eskin, E., et al. (2017). Selection in Europeans on Fatty Acid Desaturases Associated with Dietary Changes. Mol. Biol. Evol. *34*, 1307–1318.
87. Mathieson, I., Lazaridis, I., Rohland, N., Mallick, S., Patterson, N., Roodenberg, S.A., Harney, E., Stewardson, K., Fernandes, D., Novak, M., et al. (2015). Genome-wide patterns of selection in 230 ancient Eurasians. Nature *528*, 499–503.
88. Howard, T.D., Mathias, R.A., Seeds, M.C., Herrington, D.M., Hixson, J.E., Shimmin, L.C., Hawkins, G.A., Sellers, M., Ainsworth, H.C., Sergeant, S., et al. (2014). DNA methylation in an enhancer region of the FADS cluster is associated with FADS activity in human liver. PLoS ONE *9*, e97510.
89. Tung, Y.C.L., Yeo, G.S.H., O'Rahilly, S., and Coll, A.P. (2014). Obesity and FTO: Changing Focus at a Complex Locus. Cell Metab. *20*, 710–718.
90. Charrière, S., Peretti, N., Bernard, S., Di Filippo, M., Sassolas, A., Merlin, M., Delay, M., Debard, C., Lefai, E., Lachaux, A., et al. (2011). GPIHBP1 C89F neomutation and hydrophobic C-terminal domain G175R mutation in two pedigrees with severe hyperchylomicronemia. J. Clin. Endocrinol. Metab. *96*, E1675–E1679.
91. Hambleton, S., Salem, S., Bustamante, J., Bigley, V., Boisson-Dupuis, S., Azevedo, J., Fortin, A., Haniffa, M., Ceron-Gutierrez, L., Bacon, C.M., et al. (2011). IRF8 mutations and human dendritic-cell immunodeficiency. N. Engl. J. Med. *365*, 127–138.
92. Hegele, R.A., Little, J.A., and Connelly, P.W. (1991). Compound heterozygosity for mutant hepatic lipase in familial hepatic lipase deficiency. Biochem. Biophys. Res. Commun. *179*, 78–84.
93. Patrick, A.D., and Lake, B.D. (1969). Deficiency of an acid lipase in Wolman's disease. Nature *222*, 1067–1068.
94. Costas, J. (2018). The highly pleiotropic gene SLC39A8 as an opportunity to gain insight into the molecular pathogenesis of schizophrenia. Am. J. Med. Genet. B. Neuropsychiatr. Genet. *177*, 274–283.
95. Johansen, C.T., Kathiresan, S., and Hegele, R.A. (2011). Genetic determinants of plasma triglycerides. J. Lipid Res. *52*, 189–206.
96. Zhang, H., and Reilly, M.P. (2017). *LIPA* Variants in Genome-Wide Association Studies of Coronary Artery Diseases: Loss-of-Function or Gain-of-Function? Arterioscler. Thromb. Vasc. Biol. *37*, 1015–1017.
97. Wild, P.S., Zeller, T., Schillert, A., Szymczak, S., Sinning, C.R., Deiseroth, A., Schnabel, R.B., Lubos, E., Keller, T., Eleftheriadis, M.S., et al. (2011). A genome-wide association study identifies LIPA as a susceptibility gene for coronary artery disease. Circ Cardiovasc Genet *4*, 403–412.
98. Hess, J.L., Tylee, D.S., Barve, R., de Jong, S., Ophoff, R.A., Kumarasinghe, N., Tooney, P., Schall, U., Gardiner, E., Beveridge, N.J., et al.; Neurobehavioural Genetics Unit (2016). Transcriptome-wide mega-analyses reveal joint dysregulation of immunologic genes and transcription regulators in brain and blood in schizophrenia. Schizophr. Res. *176*, 114–124.
99. Lander, E.S., Linton, L.M., Birren, B., Nusbaum, C., Zody, M.C., Baldwin, J., Devon, K., Dewar, K., Doyle, M., FitzHugh, W., et al.; International Human Genome Sequencing Consortium (2001). Initial sequencing and analysis of the human genome. Nature *409*, 860–921.
100. ENCODE Project Consortium (2004). The ENCODE (ENCyclopedia Of DNA Elements) Project. Science *306*, 636–640.

The American Journal of Human Genetics, Volume *105*

Supplemental Data

Genetic and Epigenetic Fine Mapping of Complex Trait Associated Loci in the Human Liver

Minal Çalışkan, Elisabetta Manduchi, H. Shanker Rao, Julian A. Segert, Marcia Holsbach Beltrame, Marco Trizzino, YoSon Park, Samuel W. Baker, Alessandra Chesi, Matthew E. Johnson, Kenyaita M. Hodge, Michelle E. Leonard, Baoli Loza, Dong Xin, Andrea M. Berrido, Nicholas J. Hand, Robert C. Bauer, Andrew D. Wells, Kim M. Olthoff, Abraham Shaked, Daniel J. Rader, Struan F.A. Grant, and Christopher D. Brown

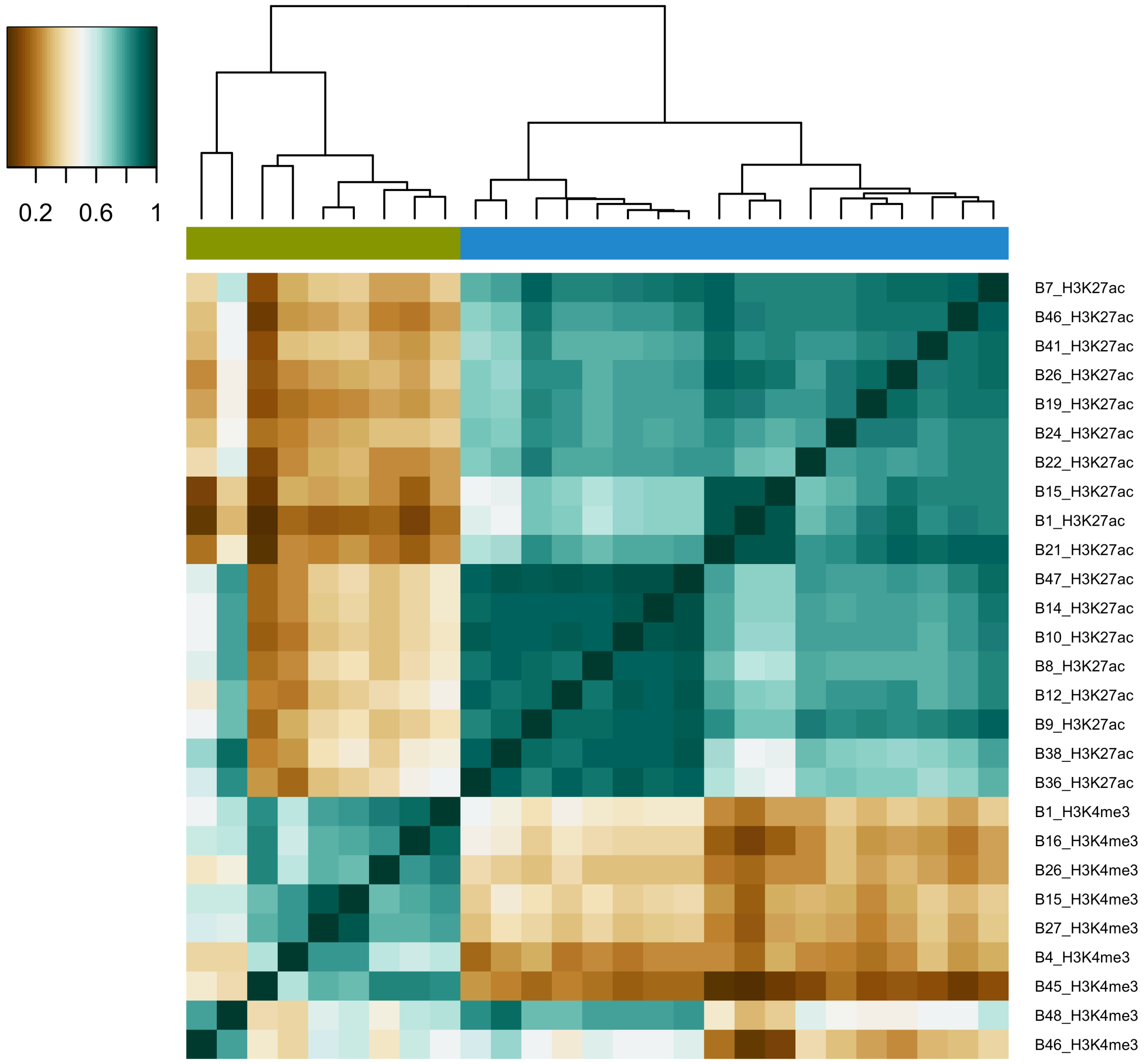

Figure S1. Heatmap plot of Spearman's correlation of ChIP-Seq data. Color bars above the heatmap plot indicate the ChIP-seq data type; columns corresponding to H3K4me3 data are shown in green, columns corresponding to H3K27ac data are shown in blue. Subject IDs are included as row labels.

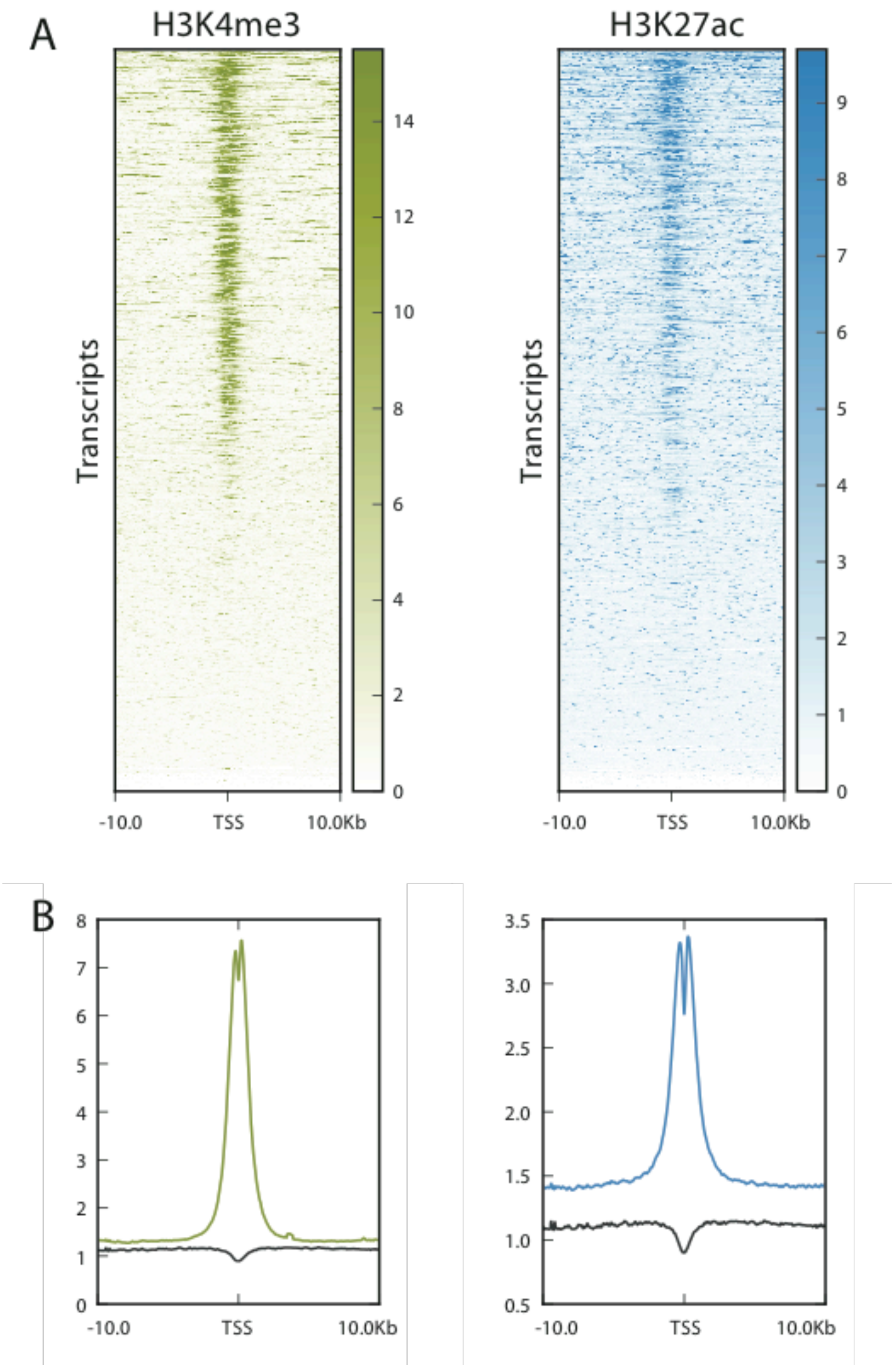

Figure S2. A) Heatmap plots of ChIP-Seq read counts in 20 kb regions centered on GENCODE v19 transcript TSS positions **B)** Profile plots of ChIP and Input sequencing read counts centered on GENCODE v19 transcript TSS positions. Green line corresponds to H3K4me3, blue line corresponds to H3K27ac, and black lines correspond to their Input DNA data.

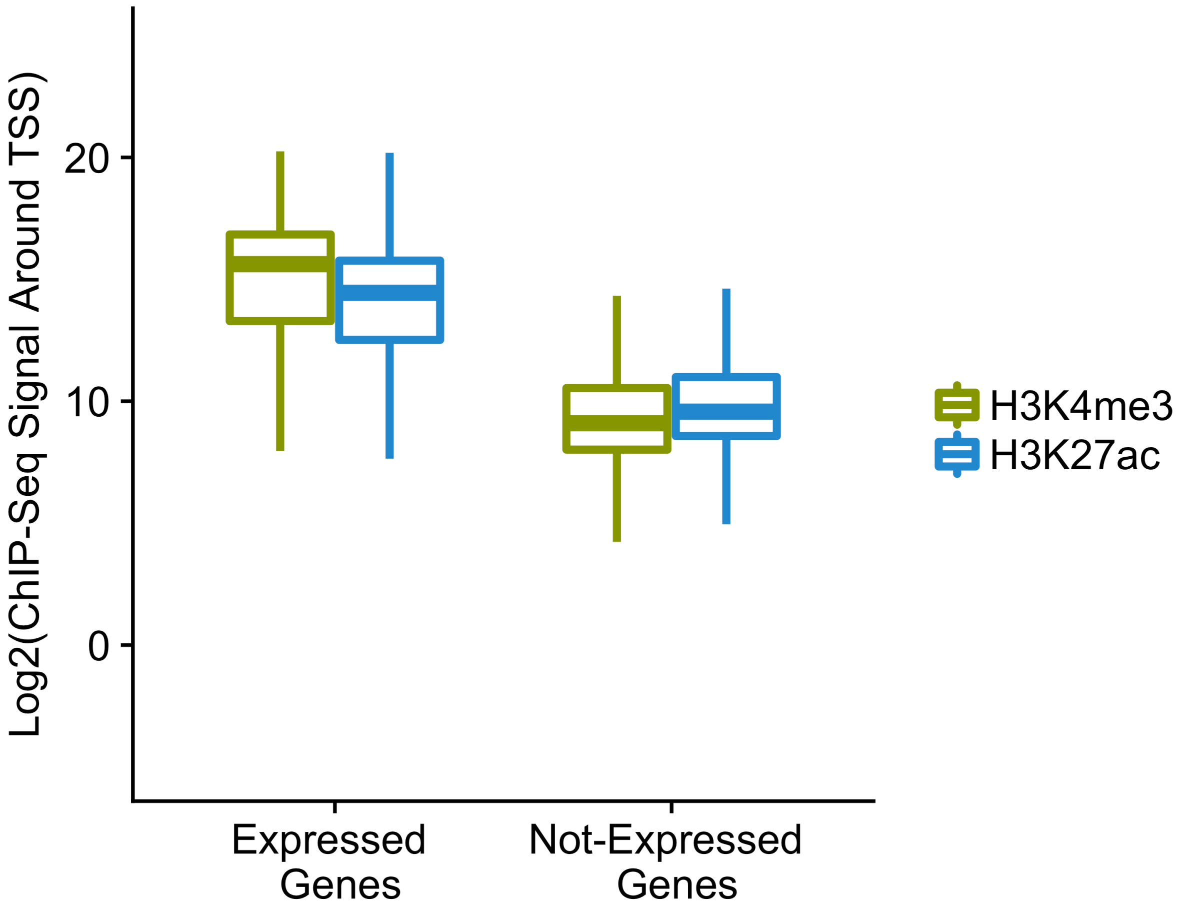

Figure S3. H3K4me3 and H3K27ac ChIP-Seq read counts in 2 kb regions centered on GENCODE v19 transcript TSS positions were extracted from merged ChIP-Seq data of biological replicates (N=9 for H3K4me3 and N=18 for H3K27ac). ChIP-Seq read counts around TSS of the 19,133 expressed genes were significantly higher than that of 38,687 genes that were not detected as expressed in Penn Cohort 1.

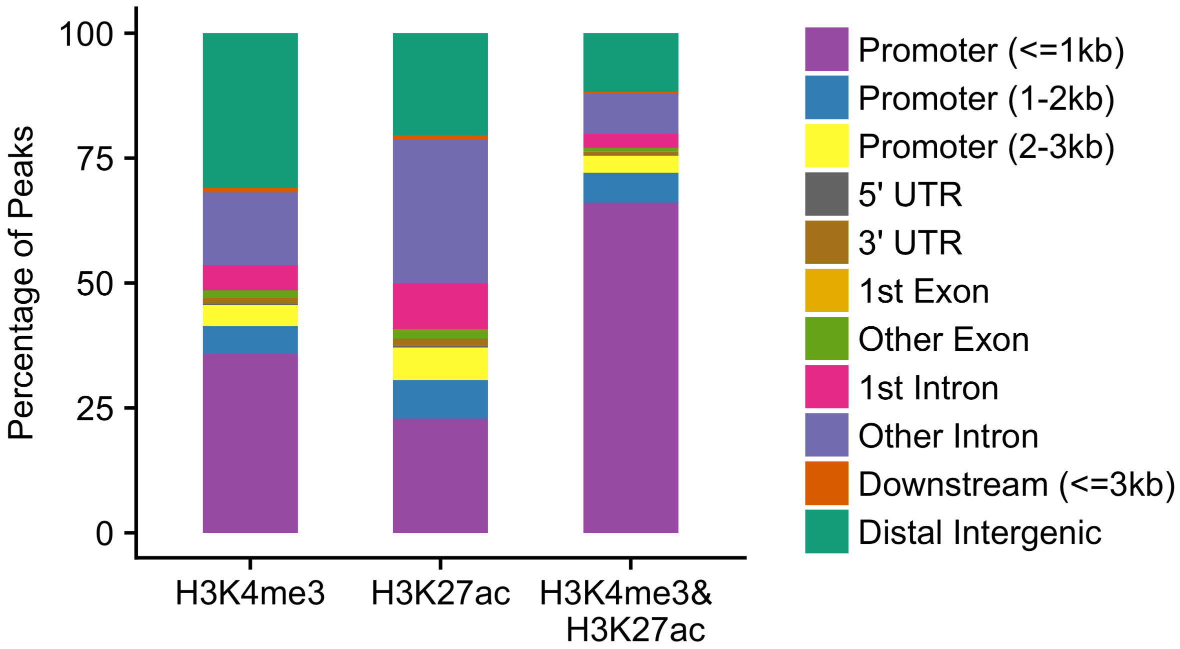

Figure S4. Genomic annotations of the ChIP-Seq peaks. Annotations are displayed for 68,600 H3K4me3 peaks, 131,293 H3K27ac peaks and 28,167 overlapping peaks between H3K4me3 and H3K27ac datasets.

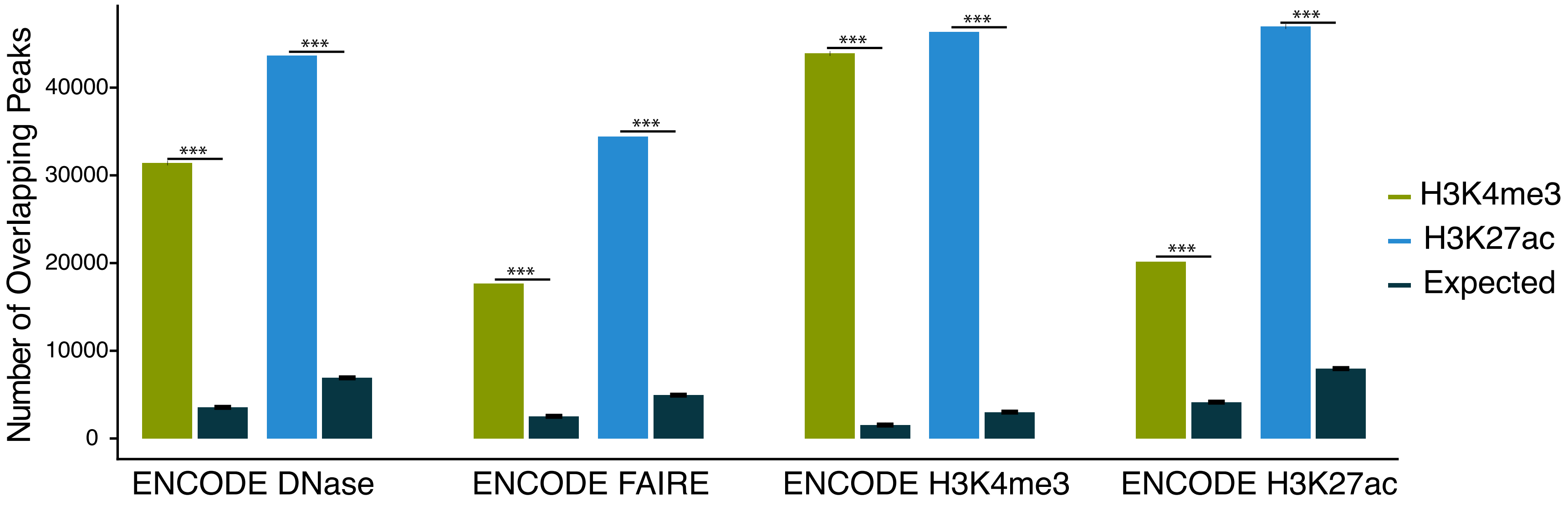

Figure S5. Observed and expected numbers of H3K4me3 and H3K27ac peaks overlapping ENCODE datasets in HepG2 cells. Expected numbers represent the mean overlap based on 1,000 sets of randomly selected size-matching regions for each of the original peaks. Error bars represent standard error of the mean. One-tailed Fisher's exact test P-values were $<2.2 \times 10^{-16}$ for all comparisons.

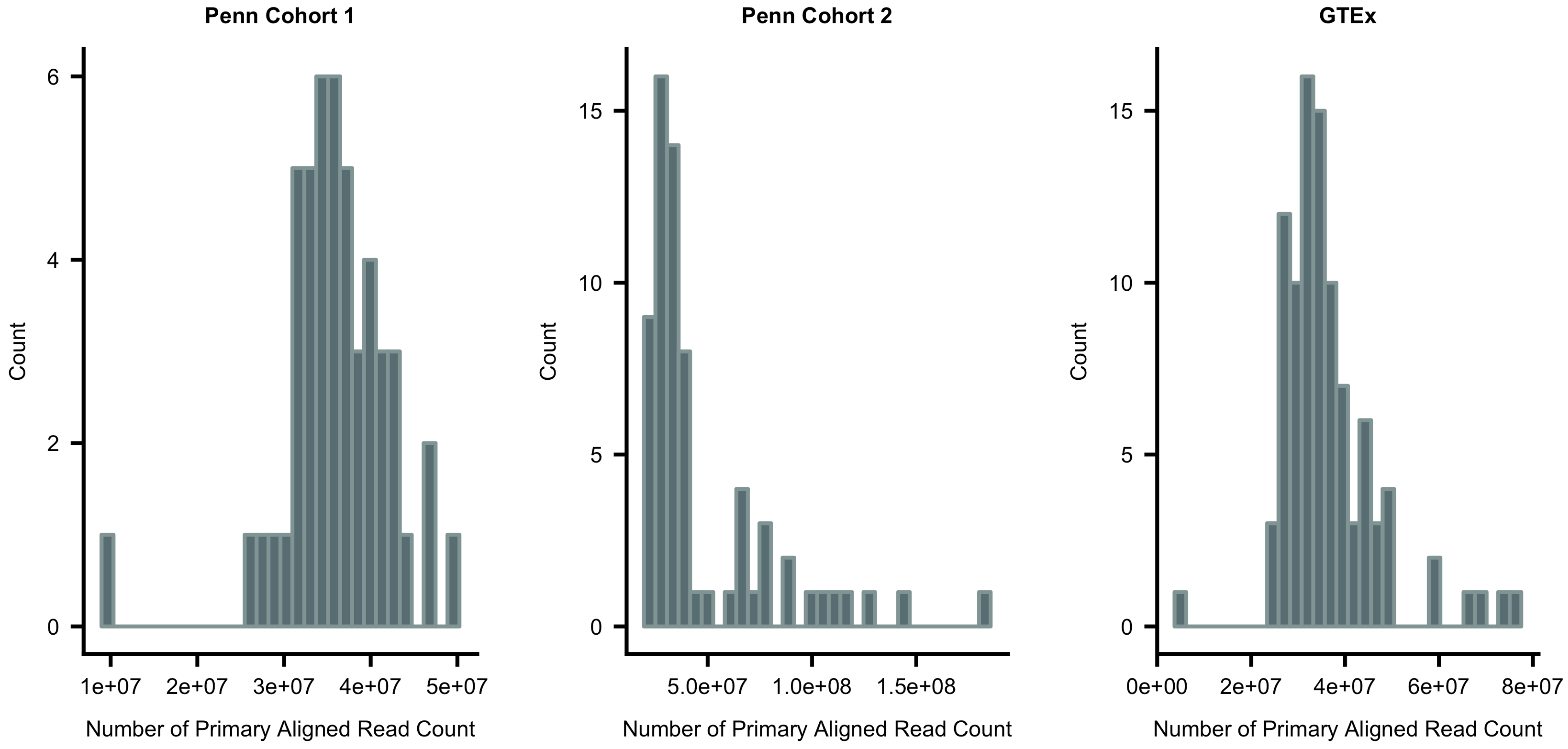

Figure S6. Histogram of the number of primary aligned RNA-seq reads to the reference human genome (hg19). Median read counts were 35.86, 32.72, 33.74 million for Penn Cohort 1, Penn Cohort 2, and GTEx, respectively.

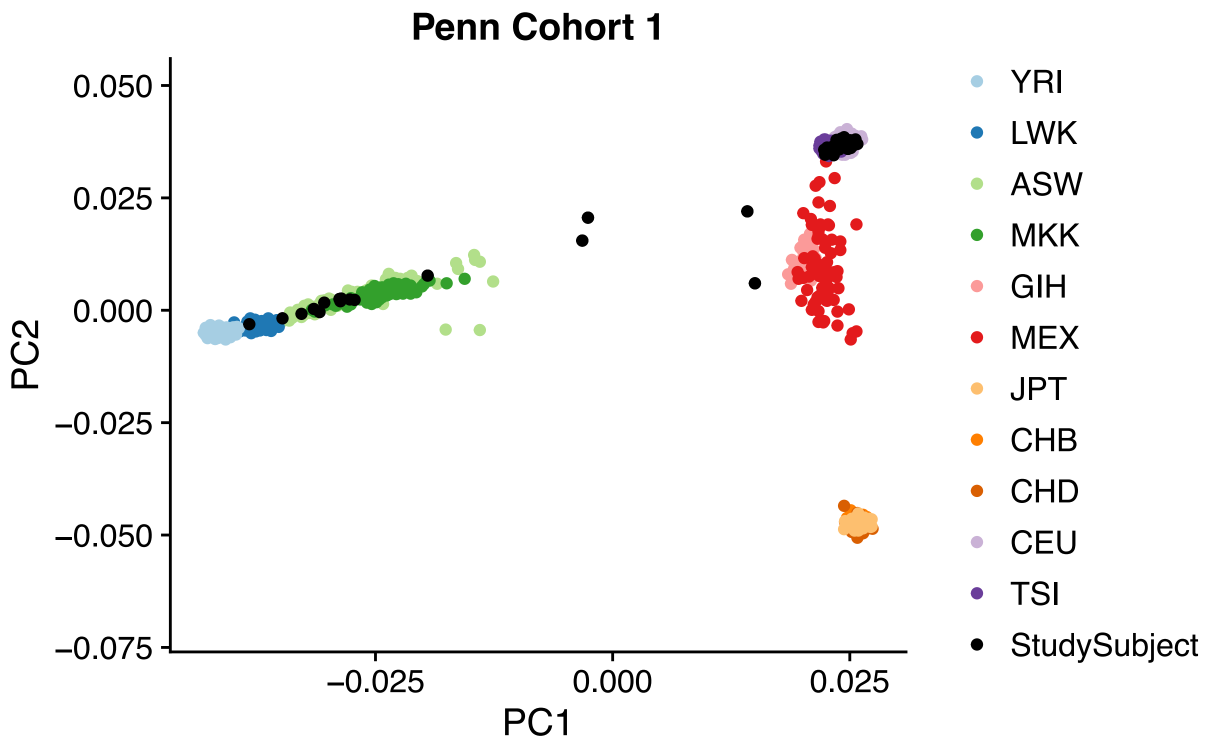

Figure S7. PCA plot of the genotype data of Penn Cohort 1 individuals. Plot displays the first two principal components of LD pruned genotype data from 50 study subjects (shown in black) with 1,184 HapMap Phase 3 individuals representing 11 populations. 34 of 50 individuals clustered with the HapMap European populations, 12 of them clustered with the HapMap African populations, and the remaining 4 individuals displayed mixed genetic ancestry.

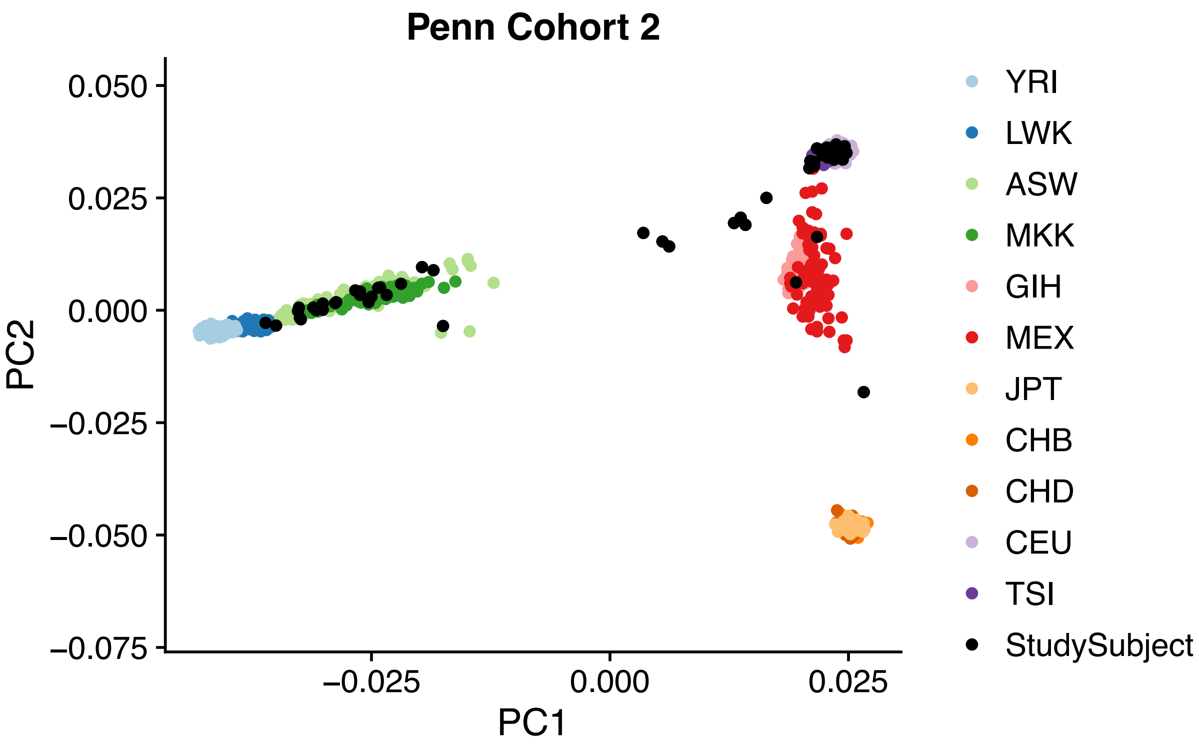

Figure S8. PCA plot of the genotype data of Penn Cohort 2 individuals. Plot displays the first two principal components of LD pruned genotype data from 96 study subjects (shown in black) with 1,184 HapMap Phase 3 individuals representing 11 populations. 62 of 96 individuals clustered with the HapMap European populations, 24 of them clustered with the HapMap African populations, and the remaining 10 individuals displayed mixed genetic ancestry.

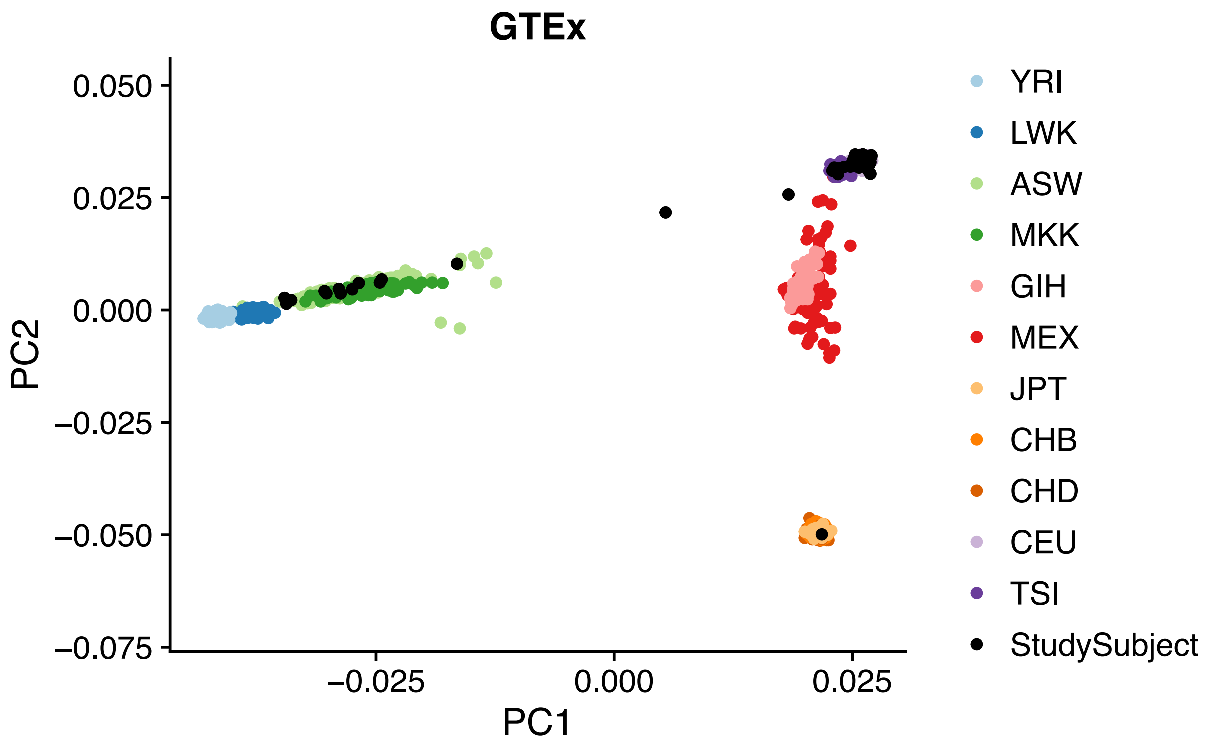

Figure S9. PCA plot of the genotype data of GTEx individuals. Plot displays the first two principal components of LD pruned genotype data from 96 study subjects (shown in black) with 1,184 HapMap Phase 3 individuals representing 11 populations. 81 of 96 individuals clustered with the HapMap European populations, 12 of them clustered with the HapMap African populations, one individual clustered with the HapMap Asian populations and the remaining two individuals displayed mixed genetic ancestry.

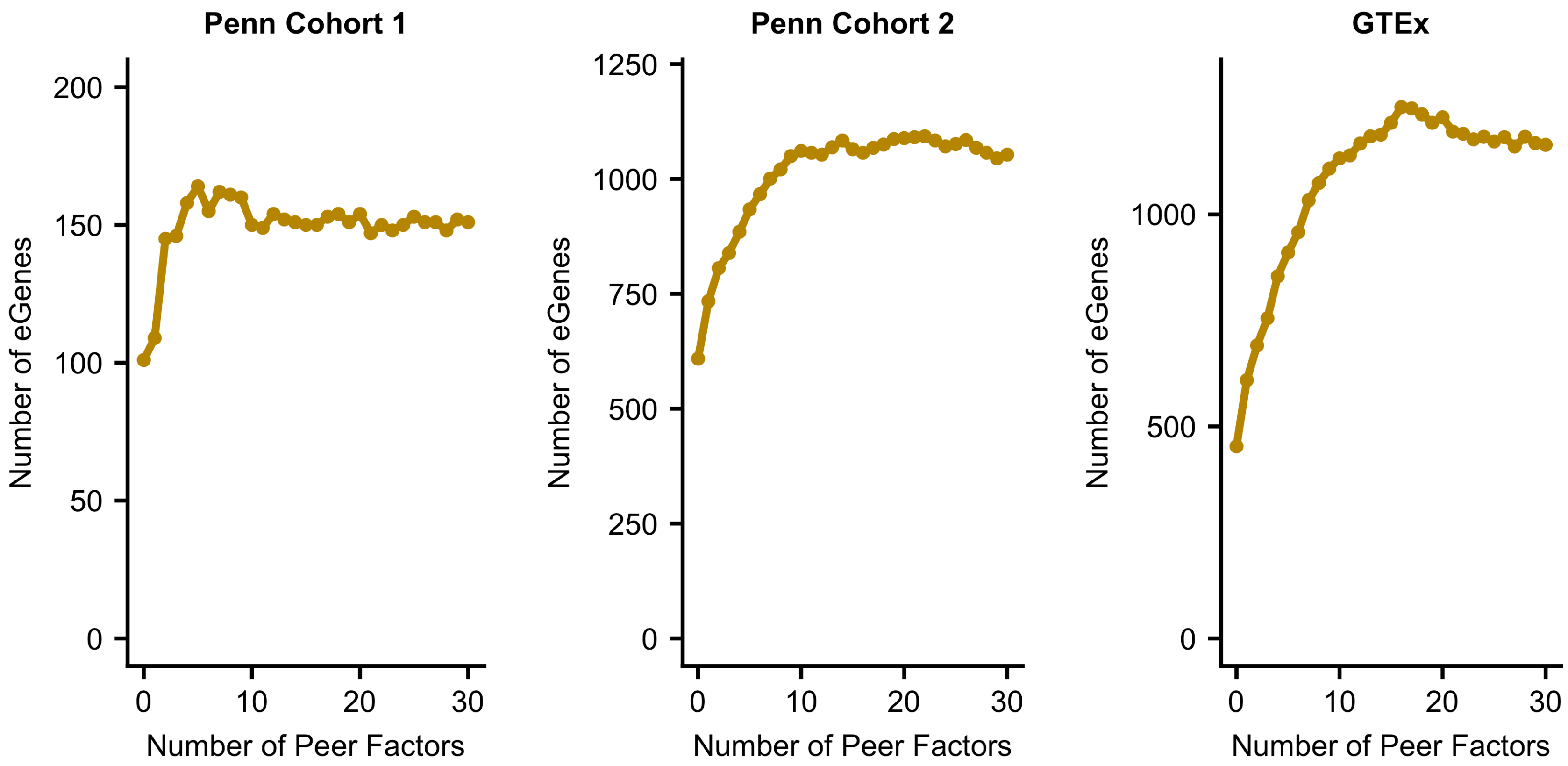

Figure S10. Number of eQTL-Genes identified as a function of number of Peer factor covariates that were included in cis-eQTL mapping (based on FastQTL). Number of eQTL-Genes were maximized when 5, 22, and 16 Peer Factors were used for Penn Cohort 1, Penn Cohort 2, and GTEx, respectively.

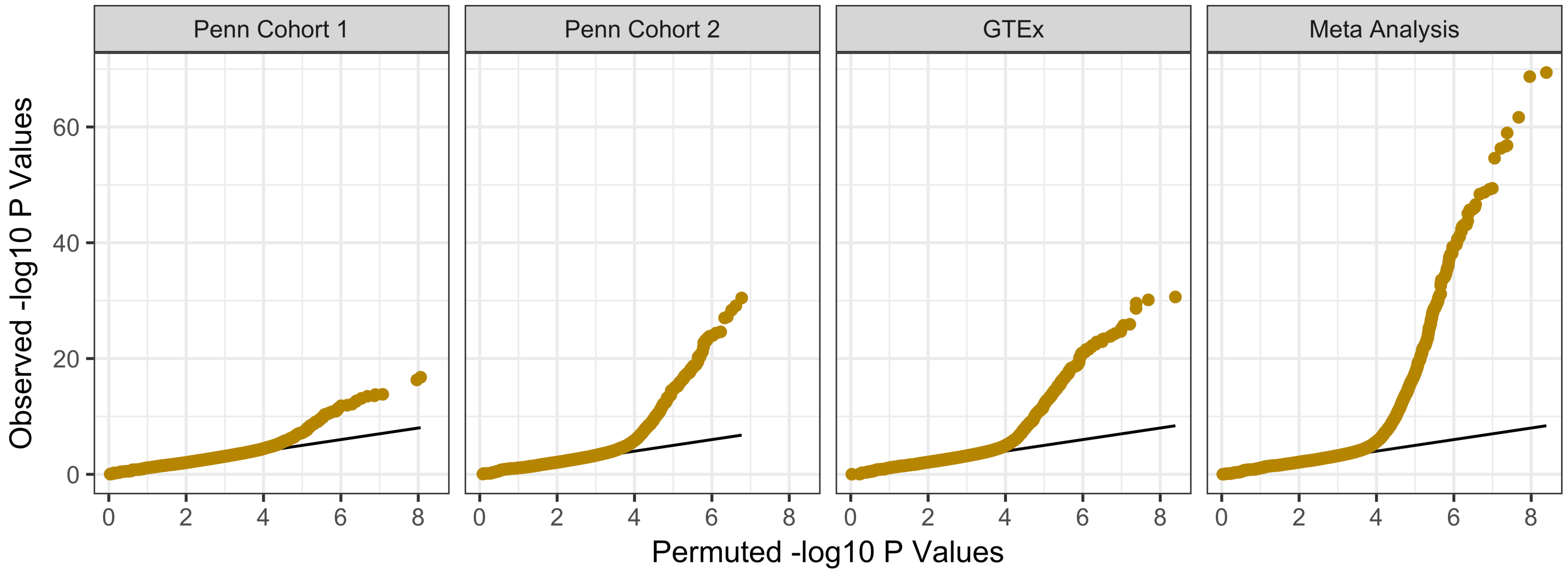

Figure S11. QQ-plots of the cis-eQTL association P-values within each cohort (Panels 1 to 3) and meta P-values across cohorts (Panel 4). Solid lines represent the distribution of P-values based on permuted data. A total of 2,625 cis eQTL-Genes were identified based on meta-analysis eQTL results. See Figure 1 for numbers of significant results within each cohort.

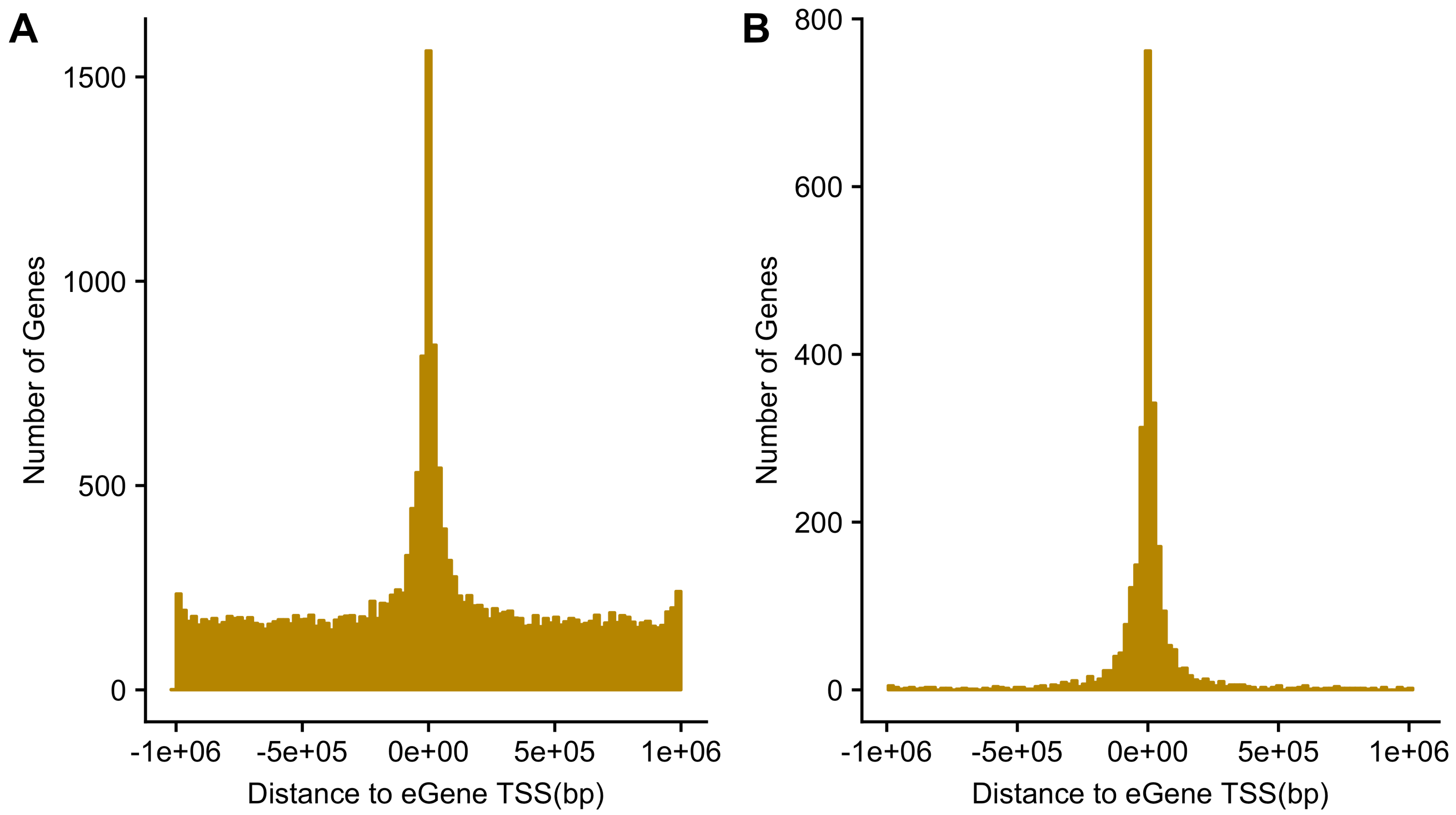

Figure S12. Lead SNP – target gene distance based on **A)** All genes **B)** Genes with significant meta cis-eQTLs

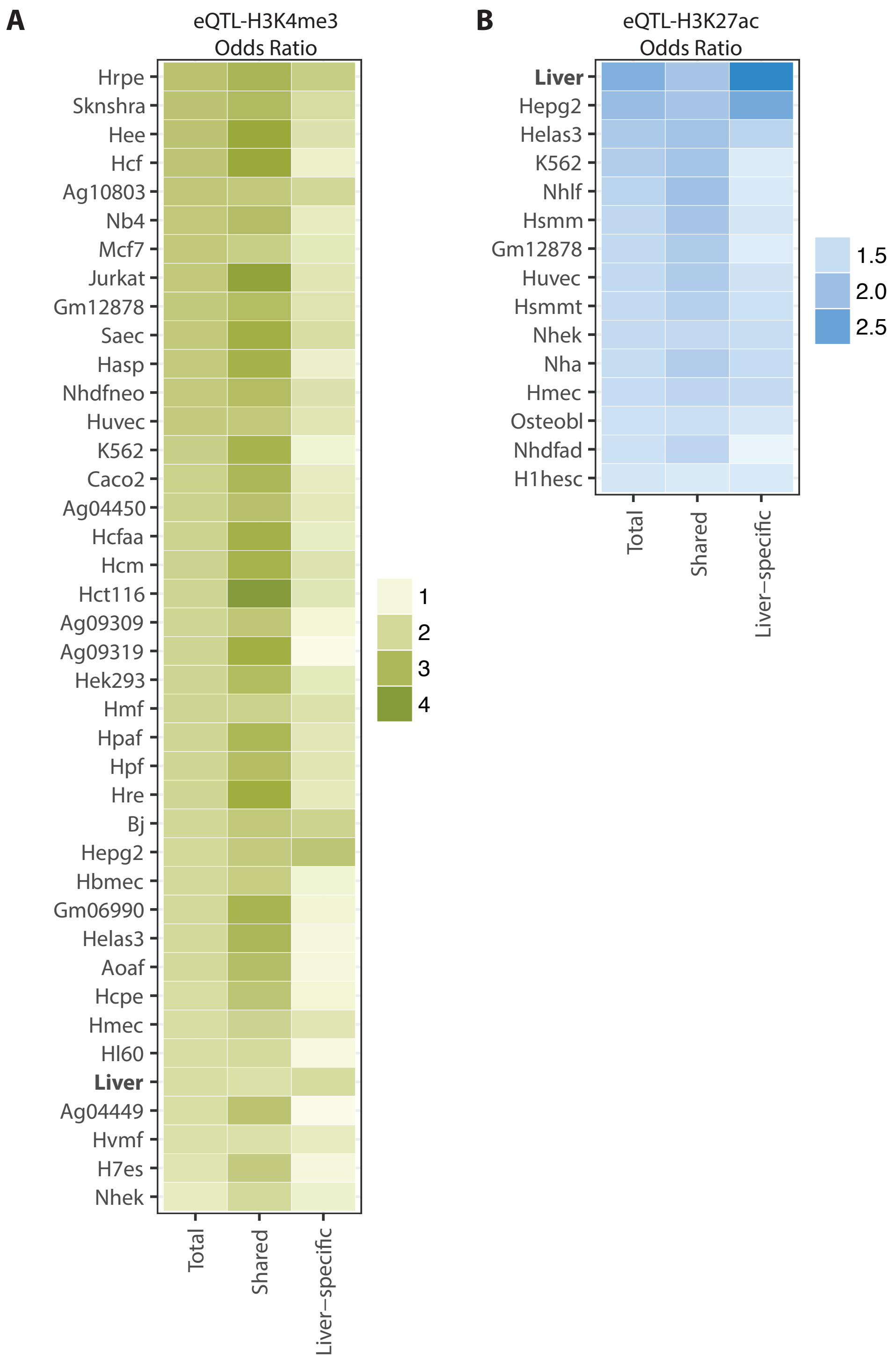

Figure S13. A) Odds ratios of overlap between H3K4me3 datasets and total, shared, and liver-specific cis-eQTLs **B)** Odds ratios of overlap between H3K27ac datasets and total, shared, and liver specific cis-eQTLs.

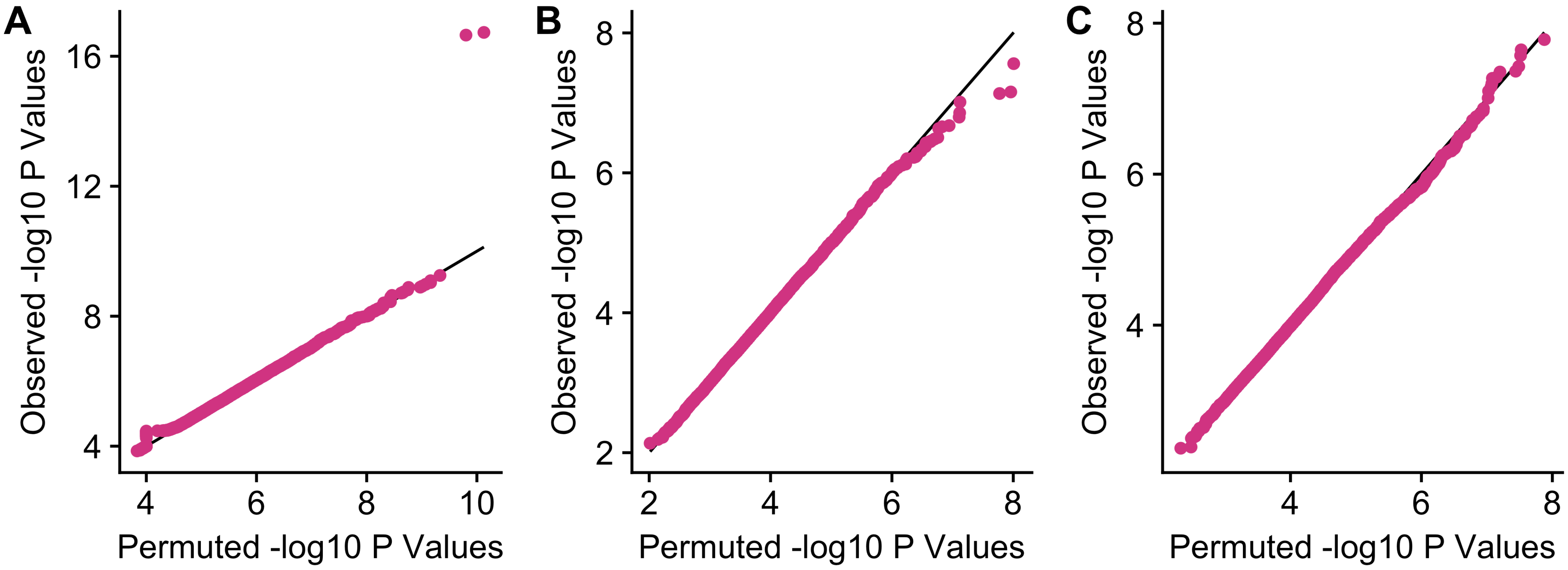

Figure S14. QQ-plots of the meta trans-eQTL association P-values using **A)** all linkage disequilibrium pruned variants in the genome **B)** cis-eQTL variants **C)** variants likely to affect activity of transcription factors. Magenta points correspond to observed P-values. Solid lines represent the expected distribution of P-values based on permuted gene expression data.

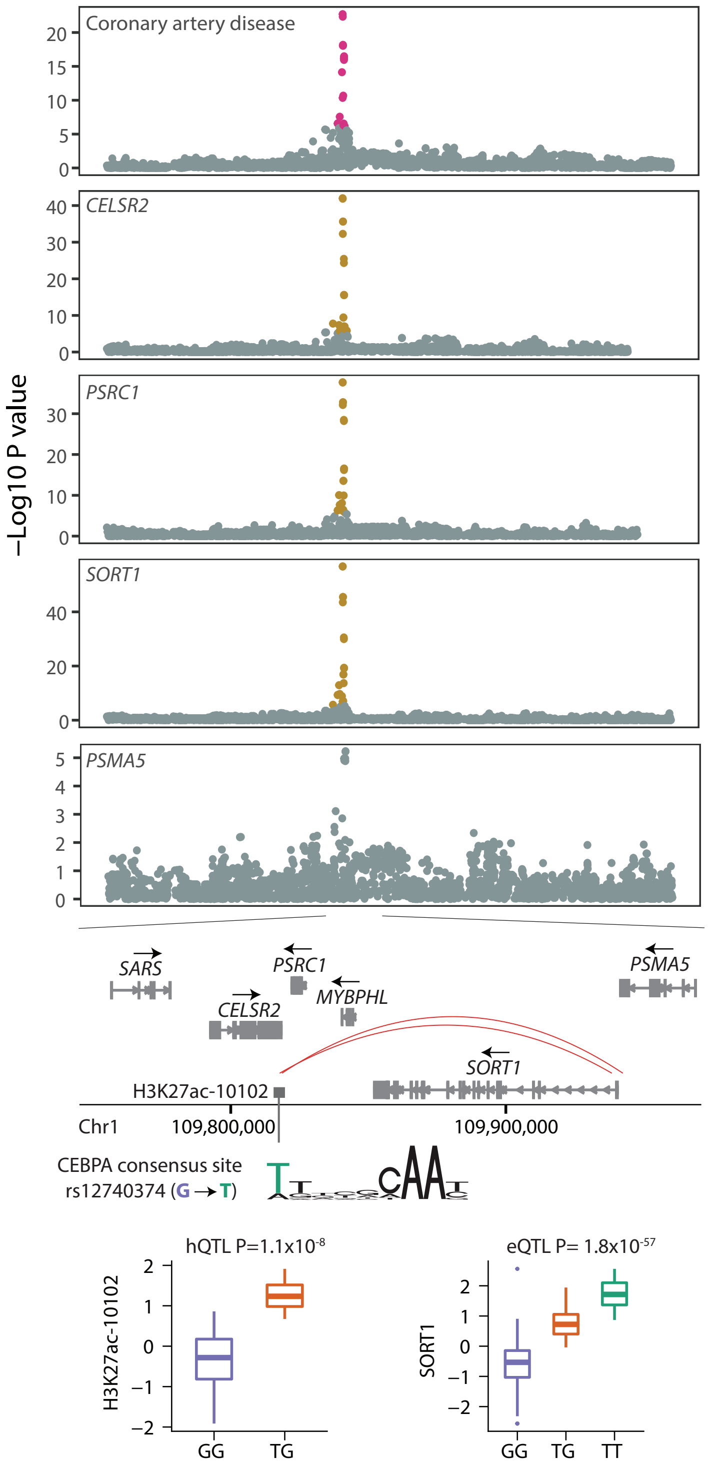

Figure S15. Significant colocalization signals at the chromosome 1p13.3 locus. Colocalization posterior probabilities of coronary artery disease associations with *CELSR2*, *PSRC1*, *SORT1* and *PSMA5* gene expression levels were 0.999, 0.999, 0.999, and 0.947,

respectively. Schematic representation of the genes in the zoomed-in locus of chr1: 109,750,000-109,940,573 and the putatively causal H3K27ac-10102 peak (chr1: 109,816,977-109,818,871). H3K27ac-10102 peak only formed DNA looping interaction with the promoter of the *SORT1* gene in the genome (CHiCAGO scores: 6.63 and 7.8). The T allele of the candidate causal variant, rs12740374, increases the odds of CEBPA binding. Box plots of normalized H3K27ac-10102 ChIP-Seq and *SORT1* RNA-Seq read counts are stratified by genotype at the rs12740374. Sample sizes of each genotype group were GG:14, TG:4 for ChIP-Seq data and GG:143, TG:83, TT:14 for RNA-Seq data.

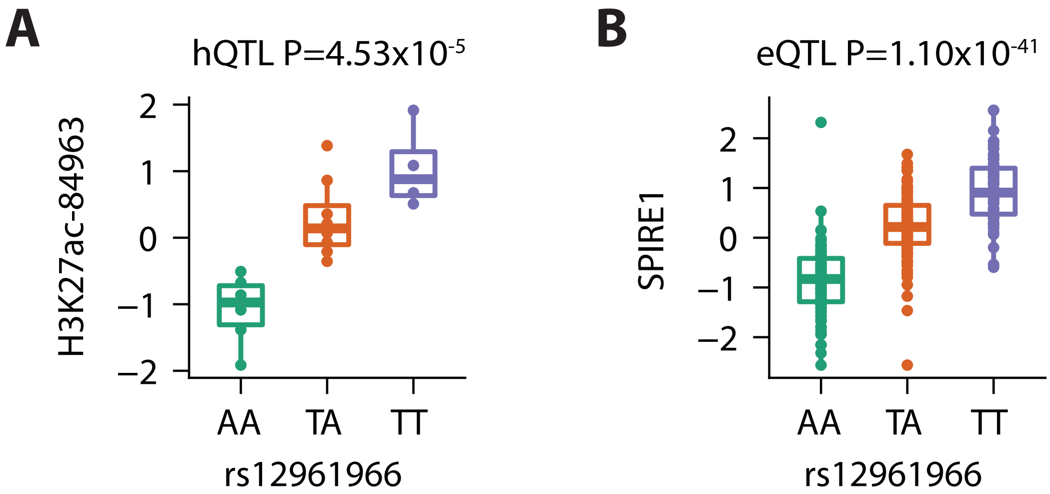

Figure S16. An example of putatively co-regulated histone modification and gene expression levels. Box plots of normalized H3K27ac-84963 ChIP-Seq and *SPIRE1* RNA-Seq read counts are stratified by genotype at the rs12961966. Sample sizes of each genotype group were TT:4, AT:8, AA:5 for ChIP-Seq data and TT:55, AT:94, AA:88 for RNA-Seq data.
